# Analysis of Antibiotic Consumption Trends and Pathogens’ Epidemiological Profile Within a Multidisciplinary Clinical Hospital from Romania

**DOI:** 10.3390/antibiotics15030288

**Published:** 2026-03-12

**Authors:** Andreea-Roxana Ungureanu, Andreea-Alina Dumitru, Emma-Adriana Ozon, Andrei-Tudor Rogoz, Raluca-Narcisa Anghel, Elena Ciucu, Ancuța-Cătălina Fița, Nicoleta-Mirela Blebea

**Affiliations:** 1 Department of Pharmacy, Sanador Clinical Hospital, 010991 Bucharest, Romania; andreea.ungureanu@sanador.ro (A.-R.U.); raluca.anghel@sanador.ro (R.-N.A.); elena.ciucu@sanador.ro (E.C.); 2 Department of Pharmaceutical Technology and Biopharmacy, Faculty of Pharmacy, “Carol Davila” Univesity of Medicine and Pharmacy, 020945 Bucharest, Romania; emma.budura@umfcd.ro (E.-A.O.); catalina.fita@umfcd.ro (A.-C.F.); 3 Department of Microbiology, Hospital Laboratory, Sanador Clinical Hospital, 010991 Bucharest, Romania; 4 Department of Healthcare-Associated Infections, Sanador Clinical Hospital, 010991 Bucharest, Romania; andrei.rogoz@sanador.ro; 5 Department of Pharmacotherapy, Faculty of Pharmacy, Ovidius University of Constanța, 900001 Constanța, Romania; nicoleta.blebea@365.univ-ovidius.ro

**Keywords:** antimicrobial resistance, antibiotic therapy, defined daily dose, access, watch, reserve, ESKAPE, hospital

## Abstract

**Background/Objectives:** In the broad and current context of antimicrobial resistance, antibiotic management and therapeutic surveillance are essential in hospitals. The present study (five-year retrospective, 2020–2024) aimed to analyze antibiotic consumption in relation to pathogens identified in a multidisciplinary hospital. **Results:** In terms of antibiotic consumption (overall 2020–2024), although initially Watch antibiotics were predominantly used, a decrease was observed in favor of Access class antibiotics (sharply increase from 2022 to 2023 and maximum in 2024). For Reserve antibiotics, only slight annual fluctuations were observed, but there was an important reduction in colistin consumption. The most used were cephalosporins (cefazolin, cefuroxime and ceftriaxone), carbapenems (meropenem and ertapenem), vancomycin and linezolid. Regarding pathogens, the most notable were: *Staphylococcus aureus*, *Escherichia coli*, *Klebsiella pneumoniae*, *Enterococcus* spp., *Pseudomonas aeruginosa*. Among the ESKAPE bacteria, *Acinetobacter baumannii* was the least frequent in our samples. ESKAPE bacteria predominantly colonized specimens from the respiratory tract, digestive tract, skin and soft tissue. Resistant strains were observed, mainly Methicillin-resistant *Staphylococcus aureus* (MRSA) and Extended-Spectrum Beta-Lactamase (ESBL) *Klebsiella* spp., but no alarming increases in number were recorded in the analyzed period. **Methods:** The analysis was carried out using tools recommended by the World Health Organisation (Access Watch Reserve antibiotics classification (AWaRe); Bacterial Priority Pathogen List (BBPL); Defined Daily Dose (DDD)), Average Annual Percent Change (AAPC) calculation and ESKAPE classification (bacteria group: *Enterococcus faecium*, *Staphylococcus aureus*, *Klebsiella pneumoniae*, *Acinetobacter baumannii*, *Pseudomonas aeruginosa* and *Enterobacter* spp.). **Conclusions:** Relatively stable trends in bacterial isolates and resistant strains over five years (2020–2024) are consistent with effective antimicrobial stewardship practices.

## 1. Introduction

The development of antimicrobial resistance (AMR) represents a complex evolutionary process that has gained more and more attention in recent years (regarded as “silent pandemic”) [1]. As it escalates, once-treatable infections become increasingly difficult or even impossible to manage, undermining decades of medical progress. The spread of resistant pathogens places a substantial burden on healthcare systems and increases mortality rates; it is estimated that by 2050, there will be an increase of approximately ten million deaths per year due to AMR, especially in low/middle-income countries [2,3,4]. Superimposed on this ongoing thread, between 2020 and 2023, humanity faced the Coronavirus Disease 2019 (COVID-19) pandemic. During this period, antimicrobials and biocides have been used in large amounts, often inappropriately [5,6]. Moreover, patients with assisted ventilation received multiple antibiotics with an impact on respiratory infectious pathogens (such as *Klebsiella pneumoniae*, *Pseudomonas aeruginosa*, *Acinetobacter baumannii*, *Staphylococcus aureus*), contributing to the risk of promoting AMR [7,8,9].

The World Health Organization (WHO) is actively involved in improving global antibiotic stewardship. The AWaRe classification was established by the WHO in 2017 as a strategic framework for optimizing antibiotic use and promoting antimicrobial stewardship at global, national and institutional levels [10]. Briefly, Access antibiotics are narrow-spectrum agents and generally have low resistance potential, in contrast with Watch antibiotics (broad-spectrum and generally used for infections determined by pathogens that are more likely to be resistant to the Access class). Reserve antibiotics are used to treat multidrug-resistant infections (as last-resort options). This grading facilitates standardized surveillance of antibiotic consumption and supports evidence-based policy development to mitigate antimicrobial resistance globally. Also, the WHO measures include awareness in antibiotic development (by establishing the Bacterial Priority Pathogen List—BPPL [11]) and usage (by AWaRe book [12]), which guide antibiotic selection and highlight critical resistance threats. Additionally, WHO has strengthened monitoring and reporting frameworks through tools like GLASS (Global Antimicrobial Resistance and Use Surveillance System), initiated in 2015, and DDD (Defined Daily Dose) methodology, enabling countries to track antibiotic consumption (AMC) and resistance patterns (AMR). According to the GLASS Enrolment Map (available from October 2024), Romania is enrolled only in AMC (from 2022). The reported values are aggregated data from both community and hospital use.

Even if a significant contribution to the development of antibiotic-resistant microorganisms comes from outpatient care, where patients administer the medication on their own and may not follow the prescribed regimen correctly (treatment adherence issues) [13,14], the hospital sector can be assumed as a central link in the chain of antimicrobial resistance. The intensive use of antibiotics in hospitals, where critically ill patients often require broad-spectrum and/or high-dose treatments, generates strong selective pressure for resistant strains to emerge and spread [15]. At the same time, hospitals rely on trained professionals who can develop and apply evidence-based protocols, closely monitor antibiotic use and implement effective surveillance systems to find and control emerging resistance patterns. In this dual role, the hospitals remain not only a high-risk cornerstone for resistance development but also a capable setting for its prevention by rigorous surveillance and assessment [16,17,18].

In this context, our study aims to analyze the antibiotic consumption and the epidemiological profile of pathogens identified in positive specimens within a hospital in Romania over a five-year period. This contributes to a correlation of interdepartmental results and helps us establish new directions for preventing antimicrobial resistance.

## 2. Results

### 2.1. Overall Antibiotic Consumption

**Figure 1:**
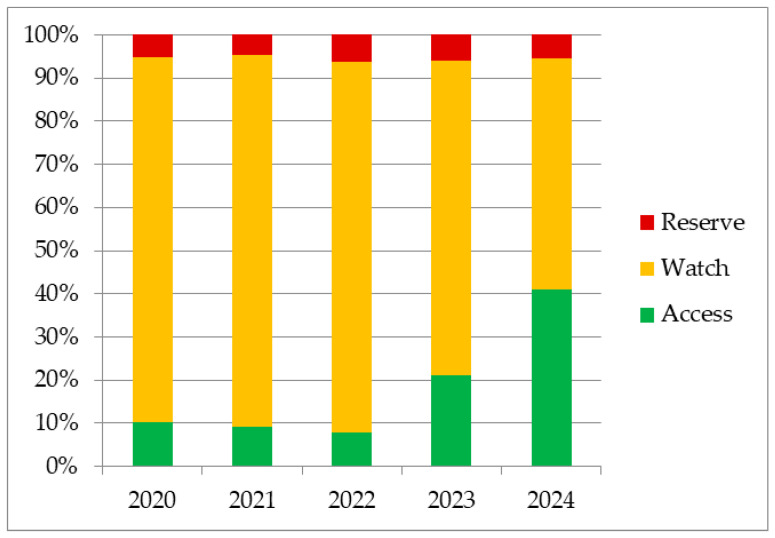
Antibiotic consumption grouped by AWaRe classification.

The variation of overall antibiotic consumption between 2020 and 2024, grouped by AWaRe classification, is shown in Figure 1, and the integration of the AWaRe classification system in hospitals’ antibiotic management is shown in Supplementary Materials (Figure S1). In the first three years of this study, Watch antibiotics outstandingly dominated (84.79%, 86.12%, 85.80%), but from 2023, the consumption of these began to decrease (2023: 73.06%; 2024: 53.63%). Regarding Reserve antibiotics, there are only slight annual fluctuations, with maximum consumption in 2022 (6.35%) and a slight decrease in the following years (2023: 5.96%; 2024: 5.34%). Between 2020 and 2022, a decrease in Access antibiotics was observed (2020: 10.15%; 2021: 9.27%; 2022: 7.58%), followed by a sharp increase in 2023 (20.98%) and 2024 (41.02%).

In 2024, the most balanced consumption was noticed for Watch and Access. Even though Watch antibiotics are the most used, the Access group showed a remarkably proportion, approximately two times higher than in 2023 and four times higher than in the first year of the analysis.

### 2.2. Access Group Consumption

The consumption of antibiotics in the Access category, as DDD/100 bed days, is presented in Table 1. The individual trend of each antibiotic and for the Access group was shown in Supplementary Materials (Figures S2–S16).

Between 2020 and 2022, aminoglycosides (predominantly amikacin) and penicillins (predominantly amoxicillin/clavulanic and ampicillin) were the most used. Since 2023, cefazolin has been introduced in the prophylaxis protocol; thus, a notable increase in consumption was observed, approximately double in 2024 compared to 2023. At the same time, in the last two years analyzed, the use of antibiotics from other classes decreased, especially aminoglycosides and penicillins. An important consumption was also observed for imidazolidines (metronidazole), especially for parenteral administration. For the Access group, a remarkably Average Annual Percent Change (AAPC) of 50.05 was noticed.

### 2.3. Watch Group Consumption

Watch antibiotic consumption is presented in Table 2 as DDD/100 bed days. In the carbapenem category, a decrease in ertapenem and imipenem was noticed, but there was an increase in the consumption of meropenem (positive AAPC). Regarding cephalosporins, second-generation molecules (cefuroxime) showed an important decrease, especially after substitution in the prophylaxis with cefazolin. For third-generation cephalosporins, a decrease in ceftazidime consumption was noticed (AAPC of −35.36), and an increase in ceftriaxone (AAPC of 31.10), which was outstanding in the last two years, was noticed. The trend for each antibiotic and for the Watch group is recorded in Supplementary Materials (Figures S17–S37).

Fluoroquinolone use was high in 2023 (predominantly parenteral levofloxacin and moxifloxacin), followed by a slow decrease in 2024. In the glycopeptides class, there was an upward trend due to vancomycin, even if teicoplanin showed a negative AAPC. Regarding macrolides, clarithromycin registered a higher consumption than azithromycin in the first years, but from 2022, the consumption reversed. Watch category penicillins (piperacillin) and phosphonics have not shown notable changes in consumption. As a general trend for the Watch group, a decrease was observed with an AAPC of −6.13, which was most significant during the last period.

### 2.4. Reserve Group Consumption

For the Reserve group, there were fewer antibiotic substances and lower DDD values (Table 3) compared to the Access and Watch groups. Consumption trends for each substance and for the entire group are presented in the Supplementary Materials (Figures S38–S46).

Among all, colistin, linezolid and tigecycline recorded considerable consumption with measurable year-to-year variation. Linezolid and tigecycline showed positive AAPC, with higher DDDs in 2022 and 2023, followed by a decrease in 2024. Colistin consumption was characterized by a negative AAPC, with high DDDs in 2022.

### 2.5. The Relative Frequency of Pathogens in Positive Samples

Bacterial identification was performed to the species level, depending on the characteristics of the isolates; results are provided in Table 4. The overall top five most frequently identified etiological agents in the positive samples were as follows: staphylococci species (mainly *S. aureus*) > *Escherichia coli* > *Klebsiella* species (especially *K. pneumoniae*) > *Enterococci* spp. (predominantly *E. faecalis* and *E. faecium*) > *Pseudomonas* species (mostly *P. aeruginosa*).

Among the identified pathogens, resistant strains were observed, which are presented in Table 5. An overall high proportion of 25.74% was registered for methicillin-resistant *Staphylococcus aureus* (MRSA) within *S. aureus* isolates, but without meaningful differences from year to year. Vancomycin-resistant enterococci (VRE) were observed predominantly in the last year (10 isolates—14.08%). The number of *Acinetobacter baumannii* isolates has varied each year, as well as for the Multidrug-Resistant (MDR) category, but since 2022, they have started to decrease.

Regarding ESBL-producing microorganisms, *Klebsiella* spp. stood out with a high proportion, which was most remarkable in 2020 (96 isolates—55.17%). A few ESBL-producing *Enterobacter* strains were also identified. *Escherichia coli* showed a considerable number of ESBL isolates each year (approximately 30). During 2020–2024, the prevalence of ESBL among *E. coli* isolates was 18.44%. For *Pseudomonas* spp., the most representative category of resistance was Carbapenem-Resistant (CR), with the highest proportions in 2022 and 2023.

### 2.6. ESKAPE Pathogens Across Clinical Specimens

**Figure 2:**
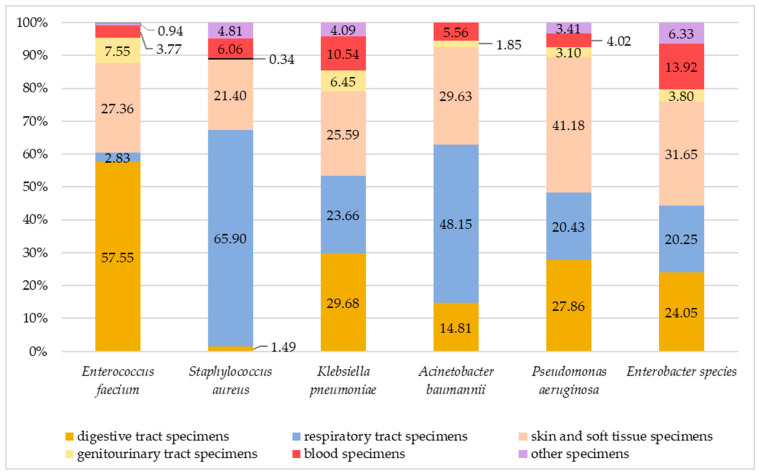
Distribution of positive samples for each ESKAPE pathogen (overall 2020–2024).

The distribution of sample types among positive samples for each ESKAPE pathogen is represented in Figure 2, for the cumulative period of 2020–2024 and for each year in Supplementary Materials (Figures S47–S51). Among the *Enterococcus faecium-positive* samples, most came from the digestive tract, followed by the skin and soft tissue. *Staphylococcus aureus* is most frequently isolated in samples from the respiratory system, followed by the skin and soft tissue. For *Klebsiella pneumoniae*-positive samples, similar percentages were found for respiratory, digestive, skin and soft tissue specimens. For *Acinetobacter baumannii*, an important percentage of positive samples came from the respiratory tract. *Pseudomonas aeruginosa* was mainly found in skin and soft tissue specimens, followed by respiratory and digestive tract samples, as well as for *Enterobacter species*. ESKAPE bacteria also colonized blood samples, which is significant for the clinical importance of systemic infections.

## 3. Discussion

The antibiotic use analysis over the five-year period (between 2020 and 2024) in a multidisciplinary hospital from Romania showed high use of Watch antibiotics in the first years, followed by an increase in the Access category in recent years. Other studies highlighted the same trend: an increase in Access (approximately +18%) and a decrease in Watch (approximately −12%) [19]. In our case this is attributed to a change in the prophylaxis protocol. This was decided after consumption patterns (tracked by DDD reports) revealed high use of Watch antibiotics. Thus, in 2023, cefuroxime (Watch-group second-generation cephalosporin) was replaced with cefazolin (Access-group first-generation cephalosporin). A meta-analysis, published in 2022, including eleven studies comparing cefazolin with cefuroxime, concluded that cefazolin is as effective as cefuroxime for prophylaxis [20]. Other studies compared ceftriaxone with cefazolin, showing that cefazolin could be a safe and effective option even as empiric treatment (ex. for urinary tract infections [21,22]). It is also effective for patients allergic to penicillins [23].

Cefazolin is active in Gram-positive bacteria, including staphylococci, which represents a considerable proportion of positive samples in our study (overall 2020–2024, approximately 24%), with a high percentage of *Staphylococcus aureus*. Thus, it is a complementary argument for its inclusion in our internal protocols as a useful treatment option, considering the side effects of anti-staphylococcal penicillins [24,25].

Concerning the other most used cephalosporin, ceftriaxone showed a high consumption (notably in the last two years, 2023–2024) with a notable positive AAPC of 31.10. This value indicates a very accelerated and sustained increase in ceftriaxone on medium-term consumption. From the data reported for parenteral antibiotics in GLASS (for Romania), analyzed in the same period as those in our study (2020–2024), ceftriaxone was the most used (mainly in the COVID-19 pandemic period, between 2020 and 2021, followed by a slow decrease between 2022 and 2024) [26].

*Escherichia coli*, *Klebsiella pneumoniae*, *Proteus mirabilis*, *Enterobacter cloacae*, *Citrobacter freundii*, *Citrobacter koseri*, and *Providencia stuartii* were among the most important enterobacteria found in our analysis. For these pathogens in critically ill patients, ceftriaxone and meropenem have remained important therapeutic options over time [27,28], which was mirrored in the consumption results.

In this study, ESBL strains were noticed (*Klebsiella* spp. > *Escherichia coli* > *Enterobacter* spp.), for which cephalosporins are ineffective [29]. The frequent identification of ESBL-producing bacteria implies the establishment of therapeutic alternatives. This does not automatically justify the use of carbapenems, but these antibiotics may be a valuable alternative. From our study, meropenem was the most used carbapenem and registered a positive AAPC > 10% (overall 2020–2024), but there were no major changes in consumption in the last three years. A six-year study also reported meropenem as the most commonly used carbapenem [30]. Third-generation cephalosporin-resistant and carbapenem-resistant *Enterobacterales* are included in the “critical group” by the WHO-BPPL (World Health Organization—Bacterial Priority Pathogen List 2024) [11].

Regarding carbapenem-resistance, strains of *Pseudomonas* spp. have been identified, notably in 2022 and 2023. WHO-BPPL classifies *Pseudomonas aeruginosa* carbapenem-resistant in the “High group” [11]. In our study, a total of 45 isolates (13.47%) of CR from the total positive strains of *Pseudomonas* spp. during all five years was noticed. A study reported the same number (45 isolates) and a superior percentage of 26.9% but only for half a year in a hospital in Egypt [31]. Other sources reported higher values (37.2%, for a hospital from Saudi Arabia) [32]. A systematic review, published in 2024, estimates a high percentage of carbapenem-resistant *Pseudomonas aeruginosa* (CRPA) in Romania of 46.4% [33]. Although meropenem consumption was high, the proportion of CRPA was low in comparison with the other reported values, indicating that selection pressure did not increase the resistance during the analyzed period.

Although *Acinetobacter baumannii* strains were identified (mostly in respiratory specimens), there were only 54 isolates over five years, with the highest numbers during the 2020–2021 COVID-19 pandemic. A substantial proportion of these (approximately 30%) were MDR. Carbapenem-resistant *A. baumannii* (CRAB) is considered a subcategory of MDR because this resistance is often associated with concurrent resistance to most other classes of antibiotics [34]. CRAB is classified by the WHO-BPPL as a “Critical group” and is a first-class priority [11].

Vancomycin (AAPC 2020–2024 of 25.55) and linezolid (AAPC 2020–2024 of 5.06) were used in large quantities, contributing to the overall percentage of the Watch group (vancomycin) and Reserve group (linezolid). A notable percentage of MRSA strains (approximately 22% of the total positive samples for *Staphylococcus aureus* in our analysis) contributes to promoting the consumption of these antibiotics. Other studies reported a general percentage of 25% in *Staphylococcus aureus* isolates being MRSA [35], similar to our result. International clinical guidelines recommend both vancomycin and linezolid as therapeutic options for MRSA infections, with vancomycin being frequently used in severe cases and linezolid as an effective alternative, including for the transition to oral administration or in situations of vancomycin intolerance [36]. *Staphylococcus aureus* is part of the ESKAPE group, and in our analysis, its prevalence is noted in respiratory, skin and soft tissue and blood samples. The MRSA type is well-known for its high risk of causing serious infections, mainly bacteremia, pneumonia and soft tissue infections. According to WHO-BPPL, it is classified in the “High group” category [11].

Frequent and prolonged use of vancomycin increases the risk of developing resistant strains, not only for MRSA (change in VRSA) but for other bacteria, most commonly of the VRE type, such as enterococci. There are several studies that analyze the oral administration of vancomycin as a risk of VRE but even parenteral administration can determine VRE strains [37,38]. Although vancomycin is a frequently used antibiotic in our hospital, we found only 4.82% of VRE species from the total enterococci positive samples (overall 2020–2024). There is a large variation between studies’ estimates of VRE’s proportions among *Enterococcus* spp., but a meta-analysis estimated a pooled, hospital-wide VRE of 7.3% for the European Region (estimation was made for a ten-year period, between 2010 and 2020) [39]. Thus, a percentage lower than 5% for our hospital suggested judicious vancomycin use. WHO-BPPL mentions *Enterococcus faecium* vancomycin-resistant in the “High group” category [11].

Most of the Reserve group antibiotics were used in specific cases and were either introduced or withdrawn from use at certain times, thus clinically considerable AAPCs cannot be assessed except for tigecycline, linezolid and colistin. Globally, a high colistin consumption is correlated with an increase in resistant bacteria [40,41] (for example, *Acinetobacter* colistin-resistant [42]). Our results revealed a decrease in colistin consumption in the last year and a negative AAPC for the analyzed period, which is in agreement with its consideration as an antibiotic of last resort.

Previous studies reported the following microorganisms: *Escherichia coli*, *Staphylococcus aureus*, *Enterococcus* spp., *Klebsiella* spp., *Candida* spp., *Clostridioides difficile*, *Enterobacter* spp., *Proteus* spp. and *Pseudomonas aeruginosa* as those most commonly responsible for healthcare-associated infections across Europe. In Romania, the epidemiological profile was dominated by *Clostridioides difficile*, *Acinetobacter baumannii*, *Klebsiella pneumoniae*, *Pseudomonas aeruginosa* and *Staphylococcus aureus* [43]. In our study, the results revealed the same frequently identified bacteria (excepting *C. difficile* and *Candida* spp.). Future perspectives of the current study will consider the analysis of common complications of antibiotic treatments, including fungal and *C. difficile* infections [44,45].

Even though in our study the ESKAPE bacteria colonized predominantly specimens from the respiratory and digestive systems as well as from skin and soft tissue, they were also found in blood specimens, which warns about the risk of serious systemic infections. Previous studies reported peaks in the isolation of ESKAPE bacteria across all types of samples in 2021 [46], but an important increase in positive blood cultures was observed for *Klebsiella pneumoniae*, *Acinetobacter baumannii*, *Staphylococcus aureus* and *Pseudomonas aeruginosa* over a seven-year period [47]. Further research will consider developing the analysis in this direction in correlation with specific antibiotic treatments.

Hospital antibiotic use and microbiological data were interpreted using WHO-recommended resources, such as DDD, AWaRe and BPPL. The reduced number of resistant bacteria over five years, with relatively stable trends in bacterial isolates, without marked increases, suggests effective antimicrobial stewardship practices [48]. The antibiotics used were consistent with the epidemiological profile. The de-escalation from cefuroxime to cefazolin in our protocols led to an important increase in the percentage of Access group antibiotics. A meta-analysis mentioned that well-implemented antimicrobial stewardship practices were linked to a lower consumption of antibiotics from the WHO Watch group, effectively preserving these critical medicines [49].

The retrospective approach of the study provided only background information and did not capture actual epidemiological trends. It is based on data belonging to a single hospital, which are limited to local cases, local resistance profiles and local prescribing protocols, hindering generalizability to other healthcare units (where the epidemiological profile does not allow de-escalation of prophylaxis). Despite these limitations, the study offers real-world evidence on antibiotic consumption and microbiological trends over a five-year period.

At the national level, a goal of the WHO is that more than 60% of total antibiotic consumption belongs to the Access group (as a target indicator in antibiotic consumption). More recently, in 2024, a target of over 70% Access antibiotics has been recommended globally [50,51]. Even though these percentages are not ideal, our results show a favorable evolution in this regard. Future efforts will focus on enhanced monitoring of antibiotic use (by regular audits), mentoring sessions, refining internal protocols to include more Access antibiotics, and encouraging de-escalation.

## 4. Materials and Methods

### 4.1. Study Design

This study is based on a retrospective design, analyzing the antibiotic consumption and laboratory culture results over a period of five years (2020–2024) in a multidisciplinary hospital in Romania. The data belongs to two hospitals’ departments: Closed Circuit Pharmacy and the Microbiology Department and was approved by the Health Care Ethical Committee of Sanador Clinical Hospital, document number 14,119 (8 December 2025). Inclusion criteria: 1. systemic antibiotics (oral and parenteral); 2. positive samples; 3. specimens from inpatients (who were admitted to the hospital for more than 24 h). Exclusion criteria: 1. topical antibiotics; 2. antibiotics in pediatric pharmaceutical formulations (oral solutions, oral suspensions, syrups); 3. antifungals; 4. antivirals; 5. negative samples (for which no pathogen was identified); 6. antibiotic consumption associated pathogens (as fungi and *C. difficile*); 7. duplicates (same etiologic agent in the same sample on an interval of 72 h); 8. samples from outpatients.

### 4.2. Antibiotic Consumption, Classification and DDDs

Antibiotic consumption was extracted from the pharmacy’s internal management system reports (PAx CentreMed v.7.8, Softeh, București, Romania) as quantities in therapeutic units (ex. ampoules, vials, tablets). The analysis included only the antibiotics for systemic use (oral and parenteral). This approach allows clinical relevance as these antibiotics are comparable with international data (most guidelines focus on systemic antibiotic therapy [52,53]).

The DDD and the AWaRe classifications are two complementary tools used to monitor and compare antibiotic use. The DDD data and AWaRe categories help identify trends in antibiotic use, detect potential overuse of high-risk antibiotics and support strategies for responsible prescribing to limit antimicrobial resistance. Therapeutic units were converted to DDD using the WHO ATC/DDD index [54,55]. In our study, the results were expressed as DDD per 100 bed days, which is feasible for hospital analysis. According to the WHO-ATC/DDD guidelines, a bed day is considered a day during which the patient is confined to a bed and stays overnight [56].

Antibiotics were grouped in the AWaRe classification. The WHO updates it every two years; the latest version (from 2025) was applied in our study. We also grouped the antibiotics by pharmacologic subcategories (ATC).

Drug utilization data presented as DDDs give a rough estimate of consumption, not an exact actual drug use [56], which only allows for standardization and comparisons at the international level.

The WHO periodically updates DDD values based on new clinical data. In order to make adequate comparisons (at the same reference values), the calculation of DDD in the current study was performed for all years using current values (ATC/DDD index 2025). The five-year interval was chosen to allow the identification of usage trends and not just year-to-year fluctuations as would have been the case with a shorter period. Although a period longer than five years would allow for a more detailed analysis, changes in protocols and product availability on the market could have determined inconsistencies between the analyzed data.

### 4.3. Average Annual Percent Change (AAPC) Determination

Average Annual Percent Change is a statistical indicator used to summarize the rate of change during a period, usually applied for diseases (ex. cancer) [57,58] but it was successfully applied for medicine consumption (ex. antibiotics) [59]. The AAPC and 95% Confidence Interval were calculated for each antibiotic and for total consumption of the three general categories (Access, Watch, Reserve) by Joinpoint Regression Software (v.5.4.0), Statistical Research and Applications Branch, Calverton, United States of America [58]. In the AAPC calculation, to overcome the limitation of dividing by zero for antibiotics for which no consumption was recorded, the zero value of DDD/100 days was replaced with the minimum threshold of 0.01. This technical adjustment was needed to allow the continuity of calculations [60].

### 4.4. Microbiological Data Processing

Microbiology Laboratory results were extracted as monthly reports (during 2020–2024) from internal management software (MedLab Medis 4 v.5.4.21., Softeh, București Romania). The data included: type of analyzed specimen, identified pathogen/pathogens and resistance specifications (MDR, ESBL, CR, VRE, MRSA).

The analysis was conducted only for positive samples; no reports were made on the total number of samples tested. A positive sample is considered the specimen for which at least one infectious bacterial agent has been identified. We chose to report only on positives, without the large number of negative samples overshadowing the results. Moreover, except for prophylactic use and empirical initiation for severe cases, antibiotics should be prescribed when microbiological results indicate an infectious pathology.

The relative frequency of the pathogen in positive samples was calculated as a percentage, reporting the number of samples where the pathogen was identified to the total number of positive samples. The results were shown on genera, highlighting the bacteria with the greatest clinical importance. For resistant strains, the percentage was calculated by reporting the number of resistant isolates to the total isolates of the same bacteria.

The samples were grouped into six categories: digestive tract specimens, genitourinary tract specimens, respiratory tract specimens, blood specimens, skin and soft tissue specimens and other specimens. ESKAPE pathogens are a group of six bacteria: *Enterococcus faecium*, *Staphylococcus aureus*, *Klebsiella pneumoniae*, *Acinetobacter baumannii*, *Pseudomonas aeruginosa*, and *Enterobacter* spp., known for their increased levels of resistance. These microorganisms are very common in the hospital environment and are involved in healthcare-associated infections, leading to prolonged hospitalization and the need for complex antibiotic therapy [61,62]. Using these two groupings, the distribution of the sample types was performed on ESKAPE pathogens. Percentages were calculated by relating the number of positive samples of the bacteria from a certain category to the total number of positive samples of the bacteria.

## 5. Conclusions

In the current study, antibiotic consumption analysis for the five-year period showed that Watch antibiotics were predominantly used in the first years (especially cefuroxime, ceftriaxone, meropenem, and ertapenem). After cefazolin was introduced in protocols (2023), the use of Access antibiotics increased remarkably from 10.15% in 2020 to 41.02% in 2024, leading to an AAPC for the five-year period of approximately 50%. For Watch antibiotics, a negative AAPC of approximately −6% was recorded. Reserve antibiotics were used less, the most important being linezolid, tigecycline (slight upward trend) and colistin (noteworthy decrease). The relative frequency of pathogens in positive samples revealed that *Staphylococcus aureus* was the most frequent (with a high percentage of MRSA), closely followed by *Escherichia coli*, then *Klebsiella* species (especially *Klebsiella pneumoniae*), for which an important number of ESBL-producing species were observed (*Klebsiella* spp. > *E. coli*). ESKAPE bacteria mainly colonized samples from the respiratory tract, digestive tract, and skin and soft tissues. Even if resistant strains have been identified among pathogens, no concerning trends have been noticed over the five-year period. Thus, the antibiotic consumption was in accordance with the epidemiological profile, suggesting appropriate antimicrobial management. Further studies will aim to analyze antibiotic consumption on department-specific and also in-depth analysis of ESKAPE bacteria in blood specimens.

## Figures and Tables

**Table 1:** Access antibiotic consumption during analyzed period, 2020–2024.

Class	ATC Code	Antibiotic Substance (Administration Route)	DDD/100 Bed Days					AAPC (95% CI)
			2020	2021	2022	2023	2024	
aminoglycosides	J01GB06	amikacin	1.69	1.64	1.80	1.27	1.20	−8.98 (−16.28;−1.42)
aminoglycosides	J01GB03	gentamicin	0.38	0.37	0.41	0.31	0.25	−10.00 (−20.11;0.86)
cephalosporins G1	J01DB04	cefazolin	0.01 *	0.01 *	0.01 *	10.41	24.98	3712.95 (17.99;112,239.48)
imidazolidines	J01XD01	metronidazole (o)	0.09	0.08	0.04	0.02	0.03	−30.17 (−49.37;−4.22)
imidazolidines	J01XD01	metronidazole (p)	1.40	1.70	1.53	1.12	1.10	−8.64 (−26.58;13.65)
lincosamides	J01FF01	clindamycin (o)	0.01	0.02	0.01	0.01 *	0.01	−0.88 (−32.99;40.84)
lincosamides	J01FF01	clindamycin (p)	0.01	0.01	0.03	0.02	0.02	153.64 (−27.88;748.60)
penicillins	J01CA04	amoxicillin	0.07	0.06	0.02	0.01	0.08	−19.15 (−69.24;105.66)
penicillins	J01CR02	amoxicillin/clavulanic acid (p)	0.90	0.94	0.79	0.84	0.94	−0.14 (−8.16;8.36)
penicillins	J01CR02	amoxicillin/clavulanic acid (o)	0.25	0.14	0.14	0.22	0.14	−7.69 (−35.37;30.66)
penicillins	J01CA01	ampicillin (p)	0.51	0.39	0.27	0.42	0.40	−3.99 (−18.42;12.65)
penicillins	J01CF04	oxacillin	0.63	0.06	0.21	0.18	0.38	0.15 (−61.16;153.04)
sulfonamides	J01EE01	sulfamethoxazole/trimethoprim (o)	0.17	0.24	0.24	0.08	0.20	−7.22 (−34.32;30.72)
tetracyclines	J01AA02	doxycycline	0.15	0.44	0.22	0.16	0.53	15.74 (−51.64;170.62)
Total Access			6.26	6.08	5.72	15.06	30.24	50.05 (−7.72;141.07)

ATC = Anatomical Therapeutic Chemical classification system; DDD = Defined Daily Dose; AAPC = Average Annual Percent Change; CI = Confidence Interval; * = 0.01 value used for AAPC calculation because the DDD’s real value was zero.

**Table 2:** Watch antibiotic consumption during analyzed period, 2020–2024.

Class	ATC Code	Antibiotic Substance (Administration Route)	DDD/100 Bed Days					AAPC (95% CI)
			2020	2021	2022	2023	2024	
carbapenems	J01DH03	ertapenem	5.11	5.46	4.22	3.47	3.64	−10.70 (−22.57;2.77)
carbapenems	J01DH51	imipenem/cilastatin	0.25	0.10	0.12	0.04	0.07	−29.07 (−45.98;−7.64)
carbapenems	J01DH02	meropenem	4.47	5.15	6.37	6.74	6.91	12.08 (3.71;20.74)
cephalosporins G2	J01DC02	cefuroxime (o)	1.92	1.71	1.68	1.41	1.24	−10.10 (−12.63;−7.65)
cephalosporins G2	J01DC02	cefuroxime (p)	27.85	31.35	35.50	21.05	0.06	−71.49 (−97.24;178.50)
cephalosporins G3	J01DD02	ceftazidime	0.46	0.41	0.40	0.17	0.08	−35.36 (−61.26;5.93)
cephalosporins G3	J01DD04	ceftriaxone	6.74	6.18	6.44	11.09	19.48	31.10 (−20.49;114.35)
fluoroquinolones	J01MA02	ciprofloxacin (o)	0.20	0.16	0.19	0.15	0.14	−6.57 (−13.00;0.01)
fluoroquinolones	J01MA02	ciprofloxacin (p)	0.05	0.07	0.06	0.05	0.03	−9.99 (−29.80;14.55)
fluoroquinolones	J01MA12	levofloxacin (o)	0.14	0.18	0.50	0.27	0.14	3.45 (−49.51;93.99)
fluoroquinolones	J01MA12	levofloxacin (p)	1.23	1.36	1.55	2.29	2.09	17.03 (6.31;28.23)
fluoroquinolones	J01MA14	moxifloxacin (o)	0.01	0.05	0.08	0.10	0.05	43.16 (−8.77;122.70)
fluoroquinolones	J01MA14	moxifloxacin (p)	0.15	0.11	0.11	0.63	0.25	32.43 (−31.35;150.26)
glycopeptides	J01XA02	teicoplanin	0.45	0.37	0.42	0.13	0.21	−22.84 (−45.45;7.95)
glycopeptides	J01XA01	vancomycin	1.37	1.99	2.60	2.78	3.61	25.55 (11.70;40.64)
macrolides	J01FA10	azithromycin (o)	0.06	0.10	0.38	0.28	0.15	34.85 (−39.81;193.64)
macrolides	J01FA09	clarithromycin (o)	0.22	0.23	0.07	0.07	0.10	−24.65 (−53.13;19.66)
macrolides	J01FA09	clarithromycin (p)	0.37	0.15	0.11	0.08	0.12	−24.89 (−40.14;−4.94)
penicillins	J01CR05	piperacillin/tazobactam	1.23	1.27	1.66	1.65	1.12	0.73 (−24.55;33.95)
phosphonics	J01XX01	fosfomycin (o)	0.03	0.02	0.03	0.01	0.03	4.34 (−8.62;18.32)
Total Watch			52.31	56.43	62.46	52.46	39.54	−6.13 (−26.00;18.89)

ATC = Anatomical Therapeutic Chemical classification system; DDD = Defined Daily Dose; AAPC = Average Annual Percent Change; CI = Confidence Interval.

**Table 3:** Reserve antibiotics consumption during analyzed period, 2020–2024.

Class	ATC Code	Antibiotic Substance (Administration Route)	DDD/100 Bed Days					AAPC (95% CI)
			2020	2021	2022	2023	2024	
monobactams	J01DF01	aztreonam	0.01 *	0.01 *	0.01 *	0.01 *	0.06	255.02 (−58.49;2617.07)
cephalosporins G5	J01DI54	ceftolozane/tazobactam	0.01	0.01 *	0.01 *	0.12	0.07	96.00 (−3.52;325.68)
cephalosporins G3	J01DD52	ceftazidime/avibactam	0.02	0.04	0.02	0.05	0.26	63.13 (−7.50;181.07)
cephalosporins G5	J01DI02	ceftaroline	0.01 *	0.01 *	0.01 *	0.01 *	0.03	207.96 (−54.19;1775.60)
polymyxins	J01XB01	colistin	0.36	0.34	0.68	0.46	0.30	−1.14 (−29.40;37.18)
phosphonics	J01XX01	fosfomycin (p)	0.01 *	0.01 *	0.01	0.08	0.04	549.59 (15.47;3416.68)
oxazolidinones	J01XX08	linezolid (p)	2.17	2.03	3.06	2.66	2.43	5.06 (−10.25;22.13)
tetracyclines	J01AA12	tigecycline	0.56	0.60	0.86	0.91	0.76	10.65 (−9.34;34.31)
Total Reserve			3.12	3.02	4.62	4.28	3.93	8.44 (−8.58;27.78)

ATC = Anatomical Therapeutic Chemical classification system; DDD = Defined Daily Dose; AAPC = Average Annual Percent Change; CI = Confidence Interval; * = 0.01 value used for AAPC calculation because the DDD’s real value was zero.

**Table 4:** Relative frequency of the pathogens identified in positive samples over the 2020–2024 period.

Bacterial Species	Number of Isolates (Relative Frequency Among Positive Samples, %)					
	2020	2021	2022	2023	2024	Overall 2020–2024
*Staphylococcus* spp.	216 (24.94)	201 (21.00)	175 (23.21)	238 (24.49)	153 (21.70)	983 (23.11)
*Staphylococcus aureus*	201 (23.21)	178 (18.60)	160 (21.22)	211(21.71)	124 (17.59)	874 (20.55)
Other staphylococci	15 (1.73)	23 (2.40)	15 (1.99)	27 (2.7)	29 (4.11)	109 (2.56)
*Escherichia coli*	173 (19.98)	178 (18.60)	159 (21.09)	186 (19.14)	177 (25.11)	873 (20.52)
*Klebsiella* spp.	174 (20.09)	170 (17.76)	150 (19.89)	184 (18.93)	131 (18.58)	809 (19.02)
*Klebsiella pneumoniae*	65 (7.51)	37 (3.87)	83 (11.01)	177 (18.21)	103 (14.61)	465 (10.93)
Other species	109 (12.59)	133 (13.90)	67 (8.89)	7 (0.72)	28 (3.97)	344 (8.09)
*Enterococcus* spp.	111 (12.82)	150 (15.67)	97 (12.86)	90 (9.26)	71 (10.07)	519 (12.20)
*Enterococcus faecalis*	5 (0.58)	50 (5.22)	71 (9.42)	62 (6.38)	50 (7.09)	238 (5.59)
*Enterococcus faecium*	12 (1.39)	32 (3.34)	20 (2.65)	24 (2.47)	18 (2.55)	106 (2.49)
Other enterococci	94 (10.85)	68 (7.11)	6 (0.80)	4 (0.41)	3 (0.43)	175 (4.11)
*Pseudomonas* spp.	61 (7.05)	69 (7.21)	53 (7.03)	102 (10.49)	49 (6.95)	334 (7.85)
*Pseudomonas aeruginosa*	56 (6.47)	68 (7.11)	51 (6.76)	101 (10.39)	47 (6.67)	323 (7.59)
Other species	5 (0.58)	1 (0.10)	2 (0.27)	1 (0.10)	2 (0.28)	11 (0.26)
*Proteus* spp.	29 (3.35)	53 (5.54)	55 (7.29)	74 (7.61)	47 (6.67)	258 (6.06)
*Proteus mirabilis*	4 (0.46)	1 (0.10)	12 (1.59)	70 (7.20)	43 (6.10)	130 (3.06)
Other species	25 (2.89)	52 (5.43)	43 (5.70)	4 (0.41)	4 (0.57)	128 (3.01)
*Streptococcus* spp.	32 (3.70)	28 (2.93)	19 (2.52)	32 (3.29)	7 (0.99)	118 (2.77)
A-group streptococci	8 (0.92)	3 (0.31)	1 (0.13)	10 (1.03)	1 (0.14)	23 (0.54)
B-group streptococci	12 (1.39)	14 (1.46)	6 (0.80)	7 (0.72)	0 (0.00)	39 (0.92)
C,D,G-groups streptococci	6 (0.69)	2 (0.21)	5 (0.66)	5 (0.51)	5 (0.71)	23 (0.54)
Other streptococci	6 (0.69)	9 (0.94)	7 (0.93)	10 (1.03)	1 (0.14)	33 (0.78)
*Stenotrophomonas maltophilia*	8 (0.92)	30 (3.13)	16 (2.12)	13 (1.34)	15 (2.13)	82 (1.93)
*Enterobacter* spp.	12 (1.39)	28 (2.93)	10 (1.33)	18 (1.85)	12 (1.70)	80 (1.88)
*Enterobacter cloacae*	7 (0.81)	11 (1.15)	9 (1.19)	11 (1.13)	5 (0.71)	43 (1.01)
Other species	5 (0.58)	17 (1.78)	1 (0.13)	7 (0.72)	7 (0.99)	37 (0.87)
*Acinetobacter* spp.	18 (2.08)	21 (2.19)	7 (0.93)	7 (0.72)	8 (1.13)	61 (1.43)
*Acinetobacter baumannii*	15 (1.73)	19 (1.99)	7 (0.93)	7 (0.72)	6 (0.85)	54 (1.27)
Other species	3 (0.35)	2 (0.21)	0 (0.00)	0 (0.00)	2 (0.28)	7 (0.16)
*Morganella* spp.	7 (0.81)	10 (1.04)	4 (0.53)	4 (0.41)	7 (0.99)	32 (0.75)
*Citrobacter* spp.	7 (0.81)	6 (0.63)	1 (0.13)	2 (0.21)	11 (1.56)	27 (0.63)
*Citrobacter freundii*	1 (0.12)	1 (0.10)	1 (0.13)	1 (0.10)	5 (0.71)	9 (0.21)
*Citrobacter koseri*	2 (0.23)	4 (0.42)	0 (0.00)	0 (0.00)	4 (0.57)	10 (0.24)
Other species	4 (0.46)	1 (0.10)	0 (0.00)	1 (0.10)	2 (0.28)	8 (0.19)
*Providencia* spp.	2 (0.23)	4 (0.42)	1 (0.13)	3 (0.31)	2 (0.28)	12 (0.28)
*Providencia stuartii*	1 (0.12)	0 (0.00)	1 (0.13)	2 (0.21)	1 (0.14)	5 (0.12)
Other species	1 (0.11)	4 (0.42)	0 (0.00)	1 (0.10)	1 (0.14)	7 (0.16)
*Moraxella* spp.	3 (0.35)	0 (0.00)	0 (0.00)	1 (0.10)	1 (0.14)	5 (0.12)
Other pathogens	13 (1.50)	9 (0.94)	7 (0.93)	18 (1.85)	14 (1.99)	61 (1.43)
other staphylococci: *S. epidermidis*, *S. warneri*, *S. haemolyticus*, *S. capitis urealyticus*, *S. saprophyticus*, *S. lugdunensis*; other *Klebsiella*: *K. oxytoca*, *K. ozaenae*, *K. aerogenes*, *K. variicola*; other enterococci: *E. gallinarum*, *E. avium*, *E. casseliflavus*, *E. raffinosus*; other *Pseudomonas*: *P. fluorescens*, *P. mendocina*, *P. stutzeri*; other *Proteus*: *P. vulgaris*, *P. hauseri*, *P. penneri*; other streptococci: *S. viridans*, *S. mutans*, *S. intermedius*, *S. bovis*, *S. anginosus*; *S. parasanguinis*, *S. constellatus*, *S. pneumoniae*; other *Enterobacter*: *E. hormaechei*, *E. kobei*, *E. bugandensis*; other *Acinetobacter*: *A. lwoffli*, *A. haemolyticus*, *A. usingii*; other *Citrobacter*: *C. youngae*, *C. brakii*, *C. amalonaticus*; other *Providencia*: *P. retgerii*, *P. rustigianii*; other pathogens: *Serratia marcescens*, *Burkholderia* spp. *(cepacia*, *gladioli)*, *Bacteroides* spp. *(fragilis*, *vulgatus)*, *Haemophilus* spp. *(influenzae*, *parainfluenzae)*, *Prevotella bivia*, *Salmonella enterica*, *Micrococcus luteus*, *Gardnerella vaginalis*, *Corynebacterium striatum*.						

**Table 5:** Distribution of strains with major antimicrobial resistance characteristics.

Bacterial Species	Number of Isolates (Relative Frequency of Resistant Isolates, %)					
	2020	2021	2022	2023	2024	Overall 2020–2024
*Acinetobacter baumannii*	15	19	7	7	6	54
MDR	5 (33.33)	0 (0.00)	4 (57.14)	6 (85.71)	1 (16.67)	16 (29.63)
*Enterobacter* spp.	12	28	10	18	12	80
ESBL	1 (8.33)	8 (28.57)	2 (20.00)	3 (16.67)	2 (16.67)	16 (20.00)
*Enterococcus* spp.	111	150	97	90	71	519
VRE	3 (2.70)	2 (1.33)	5 (5.15)	5 (5.56)	10 (14.08)	25 (4.82)
*Escherichia coli*	173	178	159	186	177	873
ESBL	22 (12.72)	32 (17.98)	33 (20.75)	40 (21.51)	34 (19.21)	161 (18.44)
*Klebsiella* spp.	174	170	150	184	131	809
ESBL	96 (55.17)	55 (32.35)	73 (48.67)	81 (44.02)	45 (34.35)	350 (43.26)
*Pseudomonas* spp.	61	69	53	102	49	334
CR	3 (4.92)	6 (8.70)	12 (22.64)	17 (16.67)	7 (14.29)	45 (13.47)
*Staphylococcus aureus*	201	178	160	211	124	874
MRSA	47 (23.38)	43 (24.16)	39 (24.38)	61 (28.91)	35 (28.23)	225 (25.74)

MDR = Multidrug-Resistant; ESBL = Extended-Spectrum Beta-Lactamase; CR—Carbapenem-Resistant; VRE—Vancomycin-Resistant *Enterococcus*; MRSA = Methicillin-Resistant *Staphylococcus aureus*.

## Data Availability

Data are contained within the article and Supplementary Materials.

## Supplementary Materials

The following supporting information can be downloaded at: https://www.mdpi.com/article/10.3390/antibiotics15030288/s1, Figure S1: AWaRe classification system integrated in hospital’s antibiotic management; Figure S2: Amikacin 2020–2024 consumption trend; Figure S3: Gentamicin 2020–2024 consumption trend; Figure S4: Cefazolin 2020–2024 consumption trend; Figure S5: Metronidazole (oral) 2020–2024 consumption trend; Figure S6: Metronidazole (parenteral) 2020–2024 consumption trend; Figure S7: Clindamycin (oral) 2020–2024 consumption trend; Figure S8: Clindamycin (parenteral) 2020–2024 consumption trend; Figure S9: Amoxicillin 2020–2024 consumption trend; Figure S10: Amoxicillin/clavulanic acid (parenteral) 2020–2024 consumption trend; Figure S11: Amoxicillin/clavulanic acid (oral) 2020–2024 consumption trend; Figure S12: Ampicillin 2020–2024 consumption trend; Figure S13: Oxacillin 2020–2024 consumption trend; Figure S14: Sulfamethoxazole/trimethoprim (oral) 2020–2024 consumption trend; Figure S15: Doxicycline 2020–2024 consumption trend; Figure S16: Access group antibiotics 2020–2024 consumption trend; Figure S17: Ertapenem 2020–2024 consumption trend; Figure S18: Imipenem/cilastatin 2020–2024 consumption trend; Figure S19: Meropenem 2020–2024 consumption trend; Figure S20: Cefuroxime (oral) 2020–2024 consumption trend; Figure S21: Cefuroxime (parenteral) 2020–2024 consumption trend; Figure S22: Ceftazidime 2020–2024 consumption trend; Figure S23: Ceftriaxone 2020–2024 consumption trend; Figure S24: Ciprofloxacin (oral) 2020–2024 consumption trend; Figure S25: Ciprofloxacin (parenteral) 2020–2024 consumption trend; Figure S26: Levofloxacin (oral) 2020–2024 consumption trend; Figure S27: Levofloxacin (parenteral) 2020–2024 consumption trend; Figure S28: Moxifloxacin (oral) 2020–2024 consumption trend; Figure S29: Moxifloxacin (parenteral) 2020–2024 consumption trend; Figure S30: Teicoplanin 2020–2024 consumption trend; Figure S31: Vancomycin 2020–2024 consumption trend; Figure S32: Azithromycin (oral) 2020–2024 consumption trend; Figure S33: Clarithromycin (oral) 2020–2024 consumption trend; Figure S34: Clarithromycin (parenteral) 2020–2024 consumption trend; Figure S35: Piperacillin/tazobactam 2020–2024 consumption trend; Figure S36: Fosfomycin (oral) 2020–2024 consumption trend; Figure S37: Watch group 2020–2024 consumption trend; Figure S38: Aztreonam 2020–2024 consumption trend; Figure S39: Ceftolozane/tazobactam 2020–2024 consumption trend; Figure S40: Ceftazidime/avibactam 2020–2024 consumption trend; Figure S41: Ceftaroline 2020–2024 consumption trend; Figure S42: Colistin 2020–2024 consumption trend; Figure S43: Fosfomycin (parenteral) 2020–2024 consumption trend; Figure S44: Linezolid (parenteral) 2020–2024 consumption trend; Figure S45: Tigecycline 2020–2024 consumption trend; Figure S46: Reserve group 2020–2024 consumption trend; Figure S47: Distribution of positive samples for each ESKAPE pathogen in 2020; Figure S48: Distribution of positive samples for each ESKAPE pathogen in 2021; Figure S49: Distribution of positive samples for each ESKAPE pathogen in 2022; Figure S50: Distribution of positive samples for each ESKAPE pathogen in 2023; Figure S51: Distribution of positive samples for each ESKAPE pathogen in 2024.

## Abbreviations

The following abbreviations are used in this manuscript:

AAPC	Annual Average Percentage Change
AMR	Antimicrobial resistance
ATC	Anatomical Therapeutic Chemical classification system
AWaRe	Access Watch Reserve antibiotics classification
BPPL	Bacterial Priority Pathogen List
COVID-19	Coronavirus Disease 2019
CR	Carbapenem-Resistant
CRAB	Carbapenem-Resistant *Acinetobacter baumannii*
CRPA	Carbapenem-Resistant *Pseudomonas aeruginosa*
DDD	Defined Daily Dose
ESBL	Extended-Spectrum Beta-Lactamase
ESKAPE	Bacteria group: *Enterococcus faecium*, *Staphylococcus aureus*, *Klebsiella pneumoniae*, *Acinetobacter baumannii*, *Pseudomonas aeruginosa* and *Enterobacter* spp.
GLASS	Global Antimicrobial Resistance and Use Surveillance System
MDR	Multidrug-Resistant
MRSA	Methicillin-Resistant *Staphylococcus aureus*
VRE	Vancomycin-Resistant *Enterococcus*
WHO	World Health Organization
