# Supplementary material for: Analysis of Antibiotic Consumption Trends and Pathogens’ Epidemiological Profile Within a Multidisciplinary Clinical Hospital from Romania
Source: Antibiotics (Basel). 2026 Mar 12;15(3):288. doi: 10.3390/antibiotics15030288 (PMC13024390; doi:10.3390/antibiotics15030288)
Supplement: Supplementary file 1 [file antibiotics-15-00288-s001.zip › antibiotics-4143201-supplementary.pdf]

Article

Analysis of Antibiotic Consumption Trends and Pathogens' Epidemiological Profile Within a Multidisciplinary Clinical Hospital from Romania

Supplementary Materials

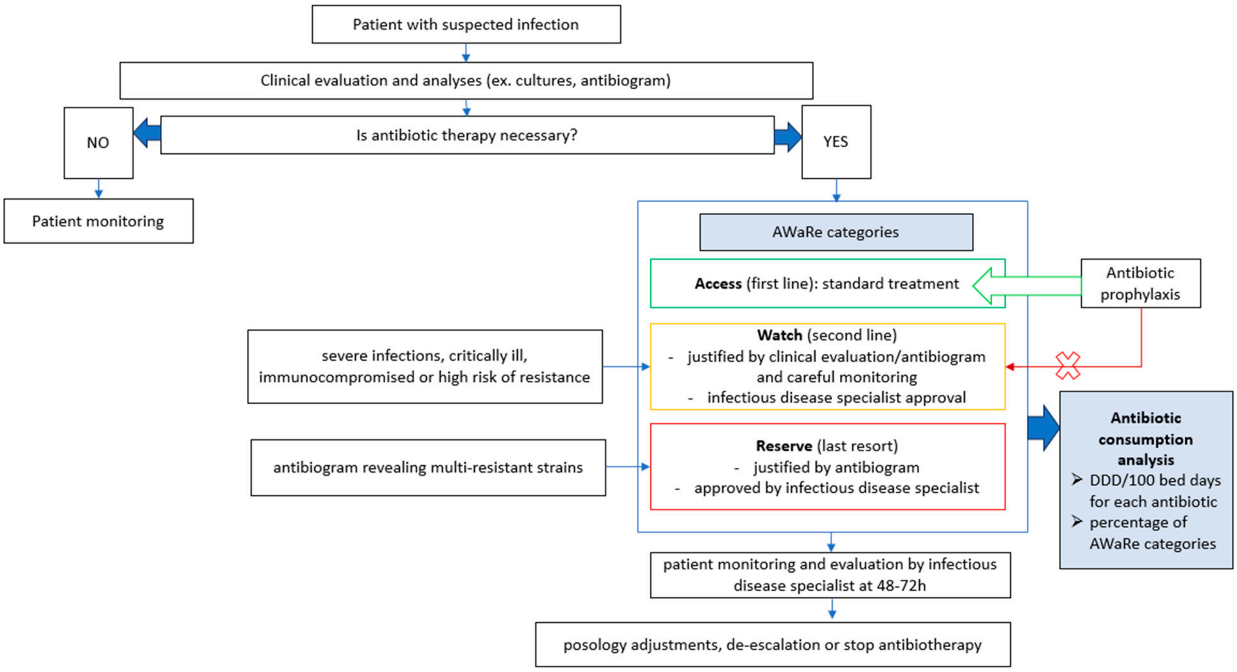

Figure S1. AWaRe classification system integrated in hospital's antibiotic management.

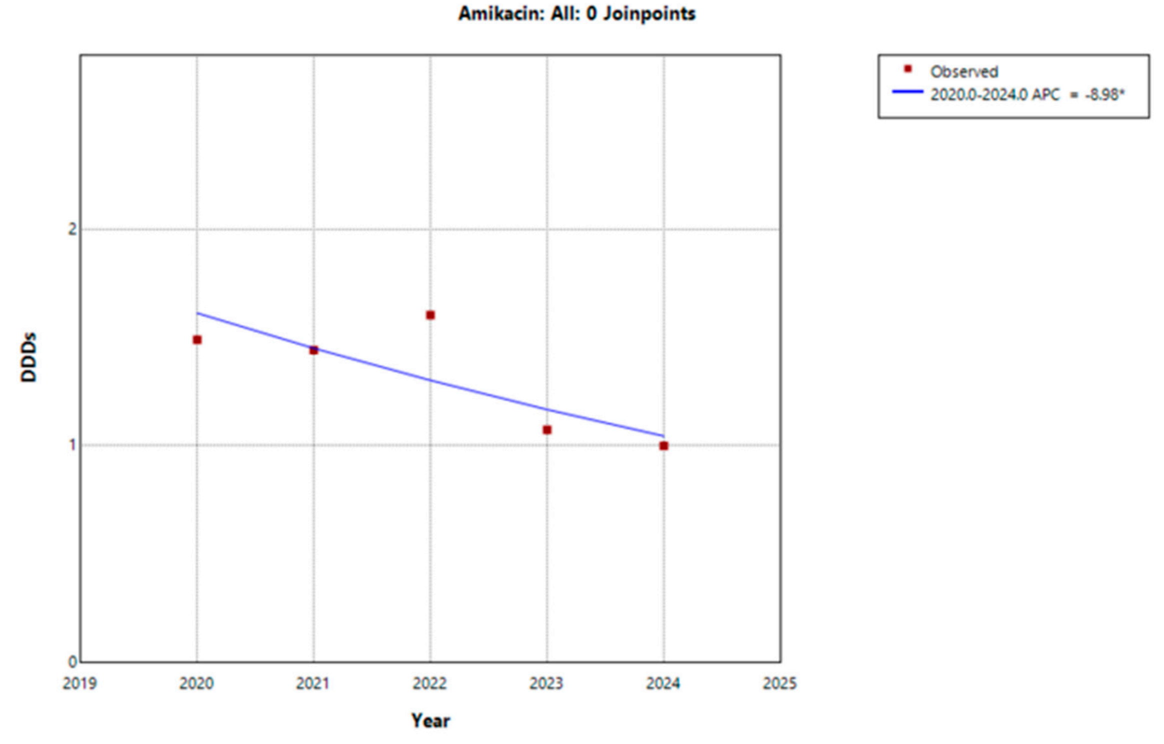

Annual Percent Change (APC)

Segment	Lower Endpoint	Upper Endpoint	APC	Lower CI	Upper CI	Test Statistic	P-Value
1	2020	2024	-8.9814*	-16.2777	-1.4196	--	0.019196

* Indicates that the Annual Percent Change (APC) is significantly different from zero at the alpha = 0.05 level.
-- Test Statistic not available for the Empirical Quantile method. See Help to Learn More

Average Annual Percent Change (AAPC)

Range	Lower Endpoint	Upper Endpoint	AAPC	Lower CI	Upper CI	Test Statistic	P-Value
Full Range	2020	2024	-8.9814*	-16.2777	-1.4196	--	0.019196

* Indicates that the AAPC is significantly different from zero at the alpha = 0.05 level.
-- Test Statistic not available for the Empirical Quantile method. See Help to Learn More

Figure S2. Amikacin 2020-2024 consumption trend.

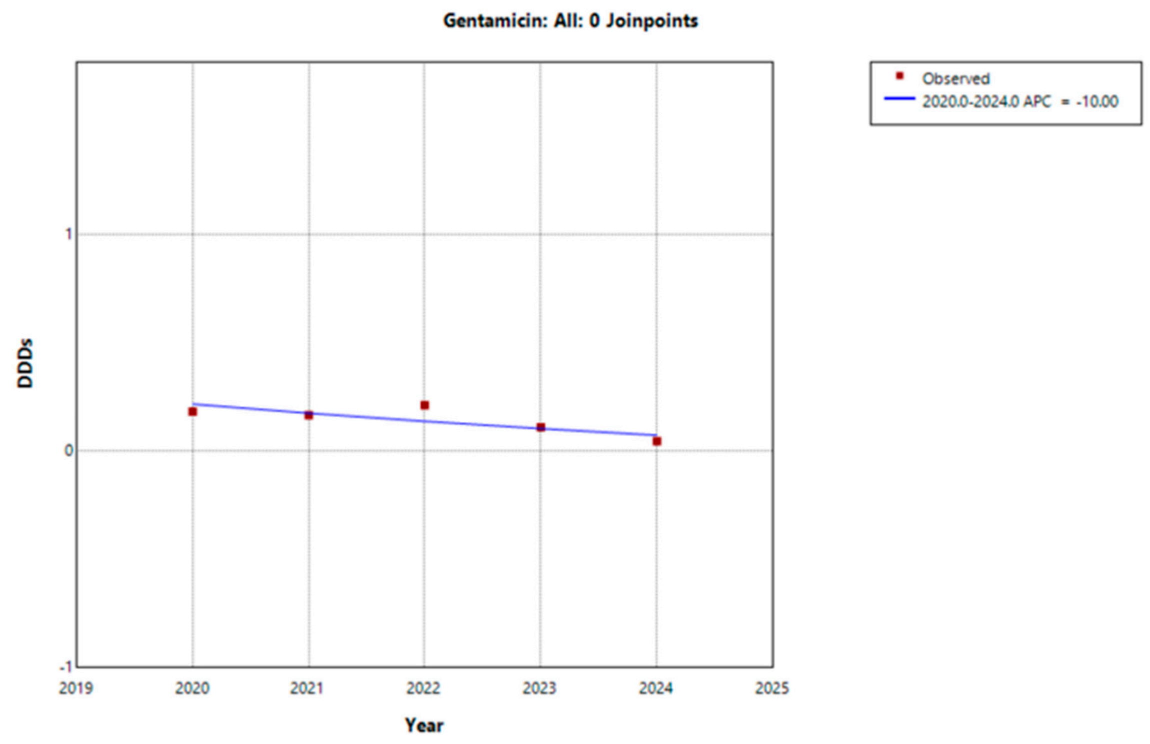

Annual Percent Change (APC)

Segment	Lower Endpoint	Upper Endpoint	APC	Lower CI	Upper CI	Test Statistic	P-Value
1	2020	2024	-9.9984	-20.1144	0.8644	--	0.075585

* Indicates that the Annual Percent Change (APC) is significantly different from zero at the alpha = 0.05 level.
-- Test Statistic not available for the Empirical Quantile method. See Help to Learn More

Average Annual Percent Change (AAPC)

Range	Lower Endpoint	Upper Endpoint	AAPC	Lower CI	Upper CI	Test Statistic	P-Value
Full Range	2020	2024	-9.9984	-20.1144	0.8644	--	0.075585

* Indicates that the AAPC is significantly different from zero at the alpha = 0.05 level.
-- Test Statistic not available for the Empirical Quantile method. See Help to Learn More

Figure S3. Gentamicin 2020-2024 consumption trend.

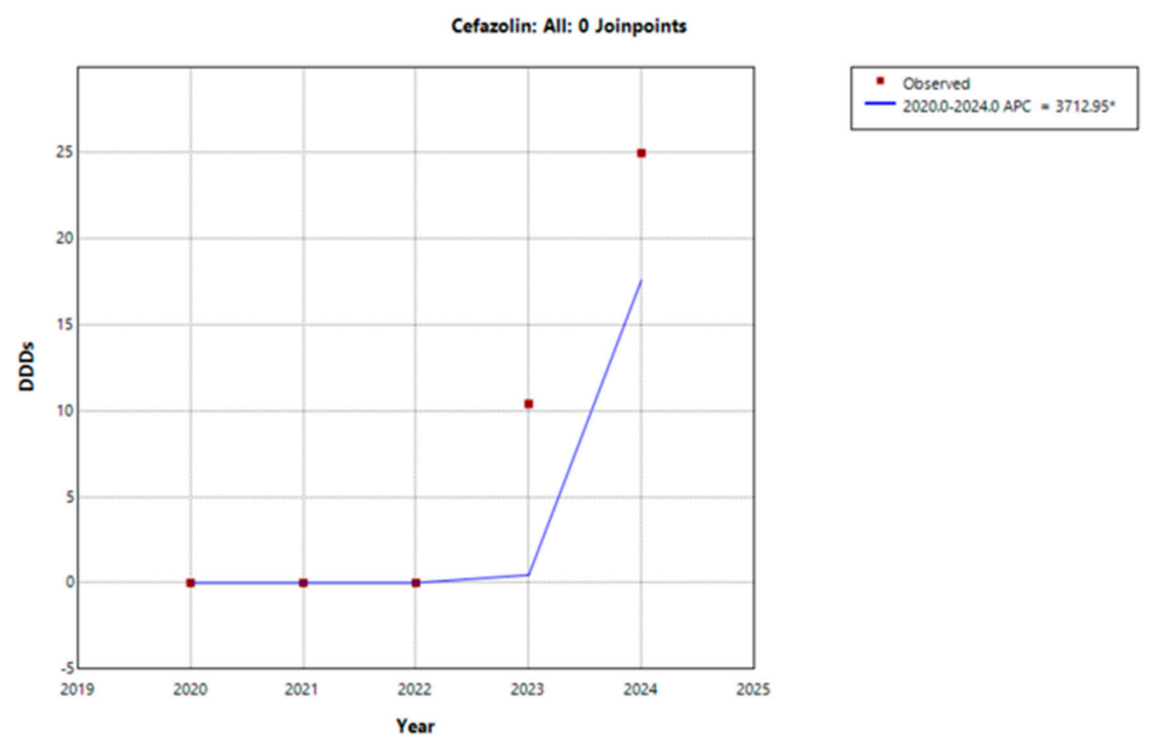

Annual Percent Change (APC)

Segment	Lower Endpoint	Upper Endpoint	APC	Lower CI	Upper CI	Test Statistic	P-Value
1	2020	2024	3712.9549*	17.9932	112239.4780	--	0.037592

* Indicates that the Annual Percent Change (APC) is significantly different from zero at the alpha = 0.05 level.
-- Test Statistic not available for the Empirical Quantile method. See Help to Learn More

Average Annual Percent Change (AAPC)

Range	Lower Endpoint	Upper Endpoint	AAPC	Lower CI	Upper CI	Test Statistic	P-Value
Full Range	2020	2024	3712.9549*	17.9932	112239.4780	--	0.037592

* Indicates that the AAPC is significantly different from zero at the alpha = 0.05 level.
-- Test Statistic not available for the Empirical Quantile method. See Help to Learn More

Figure S4. Cefazolin 2020-2024 consumption trend.

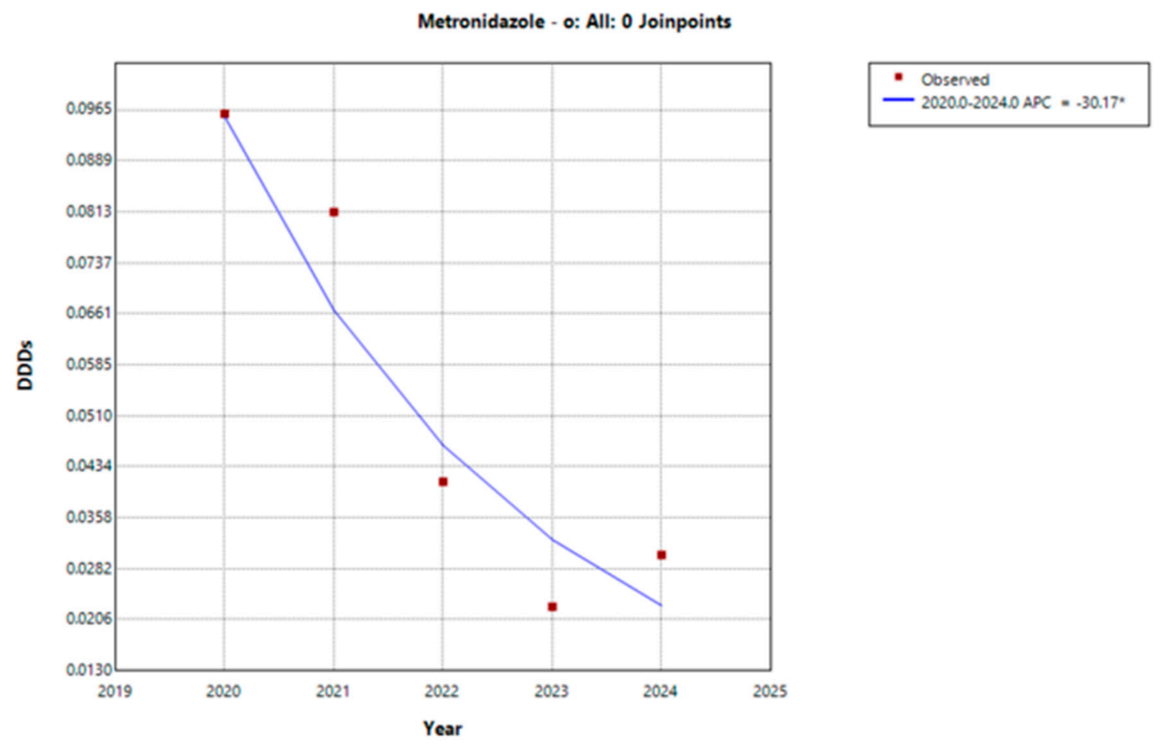

Annual Percent Change (APC)

Segment	Lower Endpoint	Upper Endpoint	APC	Lower CI	Upper CI	Test Statistic	P-Value
1	2020	2024	-30.1677*	-49.3661	-4.2181	--	0.023595

* Indicates that the Annual Percent Change (APC) is significantly different from zero at the alpha = 0.05 level.
-- Test Statistic not available for the Empirical Quantile method. See Help to Learn More

Average Annual Percent Change (AAPC)

Range	Lower Endpoint	Upper Endpoint	AAPC	Lower CI	Upper CI	Test Statistic	P-Value
Full Range	2020	2024	-30.1677*	-49.3661	-4.2181	--	0.023595

* Indicates that the AAPC is significantly different from zero at the alpha = 0.05 level.
-- Test Statistic not available for the Empirical Quantile method. See Help to Learn More

Figure S5. Metronidazole (oral) 2020-2024 consumption trend.

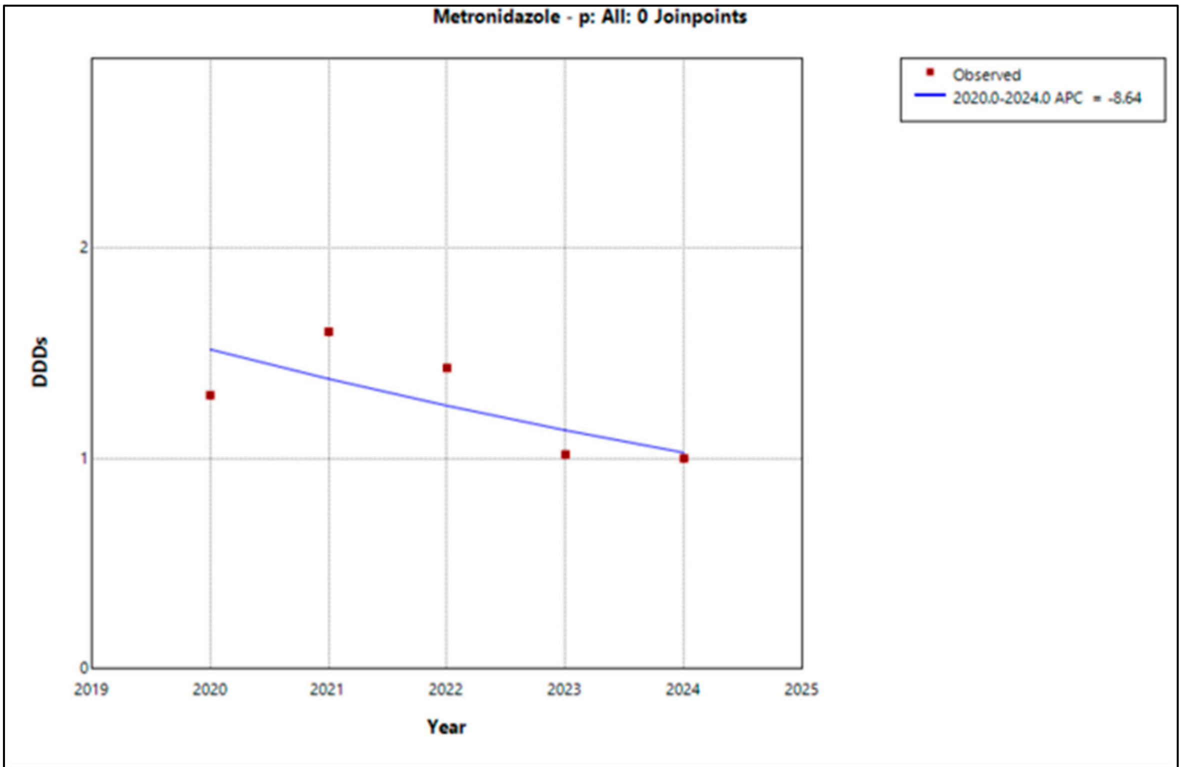

Annual Percent Change (APC)

Segment	Lower Endpoint	Upper Endpoint	APC	Lower CI	Upper CI	Test Statistic	P-Value
1	2020	2024	-8.6440	-26.5821	13.6454	--	0.322735

* Indicates that the Annual Percent Change (APC) is significantly different from zero at the alpha = 0.05 level.
-- Test Statistic not available for the Empirical Quantile method. See Help to Learn More

Average Annual Percent Change (AAPC)

Range	Lower Endpoint	Upper Endpoint	AAPC	Lower CI	Upper CI	Test Statistic	P-Value
Full Range	2020	2024	-8.6440	-26.5821	13.6454	--	0.322735

* Indicates that the AAPC is significantly different from zero at the alpha = 0.05 level.
-- Test Statistic not available for the Empirical Quantile method. See Help to Learn More

Figure S6. Metronidazole (parenteral) 2020-2024 consumption trend.

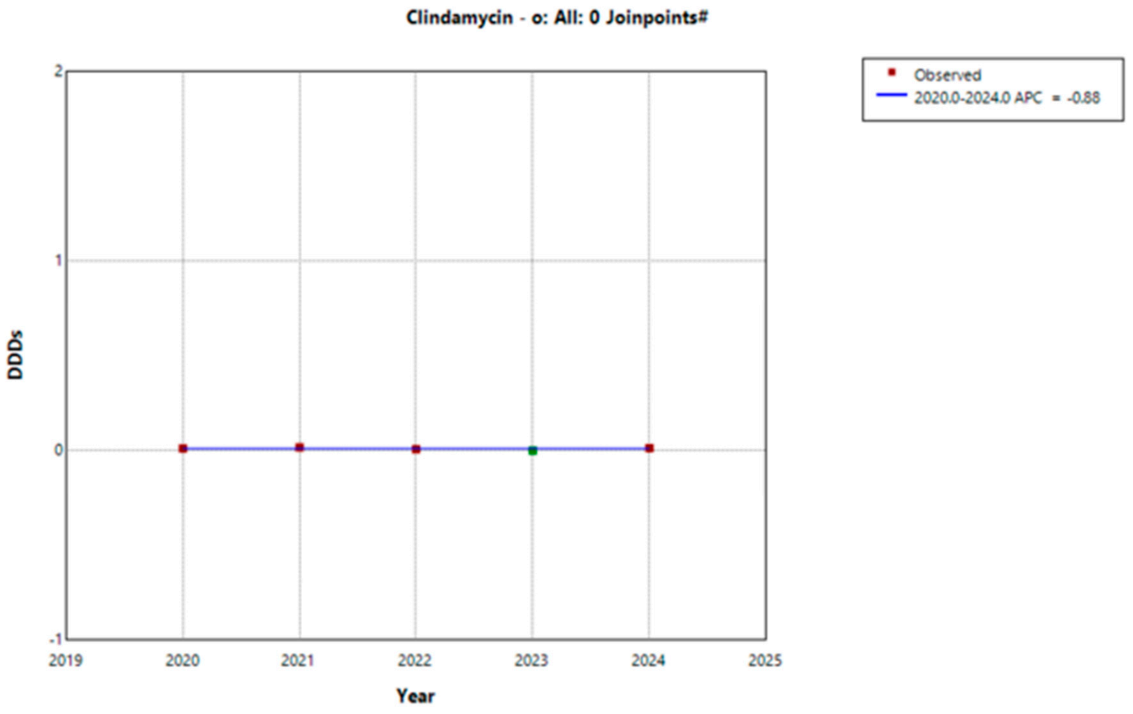

Annual Percent Change (APC)							
Segment	Lower Endpoint	Upper Endpoint	APC	Lower CI	Upper CI	Test Statistic	P-Value
1	2020	2024	-0.8791	-32.9870	40.8405	--	0.991002

* Indicates that the Annual Percent Change (APC) is significantly different from zero at the alpha = 0.05 level.
-- Test Statistic not available for the Empirical Quantile method. See Help to Learn More

Average Annual Percent Change (AAPC)							
Range	Lower Endpoint	Upper Endpoint	AAPC	Lower CI	Upper CI	Test Statistic	P-Value
Full Range	2020	2024	-0.8791	-32.9870	40.8405	--	0.991002

* Indicates that the AAPC is significantly different from zero at the alpha = 0.05 level.
-- Test Statistic not available for the Empirical Quantile method. See Help to Learn More

Figure S7. Clindamycin (oral) 2020-2024 consumption trend.

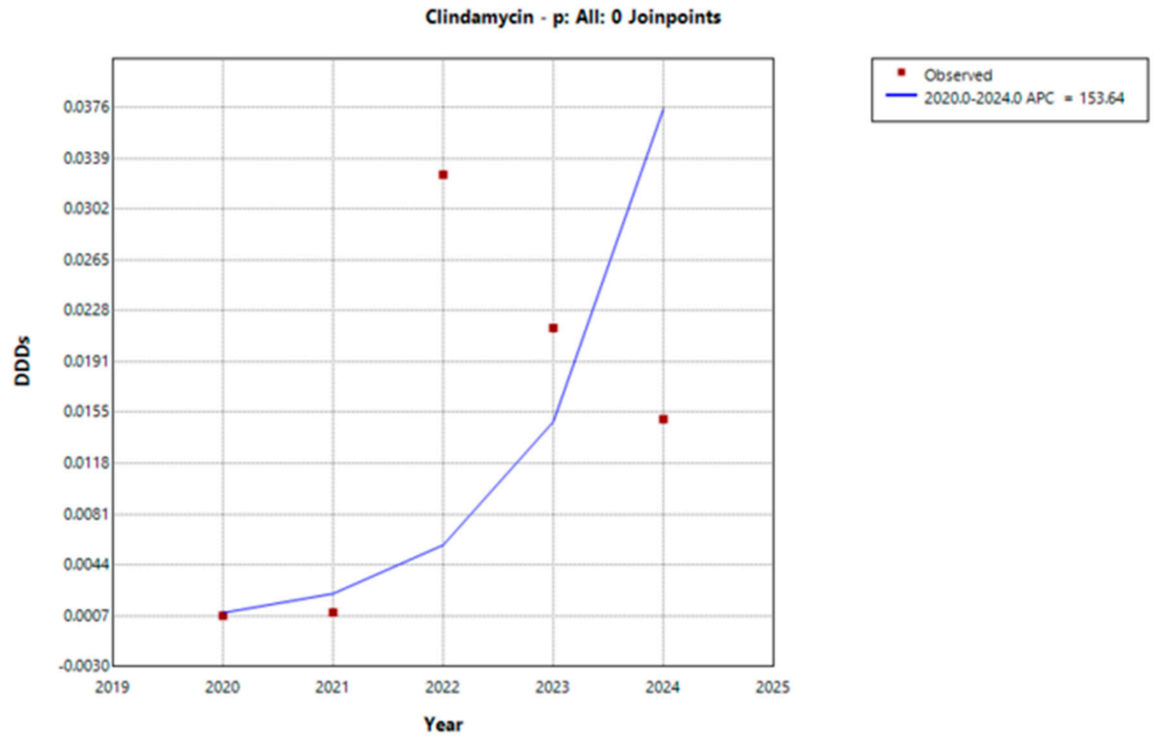

Annual Percent Change (APC)

Segment	Lower Endpoint	Upper Endpoint	APC	Lower CI	Upper CI	Test Statistic	P-Value
1	2020	2024	153.6350	-27.8793	748.5986	--	0.149970

* Indicates that the Annual Percent Change (APC) is significantly different from zero at the alpha = 0.05 level.
-- Test Statistic not available for the Empirical Quantile method. See Help to Learn More

Average Annual Percent Change (AAPC)

Range	Lower Endpoint	Upper Endpoint	AAPC	Lower CI	Upper CI	Test Statistic	P-Value
Full Range	2020	2024	153.6350	-27.8793	748.5986	--	0.149970

* Indicates that the AAPC is significantly different from zero at the alpha = 0.05 level.
-- Test Statistic not available for the Empirical Quantile method. See Help to Learn More

Figure S8. Clindamycin (parenteral) 2020-2024 consumption trend.

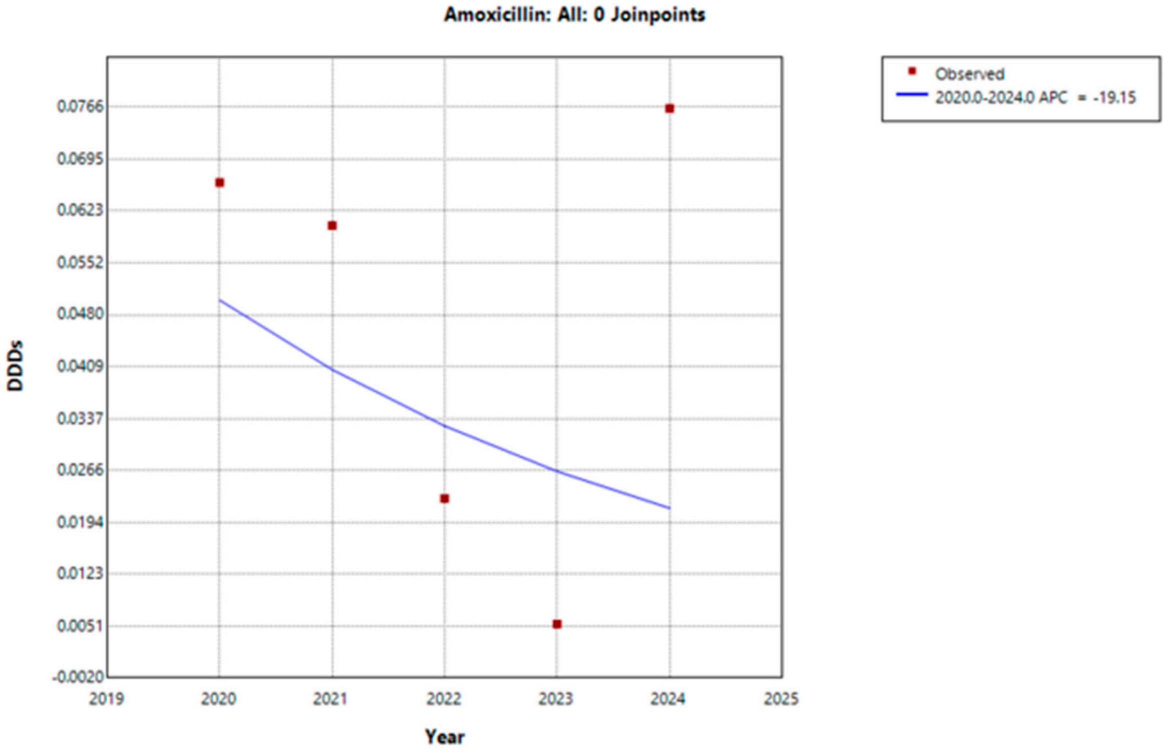

Annual Percent Change (APC)

Segment	Lower Endpoint	Upper Endpoint	APC	Lower CI	Upper CI	Test Statistic	P-Value
1	2020	2024	-19.1541	-69.2394	105.6639	--	0.633073

* Indicates that the Annual Percent Change (APC) is significantly different from zero at the alpha = 0.05 level.
-- Test Statistic not available for the Empirical Quantile method. See Help to Learn More

Average Annual Percent Change (AAPC)

Range	Lower Endpoint	Upper Endpoint	AAPC	Lower CI	Upper CI	Test Statistic	P-Value
Full Range	2020	2024	-19.1541	-69.2394	105.6639	--	0.633073

* Indicates that the AAPC is significantly different from zero at the alpha = 0.05 level.
-- Test Statistic not available for the Empirical Quantile method. See Help to Learn More

Figure S9. Amoxicillin 2020-2024 consumption trend.

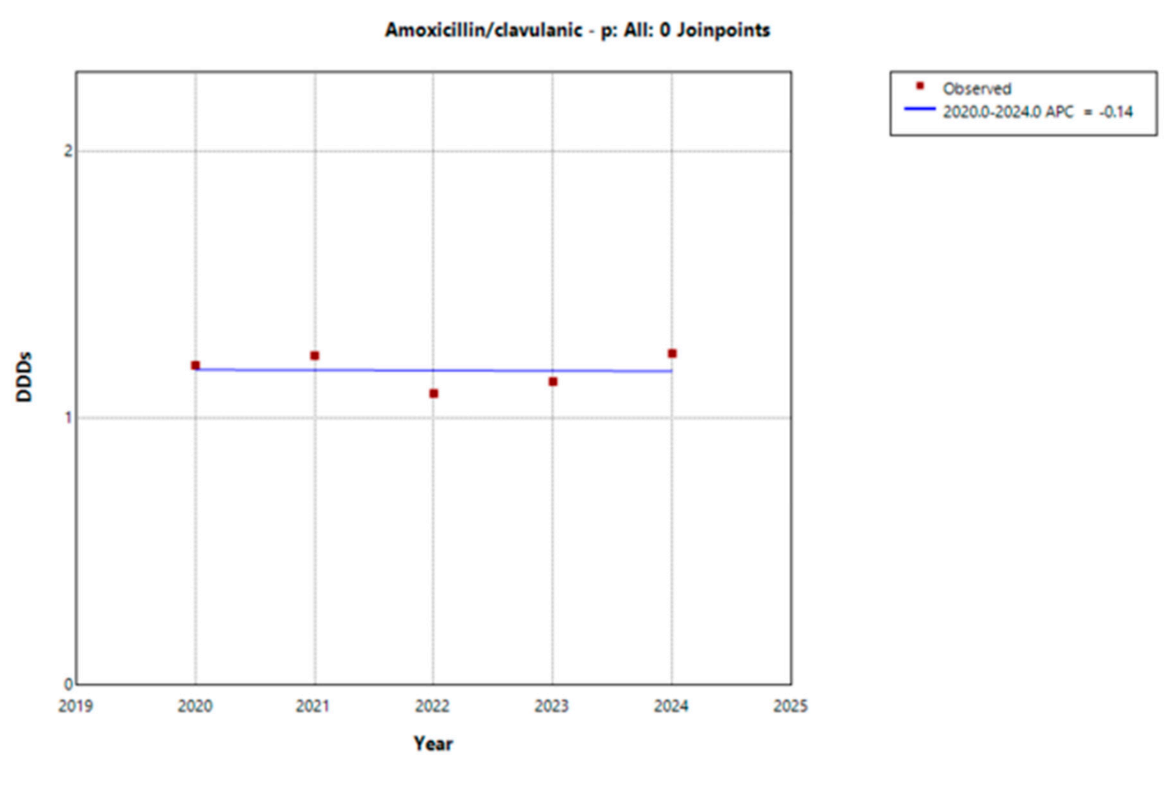

Annual Percent Change (APC)

Segment	Lower Endpoint	Upper Endpoint	APC	Lower CI	Upper CI	Test Statistic	P-Value
1	2020	2024	-0.1442	-8.1577	8.3607	--	0.961008

* Indicates that the Annual Percent Change (APC) is significantly different from zero at the alpha = 0.05 level.
-- Test Statistic not available for the Empirical Quantile method. See Help to Learn More

Average Annual Percent Change (AAPC)

Range	Lower Endpoint	Upper Endpoint	AAPC	Lower CI	Upper CI	Test Statistic	P-Value
Full Range	2020	2024	-0.1442	-8.1577	8.3607	--	0.961008

* Indicates that the AAPC is significantly different from zero at the alpha = 0.05 level.
-- Test Statistic not available for the Empirical Quantile method. See Help to Learn More

Figure S10. Amoxicillin/clavulanic acid (parenteral) 2020-2024 consumption trend.

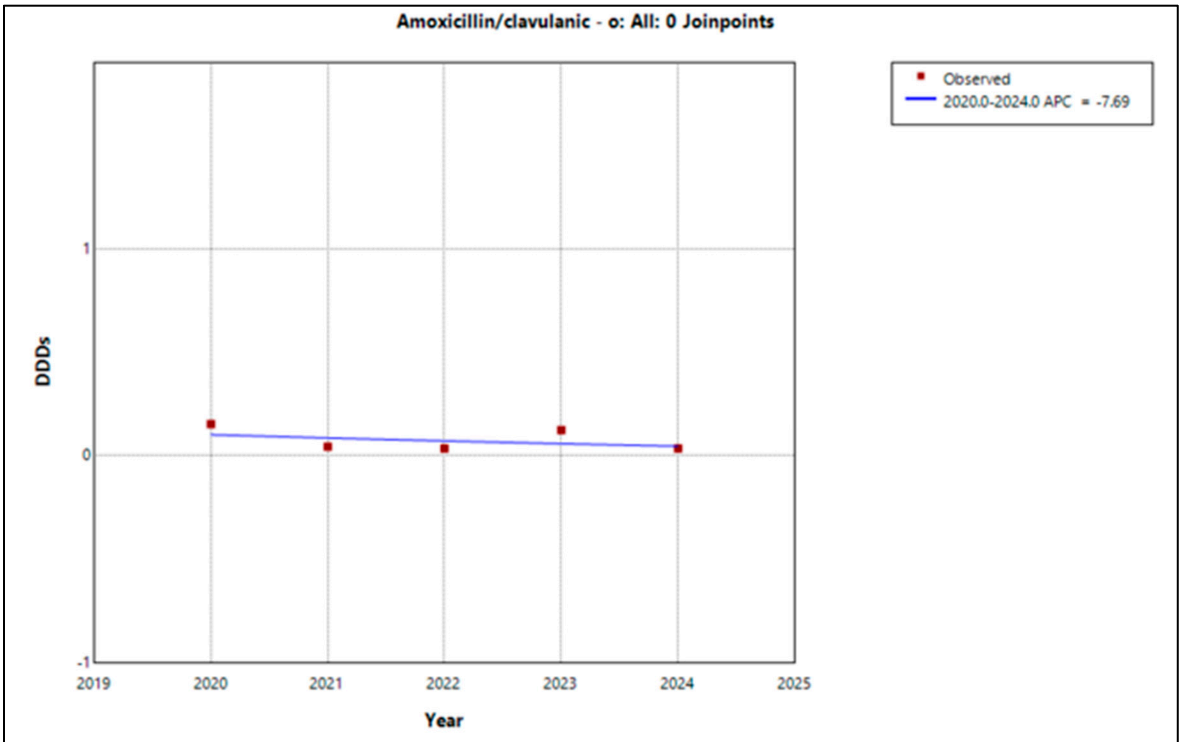

Annual Percent Change (APC)

Segment	Lower Endpoint	Upper Endpoint	APC	Lower CI	Upper CI	Test Statistic	P-Value
1	2020	2024	-7.6890	-35.3678	30.6586	--	0.555889

* Indicates that the Annual Percent Change (APC) is significantly different from zero at the alpha = 0.05 level.
-- Test Statistic not available for the Empirical Quantile method. See Help to Learn More

Average Annual Percent Change (AAPC)

Range	Lower Endpoint	Upper Endpoint	AAPC	Lower CI	Upper CI	Test Statistic	P-Value
Full Range	2020	2024	-7.6890	-35.3678	30.6586	--	0.555889

* Indicates that the AAPC is significantly different from zero at the alpha = 0.05 level.
-- Test Statistic not available for the Empirical Quantile method. See Help to Learn More

Figure S11. Amoxicillin/clavulanic acid (oral) 2020-2024 consumption trend.

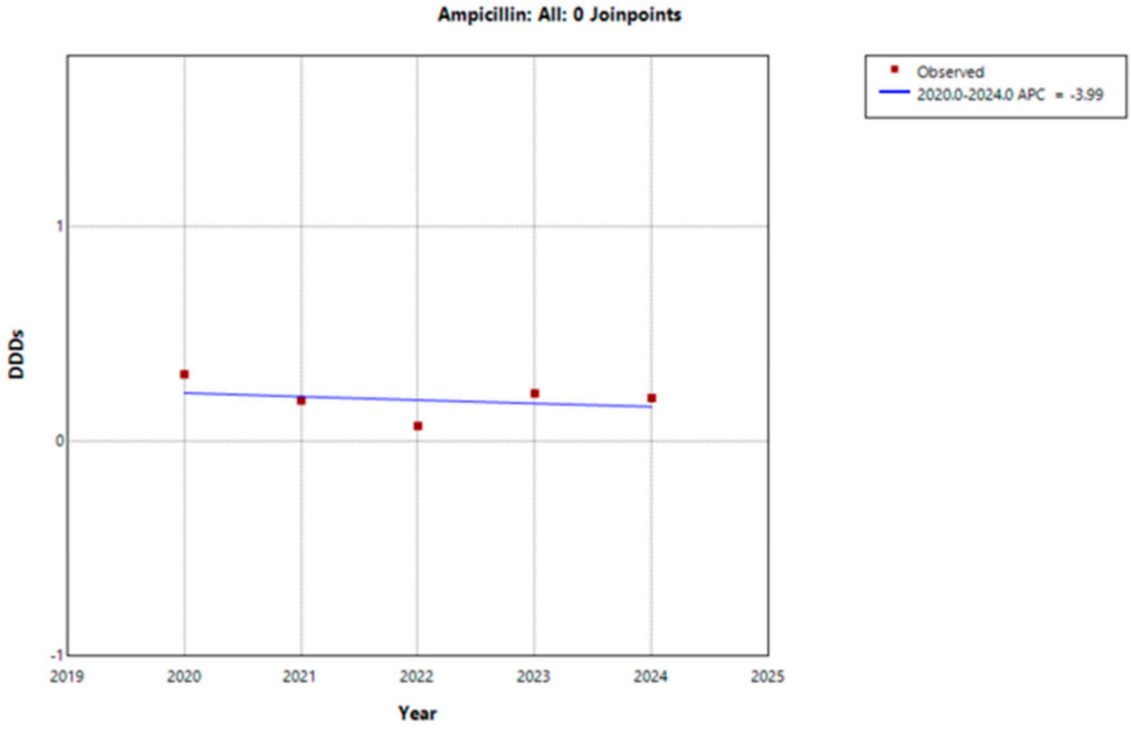

Annual Percent Change (APC)

Segment	Lower Endpoint	Upper Endpoint	APC	Lower CI	Upper CI	Test Statistic	P-Value
1	2020	2024	-3.9941	-18.4237	12.6515	--	0.618276

* Indicates that the Annual Percent Change (APC) is significantly different from zero at the alpha = 0.05 level.
-- Test Statistic not available for the Empirical Quantile method. See Help to Learn More

Average Annual Percent Change (AAPC)

Range	Lower Endpoint	Upper Endpoint	AAPC	Lower CI	Upper CI	Test Statistic	P-Value
Full Range	2020	2024	-3.9941	-18.4237	12.6515	--	0.618276

* Indicates that the AAPC is significantly different from zero at the alpha = 0.05 level.
-- Test Statistic not available for the Empirical Quantile method. See Help to Learn More

Figure S12. Ampicillin 2020-2024 consumption trend.

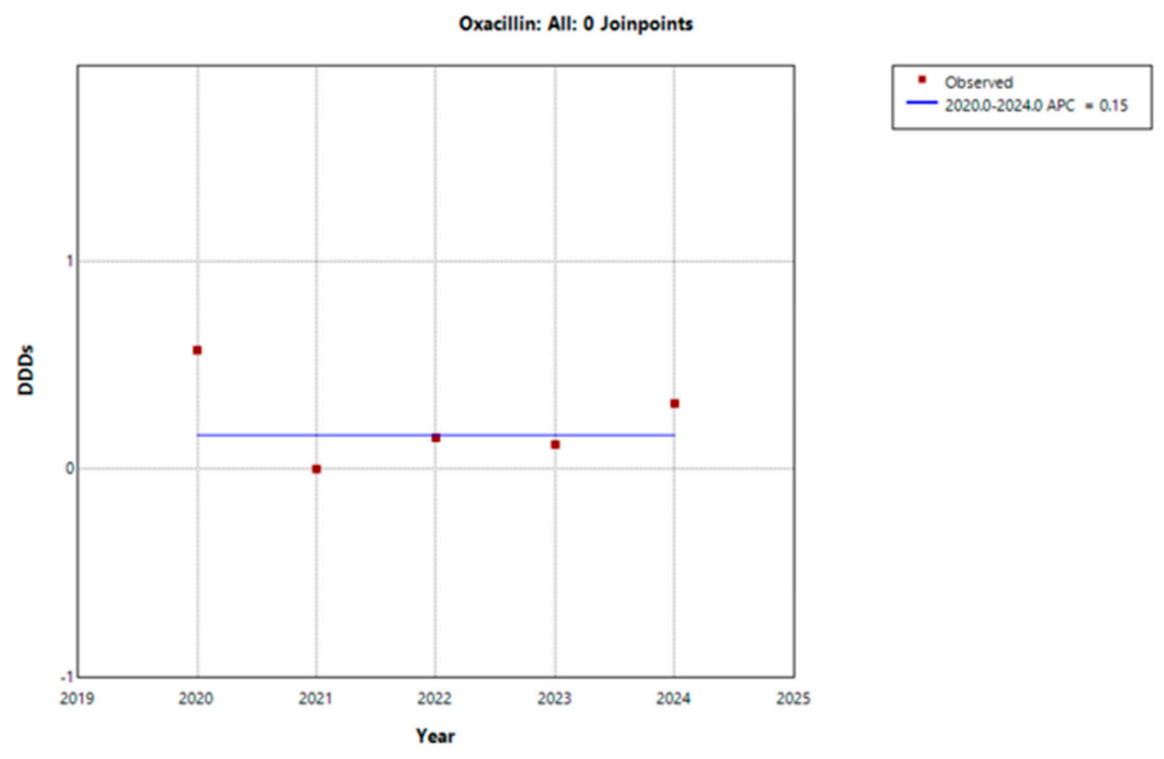

Annual Percent Change (APC)

Segment	Lower Endpoint	Upper Endpoint	APC	Lower CI	Upper CI	Test Statistic	P-Value
1	2020	2024	0.1480	-61.1626	153.0422	--	0.976205

* Indicates that the Annual Percent Change (APC) is significantly different from zero at the alpha = 0.05 level.
-- Test Statistic not available for the Empirical Quantile method. See Help to Learn More

Average Annual Percent Change (AAPC)

Range	Lower Endpoint	Upper Endpoint	AAPC	Lower CI	Upper CI	Test Statistic	P-Value
Full Range	2020	2024	0.1480	-61.1626	153.0422	--	0.976205

* Indicates that the AAPC is significantly different from zero at the alpha = 0.05 level.
-- Test Statistic not available for the Empirical Quantile method. See Help to Learn More

Figure S13. Oxacillin 2020-2024 consumption trend.

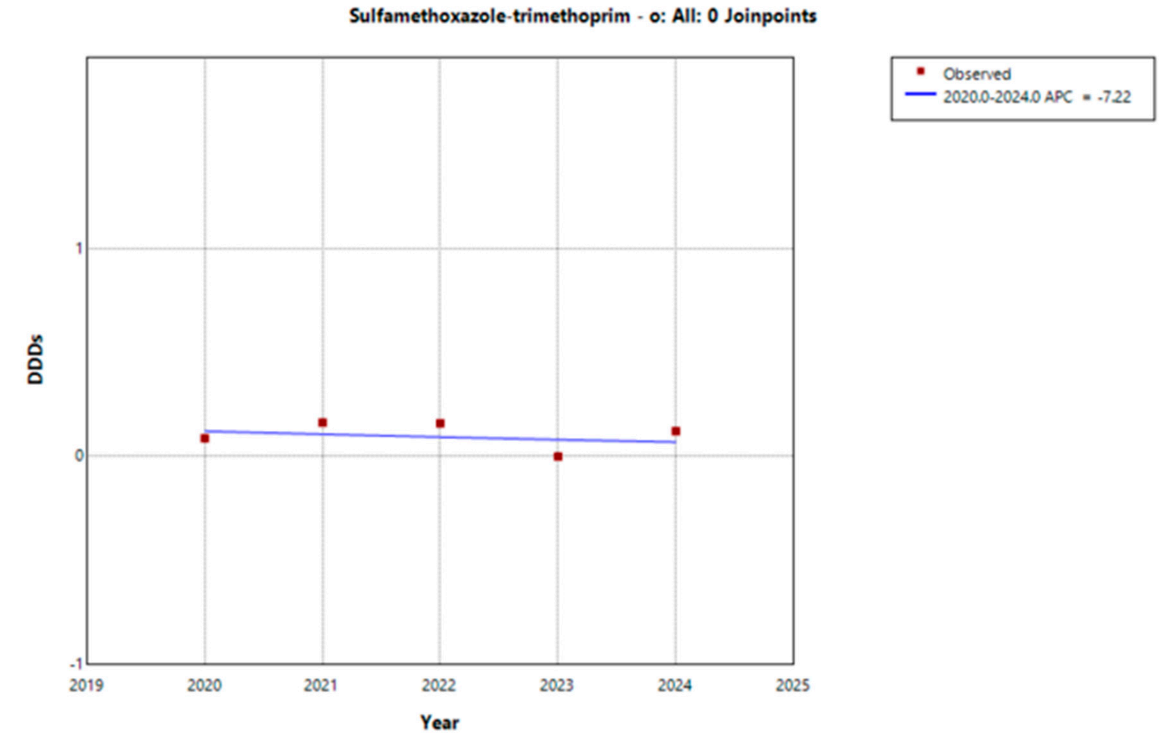

Annual Percent Change (APC)

Segment	Lower Endpoint	Upper Endpoint	APC	Lower CI	Upper CI	Test Statistic	P-Value
1	2020	2024	-7.2233	-34.3191	30.7205	--	0.691862

* Indicates that the Annual Percent Change (APC) is significantly different from zero at the alpha = 0.05 level.
-- Test Statistic not available for the Empirical Quantile method. See Help to Learn More

Average Annual Percent Change (AAPC)

Range	Lower Endpoint	Upper Endpoint	AAPC	Lower CI	Upper CI	Test Statistic	P-Value
Full Range	2020	2024	-7.2233	-34.3191	30.7205	--	0.691862

* Indicates that the AAPC is significantly different from zero at the alpha = 0.05 level.
-- Test Statistic not available for the Empirical Quantile method. See Help to Learn More

Figure S14. Sulfamethoxazole/trimetoprim (oral) 2020-2024 consumption trend.

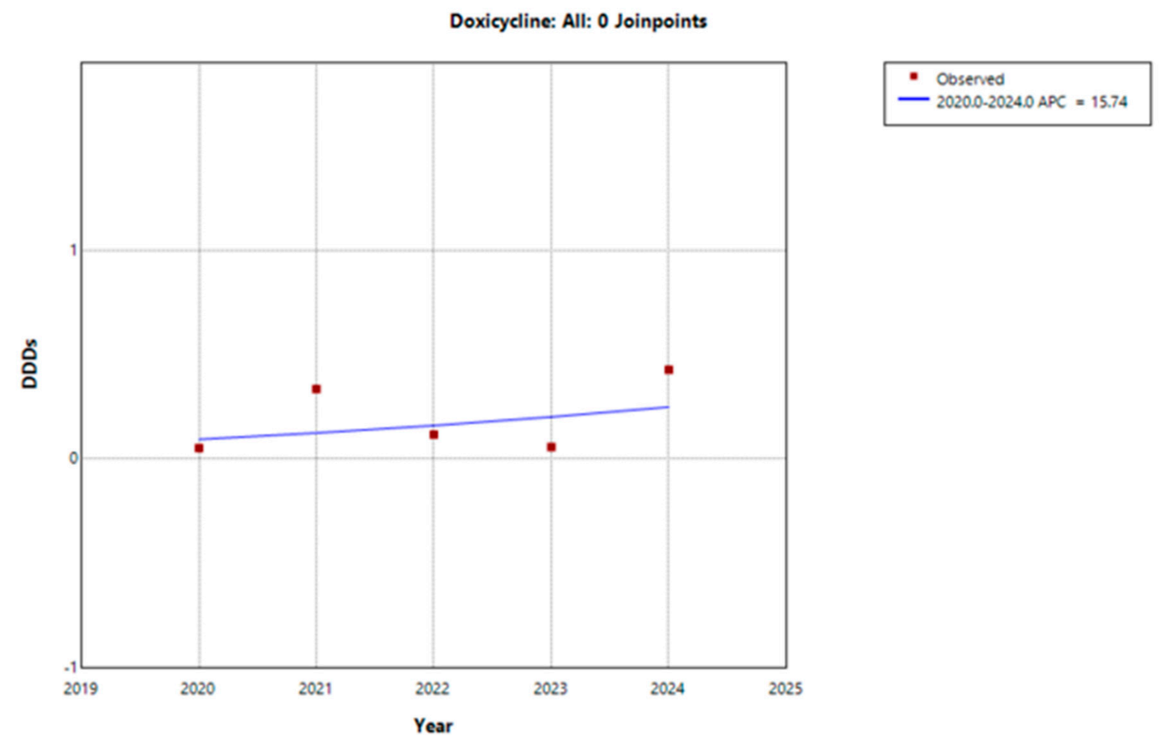

Annual Percent Change (APC)

Segment	Lower Endpoint	Upper Endpoint	APC	Lower CI	Upper CI	Test Statistic	P-Value
1	2020	2024	15.7356	-51.6385	170.6241	--	0.637073

* Indicates that the Annual Percent Change (APC) is significantly different from zero at the alpha = 0.05 level.
-- Test Statistic not available for the Empirical Quantile method. See Help to Learn More

Average Annual Percent Change (AAPC)

Range	Lower Endpoint	Upper Endpoint	AAPC	Lower CI	Upper CI	Test Statistic	P-Value
Full Range	2020	2024	15.7356	-51.6385	170.6241	--	0.637073

* Indicates that the AAPC is significantly different from zero at the alpha = 0.05 level.
-- Test Statistic not available for the Empirical Quantile method. See Help to Learn More

Figure S15. Doxicycline 2020-2024 consumption trend.

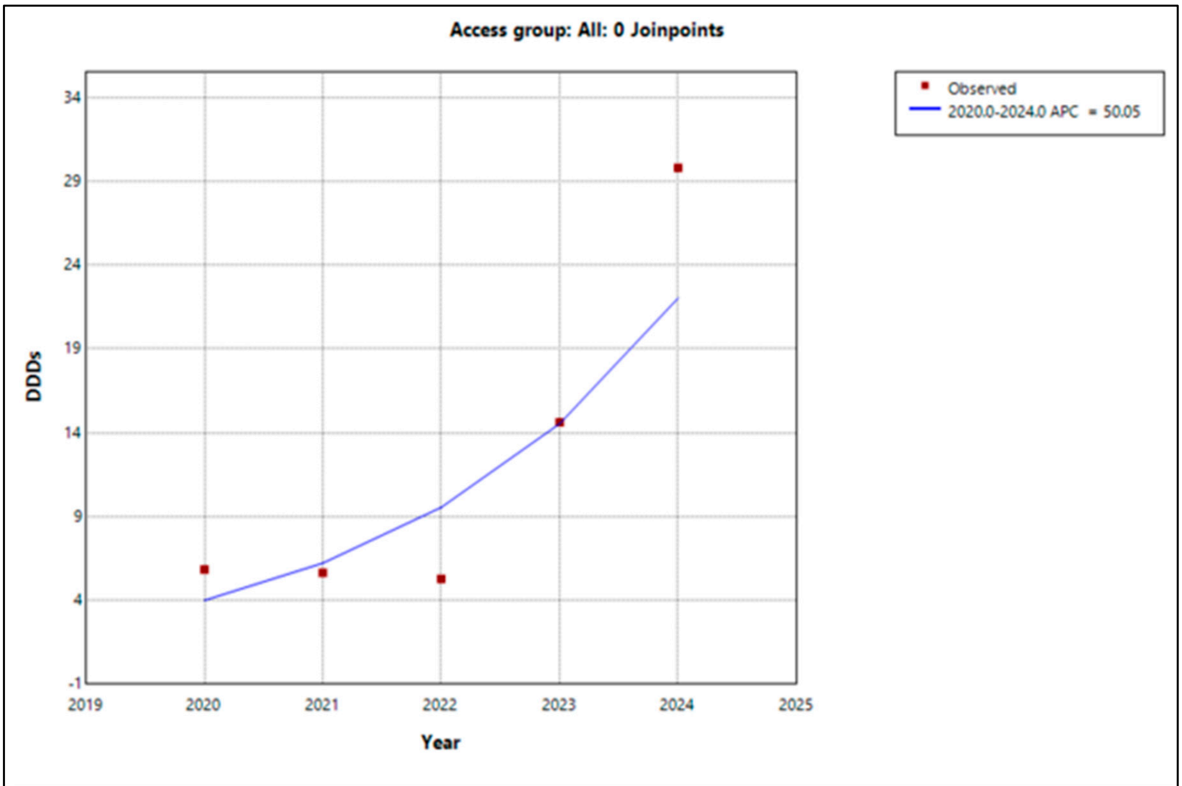

Annual Percent Change (APC)

Segment	Lower Endpoint	Upper Endpoint	APC	Lower CI	Upper CI	Test Statistic	P-Value
1	2020	2024	50.0503	-7.7151	141.0656	--	0.117576

* Indicates that the Annual Percent Change (APC) is significantly different from zero at the alpha = 0.05 level.
-- Test Statistic not available for the Empirical Quantile method. See Help to Learn More

Average Annual Percent Change (AAPC)

Range	Lower Endpoint	Upper Endpoint	AAPC	Lower CI	Upper CI	Test Statistic	P-Value
Full Range	2020	2024	50.0503	-7.7151	141.0656	--	0.117576

* Indicates that the AAPC is significantly different from zero at the alpha = 0.05 level.
-- Test Statistic not available for the Empirical Quantile method. See Help to Learn More

Figure S16. Access group antibiotics 2020-2024 consumption trend.

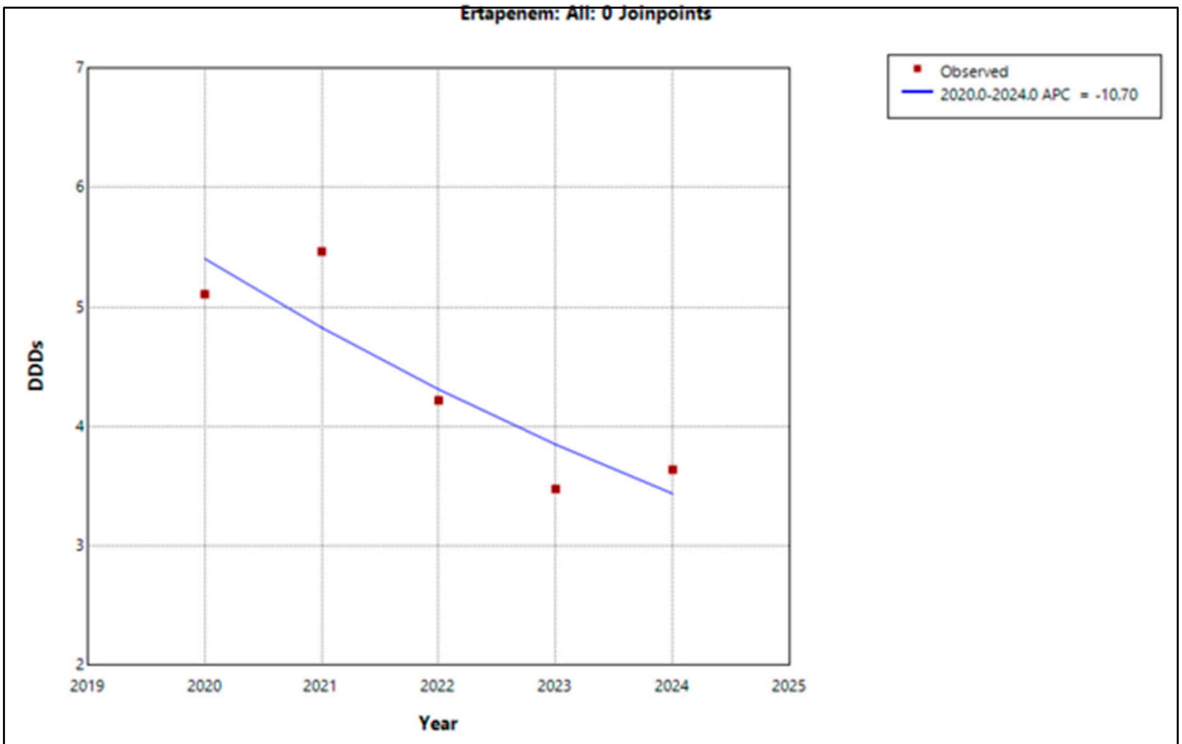

Annual Percent Change (APC)

Segment	Lower Endpoint	Upper Endpoint	APC	Lower CI	Upper CI	Test Statistic	P-Value
1	2020	2024	-10.6962	-22.5656	2.7743	--	0.113177

* Indicates that the Annual Percent Change (APC) is significantly different from zero at the alpha = 0.05 level.
-- Test Statistic not available for the Empirical Quantile method. See Help to Learn More

Average Annual Percent Change (AAPC)

Range	Lower Endpoint	Upper Endpoint	AAPC	Lower CI	Upper CI	Test Statistic	P-Value
Full Range	2020	2024	-10.6962	-22.5656	2.7743	--	0.113177

* Indicates that the AAPC is significantly different from zero at the alpha = 0.05 level.
-- Test Statistic not available for the Empirical Quantile method. See Help to Learn More

Figure S17. Ertapenem 2020-2024 consumption trend.

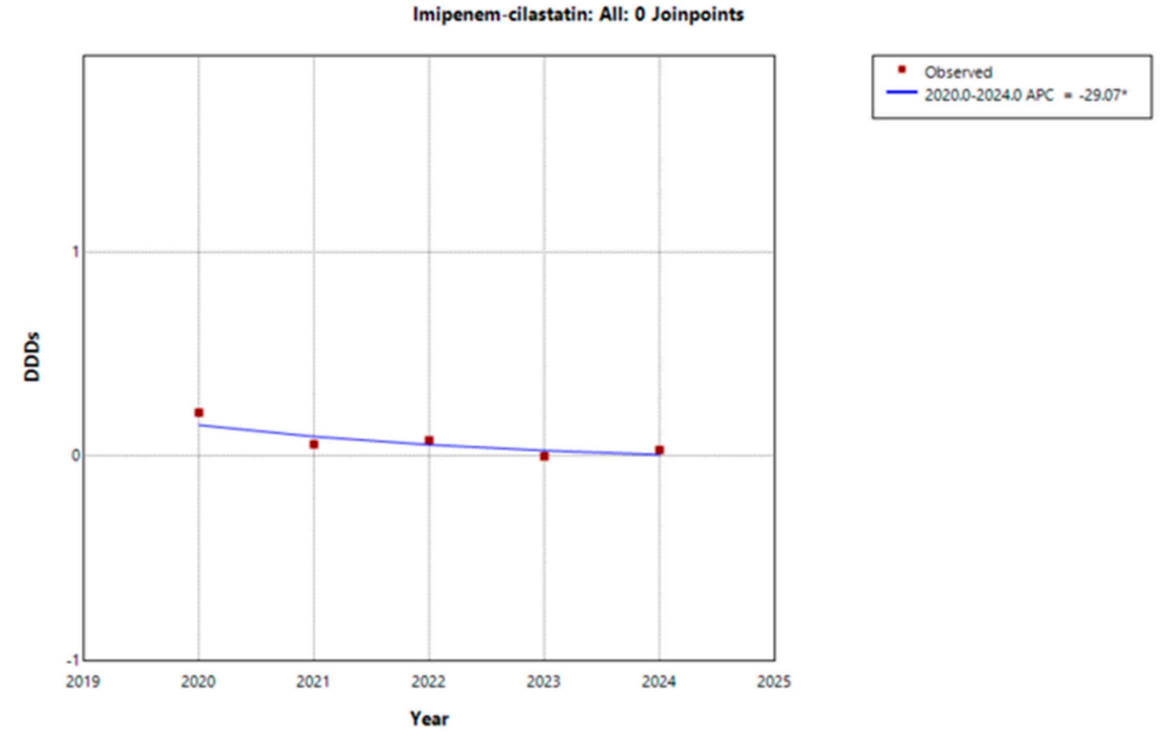

Annual Percent Change (APC)

Segment	Lower Endpoint	Upper Endpoint	APC	Lower CI	Upper CI	Test Statistic	P-Value
1	2020	2024	-29.0669*	-45.9786	-7.6364	--	< 0.000001

* Indicates that the Annual Percent Change (APC) is significantly different from zero at the alpha = 0.05 level.
-- Test Statistic not available for the Empirical Quantile method. See Help to Learn More

Average Annual Percent Change (AAPC)

Range	Lower Endpoint	Upper Endpoint	AAPC	Lower CI	Upper CI	Test Statistic	P-Value
Full Range	2020	2024	-29.0669*	-45.9786	-7.6364	--	< 0.000001

* Indicates that the AAPC is significantly different from zero at the alpha = 0.05 level.
-- Test Statistic not available for the Empirical Quantile method. See Help to Learn More

Figure S18. Imipenem/cilastatin 2020-2024 consumption trend.

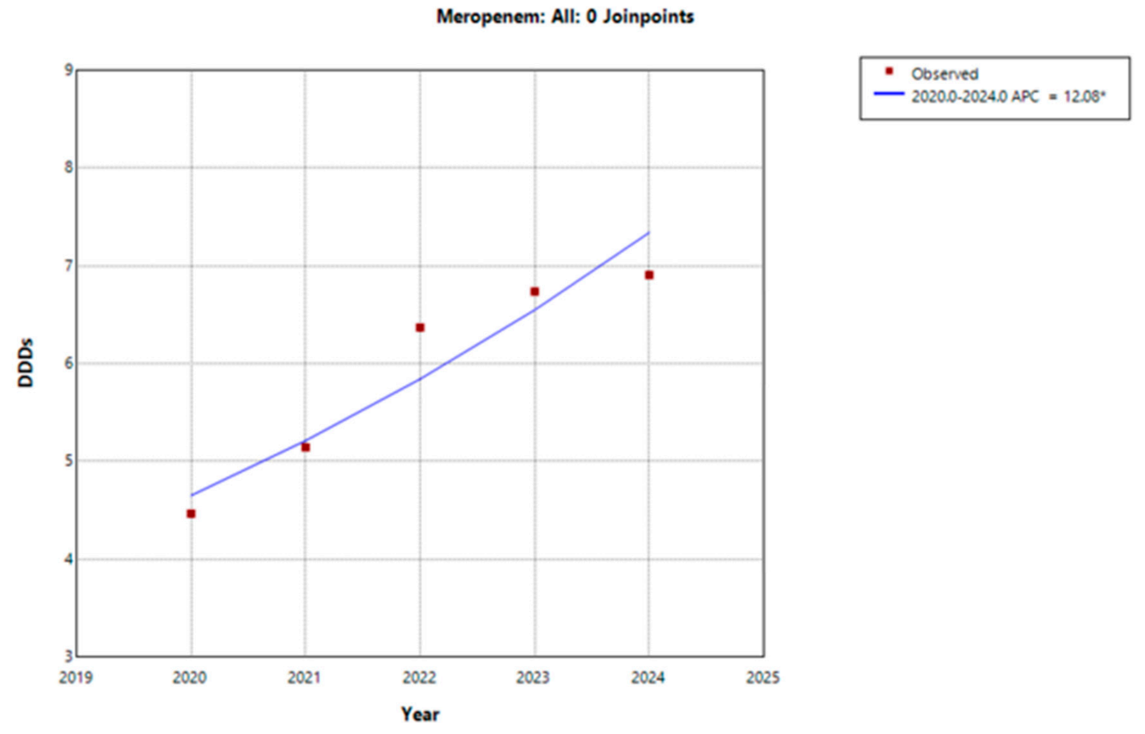

Annual Percent Change (APC)

Segment	Lower Endpoint	Upper Endpoint	APC	Lower CI	Upper CI	Test Statistic	P-Value
1	2020	2024	12.0784*	3.7147	20.7388	--	0.001600

* Indicates that the Annual Percent Change (APC) is significantly different from zero at the alpha = 0.05 level.
-- Test Statistic not available for the Empirical Quantile method. See Help to Learn More

Average Annual Percent Change (AAPC)

Range	Lower Endpoint	Upper Endpoint	AAPC	Lower CI	Upper CI	Test Statistic	P-Value
Full Range	2020	2024	12.0784*	3.7147	20.7388	--	0.001600

* Indicates that the AAPC is significantly different from zero at the alpha = 0.05 level.
-- Test Statistic not available for the Empirical Quantile method. See Help to Learn More

Figure S19. Meropenem 2020-2024 consumption trend.

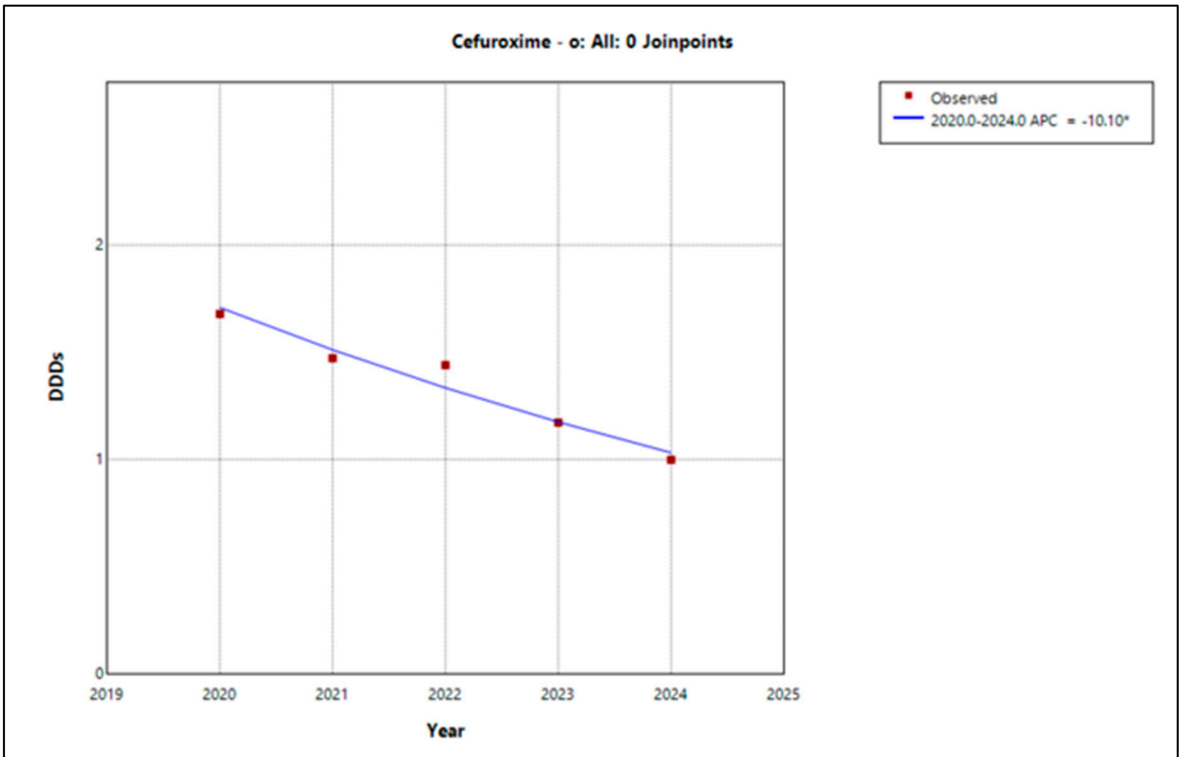

Annual Percent Change (APC)

Segment	Lower Endpoint	Upper Endpoint	APC	Lower CI	Upper CI	Test Statistic	P-Value
1	2020	2024	-10.1041*	-12.6265	-7.6460	--	< 0.000001

* Indicates that the Annual Percent Change (APC) is significantly different from zero at the alpha = 0.05 level.
-- Test Statistic not available for the Empirical Quantile method. See Help to Learn More

Average Annual Percent Change (AAPC)

Range	Lower Endpoint	Upper Endpoint	AAPC	Lower CI	Upper CI	Test Statistic	P-Value
Full Range	2020	2024	-10.1041*	-12.6265	-7.6460	--	< 0.000001

* Indicates that the AAPC is significantly different from zero at the alpha = 0.05 level.
-- Test Statistic not available for the Empirical Quantile method. See Help to Learn More

Figure S20. Cefuroxime (oral) 2020-2024 consumption trend.

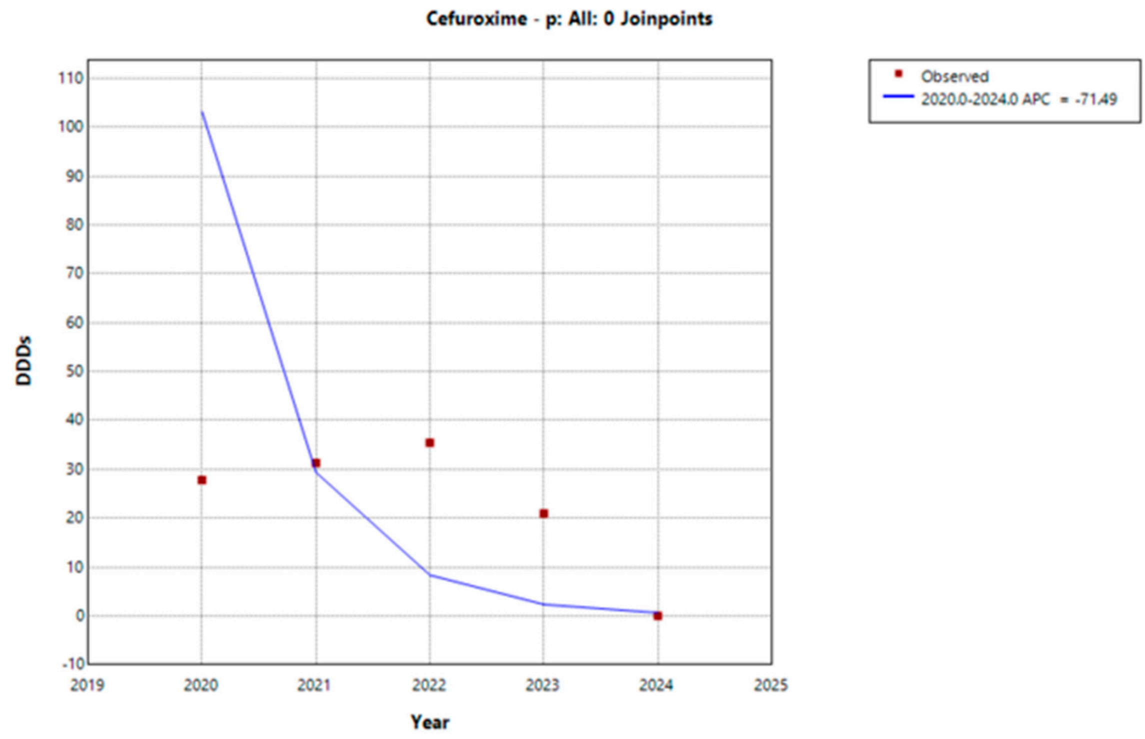

Annual Percent Change (APC)

Segment	Lower Endpoint	Upper Endpoint	APC	Lower CI	Upper CI	Test Statistic	P-Value
1	2020	2024	-71.4890	-97.2386	178.4962	--	0.247550

* Indicates that the Annual Percent Change (APC) is significantly different from zero at the alpha = 0.05 level.
-- Test Statistic not available for the Empirical Quantile method. See Help to Learn More

Average Annual Percent Change (AAPC)

Range	Lower Endpoint	Upper Endpoint	AAPC	Lower CI	Upper CI	Test Statistic	P-Value
Full Range	2020	2024	-71.4890	-97.2386	178.4962	--	0.247550

* Indicates that the AAPC is significantly different from zero at the alpha = 0.05 level.
-- Test Statistic not available for the Empirical Quantile method. See Help to Learn More

Figure S21. Cefuroxime (parenteral) 2020-2024 consumption trend.

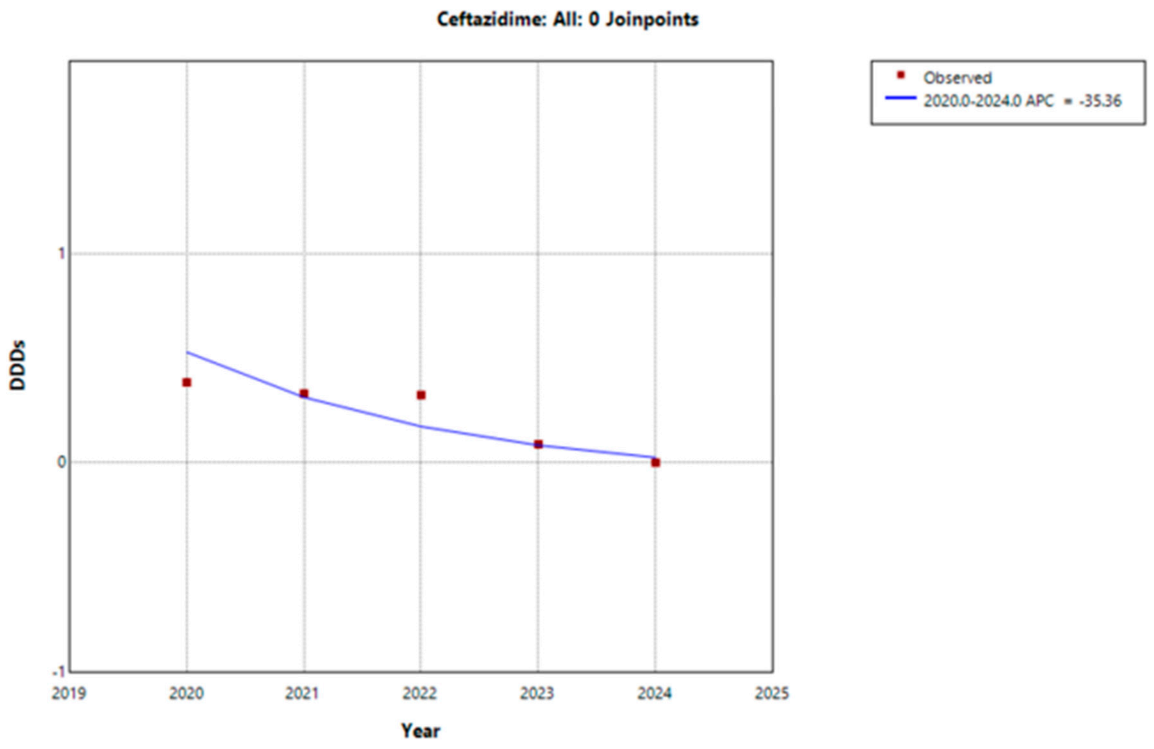

Annual Percent Change (APC)

Segment	Lower Endpoint	Upper Endpoint	APC	Lower CI	Upper CI	Test Statistic	P-Value
1	2020	2024	-35.3604	-61.2553	5.9314	--	0.081584

* Indicates that the Annual Percent Change (APC) is significantly different from zero at the alpha = 0.05 level.
-- Test Statistic not available for the Empirical Quantile method. See Help to Learn More

Average Annual Percent Change (AAPC)

Range	Lower Endpoint	Upper Endpoint	AAPC	Lower CI	Upper CI	Test Statistic	P-Value
Full Range	2020	2024	-35.3604	-61.2553	5.9314	--	0.081584

* Indicates that the AAPC is significantly different from zero at the alpha = 0.05 level.
-- Test Statistic not available for the Empirical Quantile method. See Help to Learn More

Figure S22. Ceftazidime 2020-2024 consumption trend.

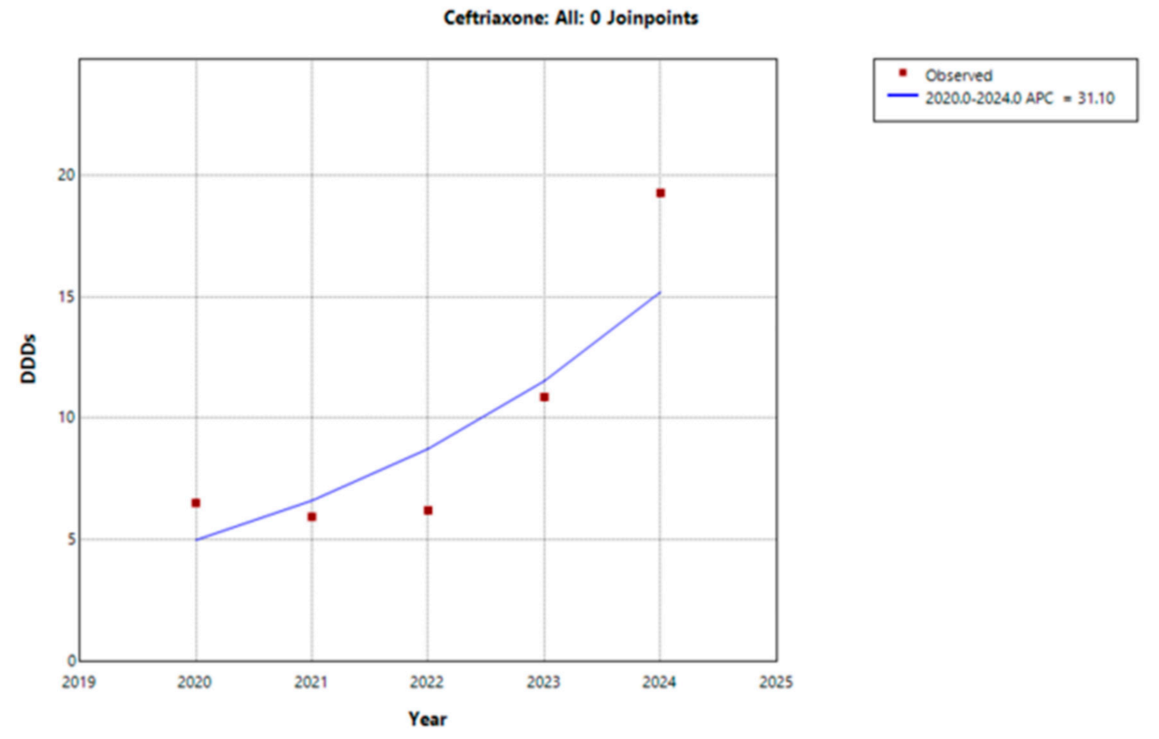

Annual Percent Change (APC)

Segment	Lower Endpoint	Upper Endpoint	APC	Lower CI	Upper CI	Test Statistic	P-Value
1	2020	2024	31.0980	-20.4928	114.3546	--	0.267147

* Indicates that the Annual Percent Change (APC) is significantly different from zero at the alpha = 0.05 level.
-- Test Statistic not available for the Empirical Quantile method. See Help to Learn More

Average Annual Percent Change (AAPC)

Range	Lower Endpoint	Upper Endpoint	AAPC	Lower CI	Upper CI	Test Statistic	P-Value
Full Range	2020	2024	31.0980	-20.4928	114.3546	--	0.267147

* Indicates that the AAPC is significantly different from zero at the alpha = 0.05 level.
-- Test Statistic not available for the Empirical Quantile method. See Help to Learn More

Figure S23. Ceftriaxone 2020-2024 consumption trend.

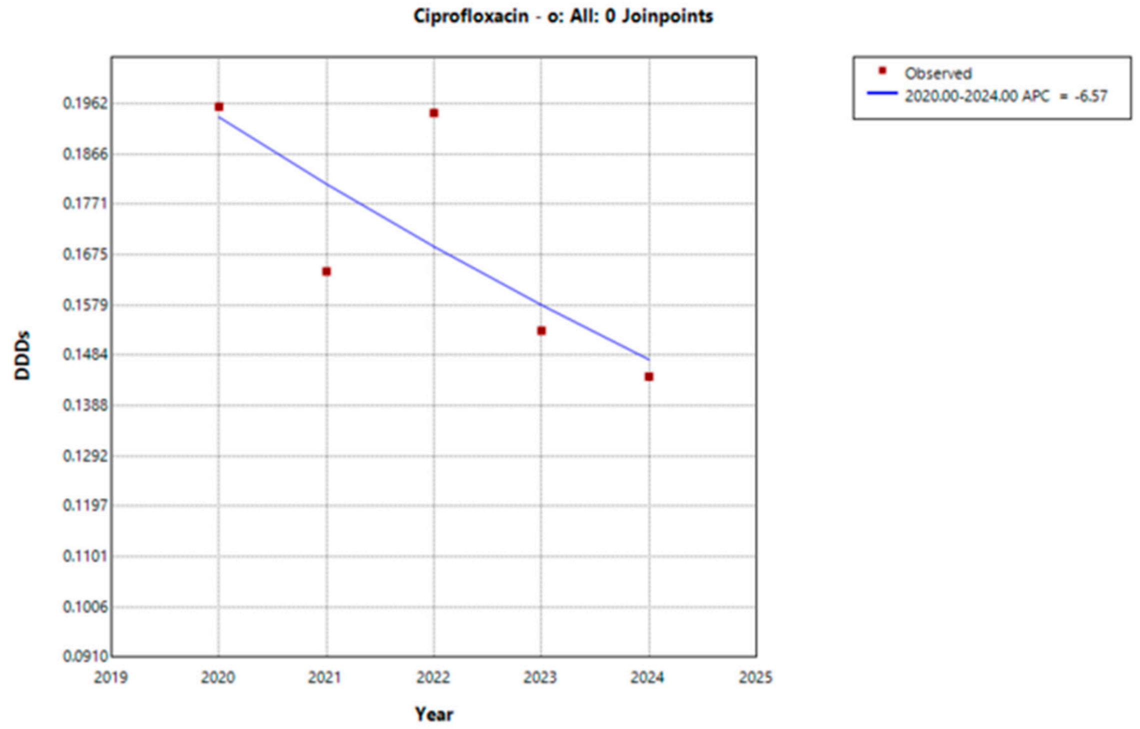

Annual Percent Change (APC)

Segment	Lower Endpoint	Upper Endpoint	APC	Lower CI	Upper CI	Test Statistic	P-Value
1	2020	2024	-6.5713	-12.9967	0.0030	--	0.050390

* Indicates that the Annual Percent Change (APC) is significantly different from zero at the alpha = 0.05 level.
-- Test Statistic not available for the Empirical Quantile method. See Help to Learn More

Average Annual Percent Change (AAPC)

Range	Lower Endpoint	Upper Endpoint	AAPC	Lower CI	Upper CI	Test Statistic	P-Value
Full Range	2020	2024	-6.5713	-12.9967	0.0030	--	0.050390

* Indicates that the AAPC is significantly different from zero at the alpha = 0.05 level.
-- Test Statistic not available for the Empirical Quantile method. See Help to Learn More

Figure S24. Ciprofloxacin (oral) 2020-2024 consumption trend.

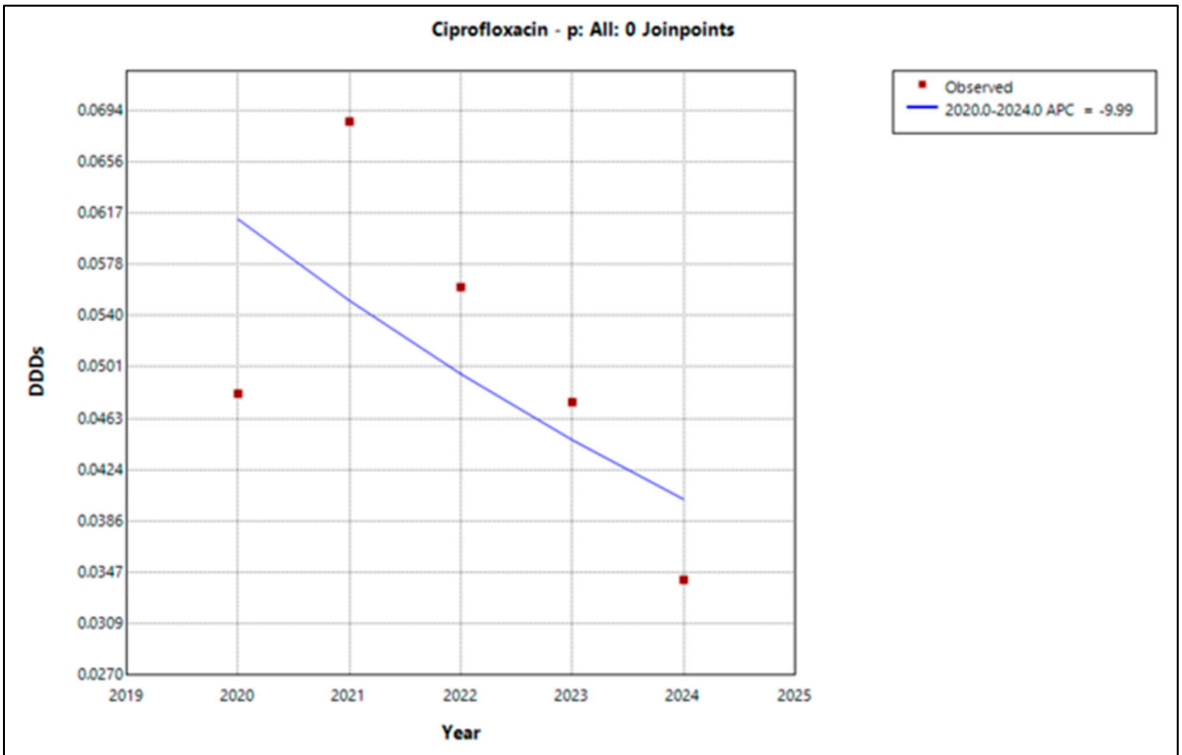

Annual Percent Change (APC)

Segment	Lower Endpoint	Upper Endpoint	APC	Lower CI	Upper CI	Test Statistic	P-Value
1	2020	2024	-9.9898	-29.7975	14.5508	--	0.328734

* Indicates that the Annual Percent Change (APC) is significantly different from zero at the alpha = 0.05 level.
-- Test Statistic not available for the Empirical Quantile method. See Help to Learn More

Average Annual Percent Change (AAPC)

Range	Lower Endpoint	Upper Endpoint	AAPC	Lower CI	Upper CI	Test Statistic	P-Value
Full Range	2020	2024	-9.9898	-29.7975	14.5508	--	0.328734

* Indicates that the AAPC is significantly different from zero at the alpha = 0.05 level.
-- Test Statistic not available for the Empirical Quantile method. See Help to Learn More

Figure S25. Ciprofloxacin (parenteral) 2020-2024 consumption trend.

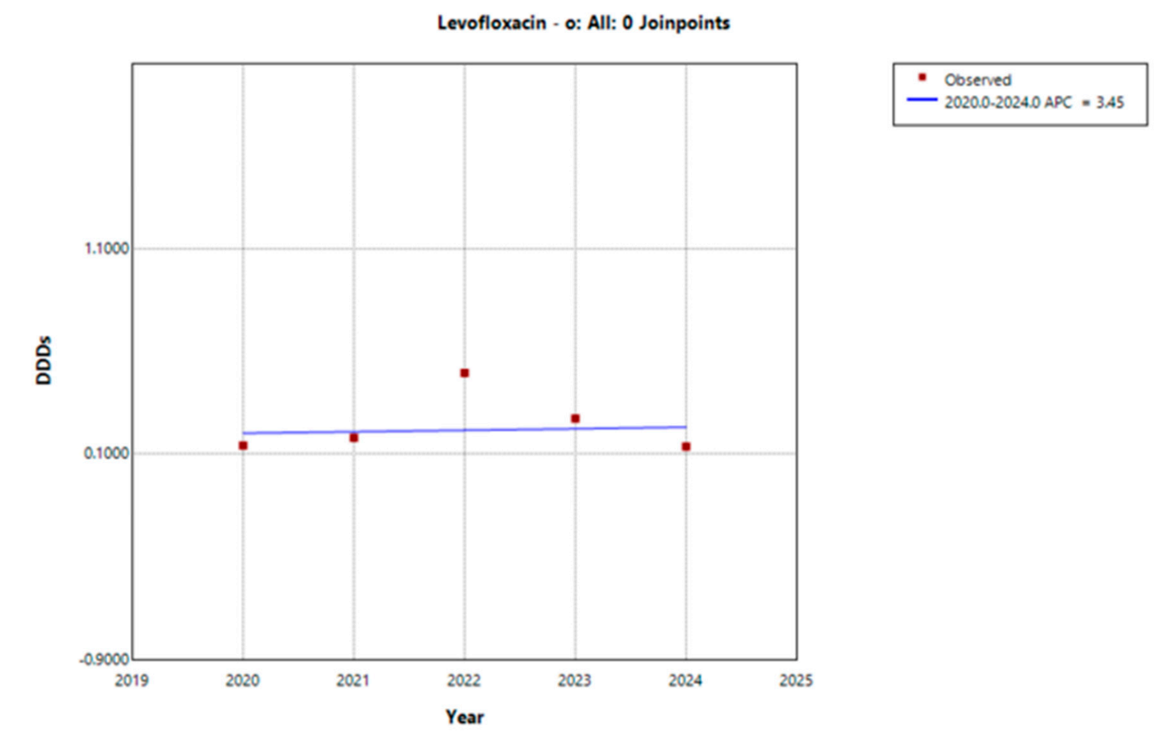

Annual Percent Change (APC)

Segment	Lower Endpoint	Upper Endpoint	APC	Lower CI	Upper CI	Test Statistic	P-Value
1	2020	2024	3.4506	-46.5148	93.9852	--	0.879024

* Indicates that the Annual Percent Change (APC) is significantly different from zero at the alpha = 0.05 level.
-- Test Statistic not available for the Empirical Quantile method. See Help to Learn More

Average Annual Percent Change (AAPC)

Range	Lower Endpoint	Upper Endpoint	AAPC	Lower CI	Upper CI	Test Statistic	P-Value
Full Range	2020	2024	3.4506	-46.5148	93.9852	--	0.879024

* Indicates that the AAPC is significantly different from zero at the alpha = 0.05 level.
-- Test Statistic not available for the Empirical Quantile method. See Help to Learn More

Figure S26. Levofloxacin (oral) 2020-2024 consumption trend.

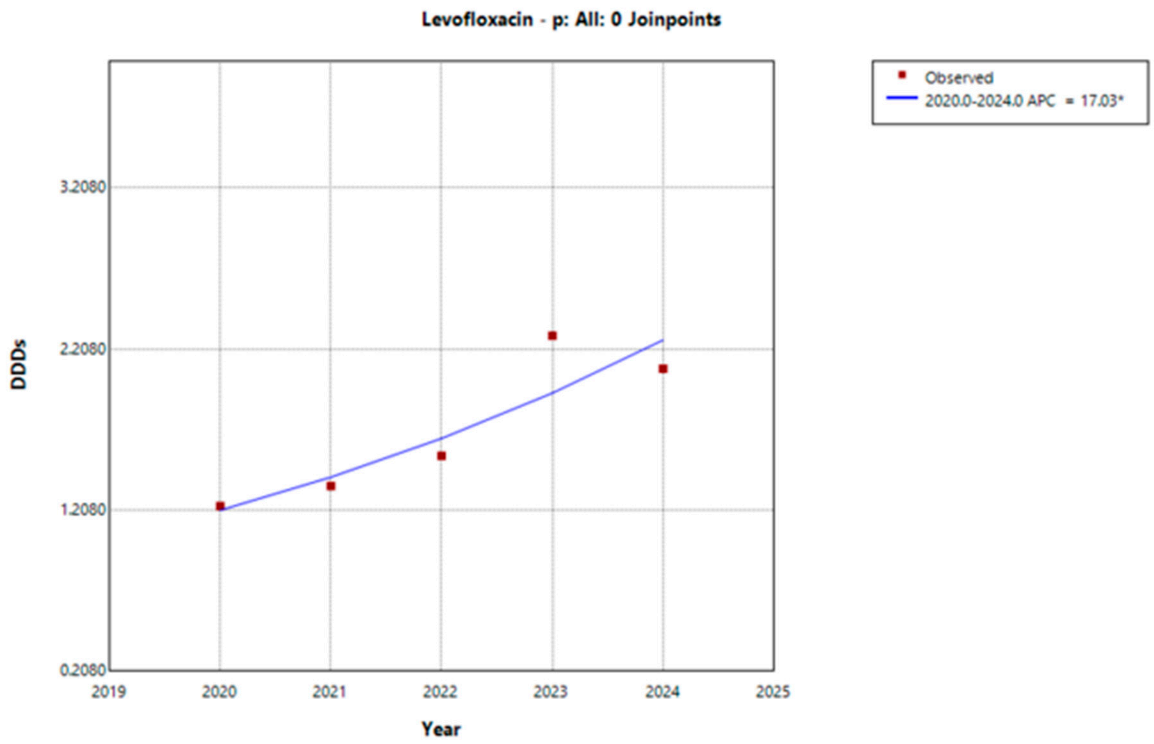

Annual Percent Change (APC)

Segment	Lower Endpoint	Upper Endpoint	APC	Lower CI	Upper CI	Test Statistic	P-Value
1	2020	2024	17.0335*	6.3103	28.2322	--	0.000400

* Indicates that the Annual Percent Change (APC) is significantly different from zero at the alpha = 0.05 level.
-- Test Statistic not available for the Empirical Quantile method. See Help to Learn More

Average Annual Percent Change (AAPC)

Range	Lower Endpoint	Upper Endpoint	AAPC	Lower CI	Upper CI	Test Statistic	P-Value
Full Range	2020	2024	17.0335*	6.3103	28.2322	--	0.000400

* Indicates that the AAPC is significantly different from zero at the alpha = 0.05 level.
-- Test Statistic not available for the Empirical Quantile method. See Help to Learn More

Figure S27. Levofloxacin (parenteral) 2020-2024 consumption trend.

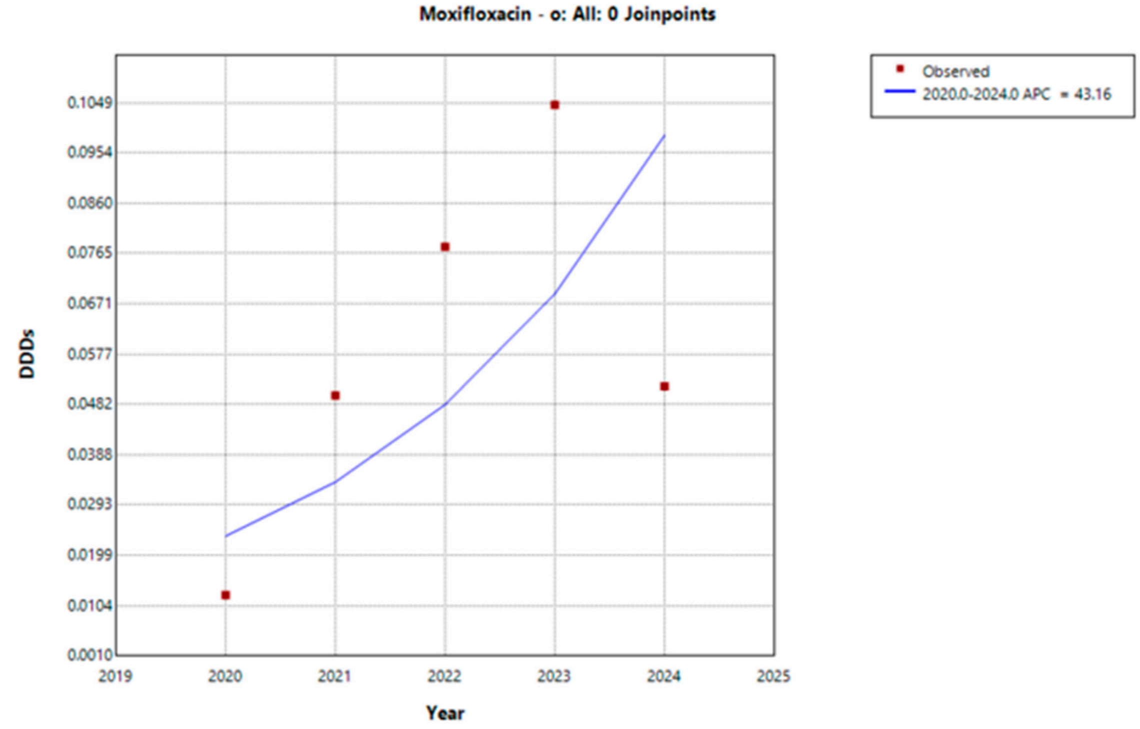

Annual Percent Change (APC)

Segment	Lower Endpoint	Upper Endpoint	APC	Lower CI	Upper CI	Test Statistic	P-Value
1	2020	2024	43.1606	-8.7725	122.7047	--	0.143971

* Indicates that the Annual Percent Change (APC) is significantly different from zero at the alpha = 0.05 level.
-- Test Statistic not available for the Empirical Quantile method. See Help to Learn More

Average Annual Percent Change (AAPC)

Range	Lower Endpoint	Upper Endpoint	AAPC	Lower CI	Upper CI	Test Statistic	P-Value
Full Range	2020	2024	43.1606	-8.7725	122.7047	--	0.143971

* Indicates that the AAPC is significantly different from zero at the alpha = 0.05 level.
-- Test Statistic not available for the Empirical Quantile method. See Help to Learn More

Figure S28. Moxifloxacin (oral) 2020-2024 consumption trend.

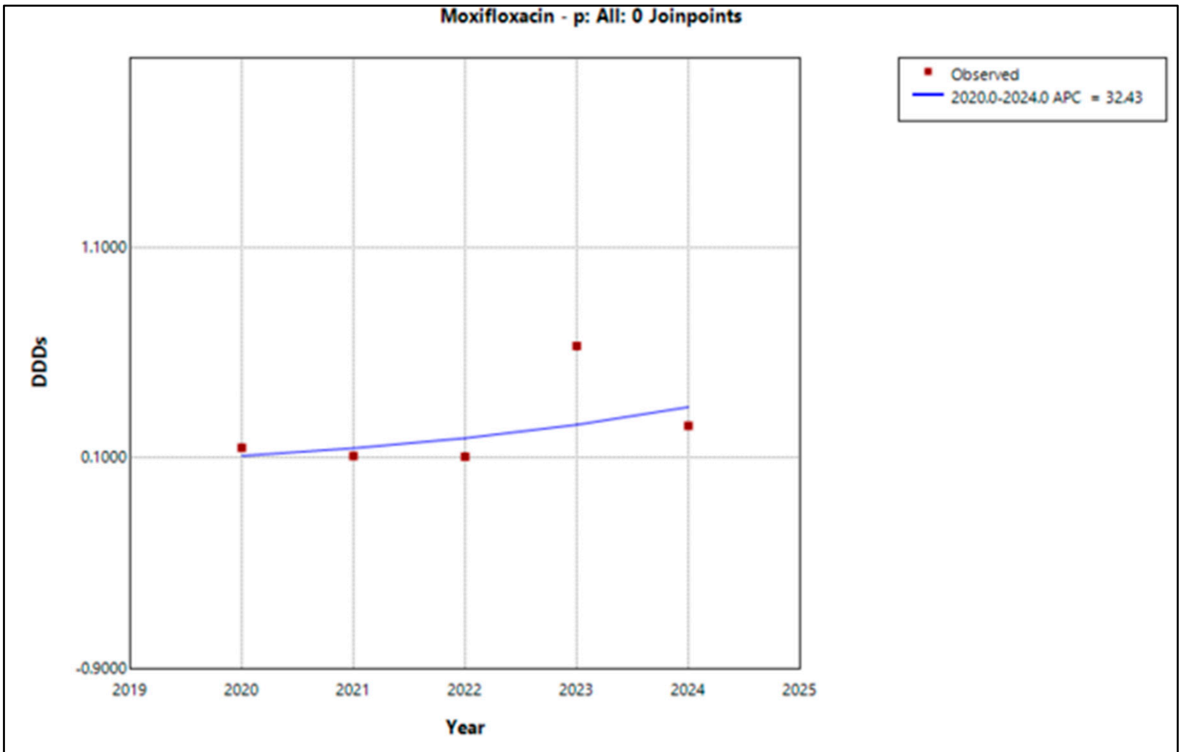

Annual Percent Change (APC)

Segment	Lower Endpoint	Upper Endpoint	APC	Lower CI	Upper CI	Test Statistic	P-Value
1	2020	2024	32.4323	-31.3467	150.2612	--	0.378324

* Indicates that the Annual Percent Change (APC) is significantly different from zero at the alpha = 0.05 level.
-- Test Statistic not available for the Empirical Quantile method. See Help to Learn More

Average Annual Percent Change (AAPC)

Range	Lower Endpoint	Upper Endpoint	AAPC	Lower CI	Upper CI	Test Statistic	P-Value
Full Range	2020	2024	32.4323	-31.3467	150.2612	--	0.378324

* Indicates that the AAPC is significantly different from zero at the alpha = 0.05 level.
-- Test Statistic not available for the Empirical Quantile method. See Help to Learn More

Figure S29. Moxifloxacin (parenteral) 2020-2024 consumption trend.

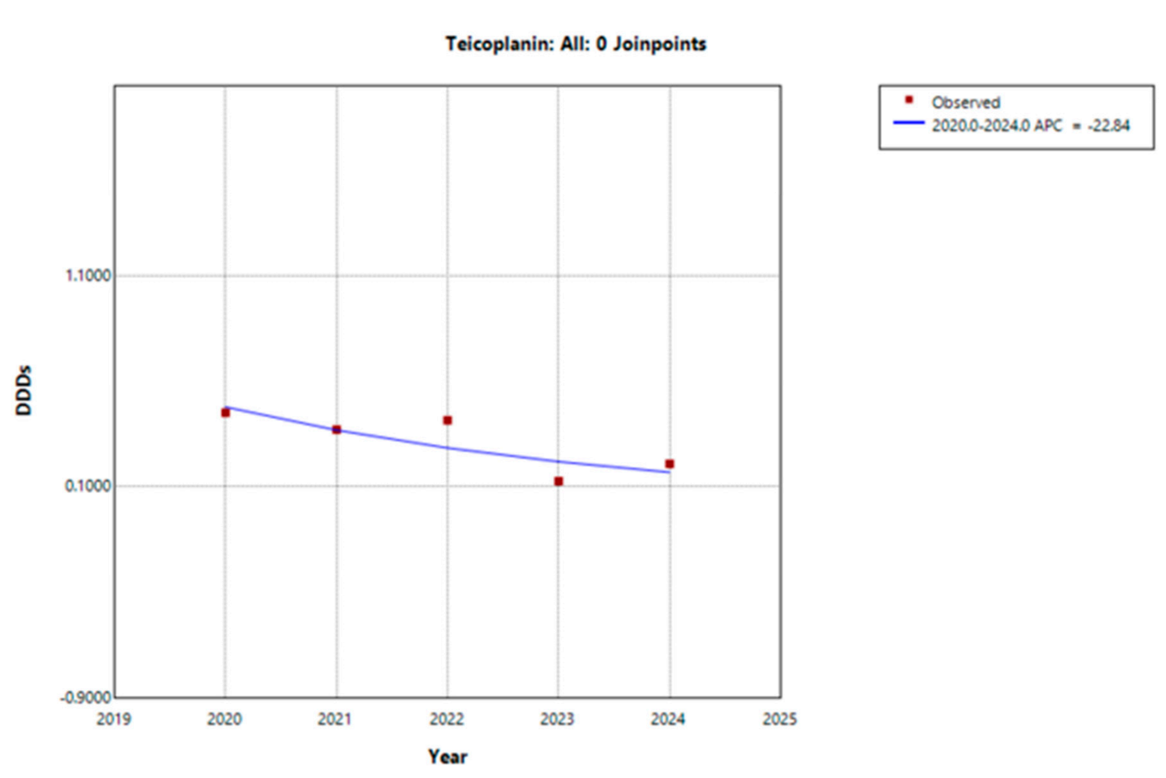

Annual Percent Change (APC)

Segment	Lower Endpoint	Upper Endpoint	APC	Lower CI	Upper CI	Test Statistic	P-Value
1	2020	2024	-22.8443	-45.4517	7.9465	--	0.137173

* Indicates that the Annual Percent Change (APC) is significantly different from zero at the alpha = 0.05 level.
-- Test Statistic not available for the Empirical Quantile method. See Help to Learn More

Average Annual Percent Change (AAPC)

Range	Lower Endpoint	Upper Endpoint	AAPC	Lower CI	Upper CI	Test Statistic	P-Value
Full Range	2020	2024	-22.8443	-45.4517	7.9465	--	0.137173

* Indicates that the AAPC is significantly different from zero at the alpha = 0.05 level.
-- Test Statistic not available for the Empirical Quantile method. See Help to Learn More

Figure S30. Teicoplanin 2020-2024 consumption trend.

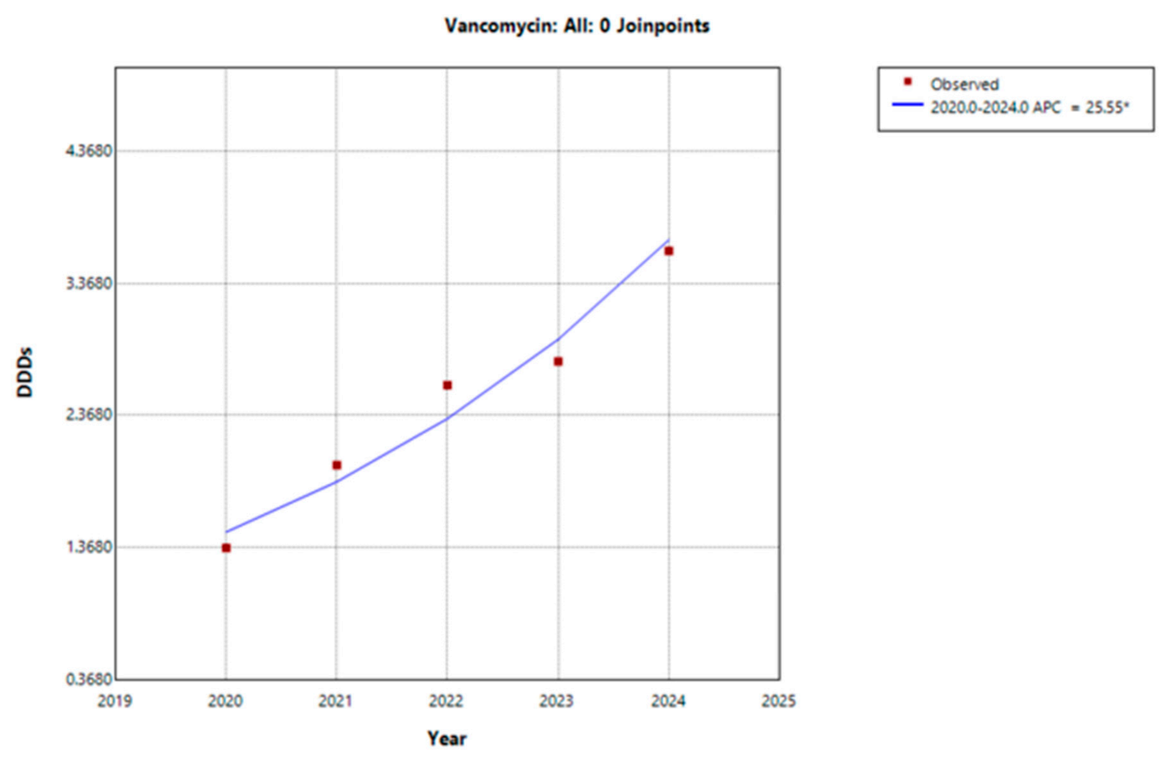

Annual Percent Change (APC)

Segment	Lower Endpoint	Upper Endpoint	APC	Lower CI	Upper CI	Test Statistic	P-Value
1	2020	2024	25.5457*	11.7009	40.6429	--	< 0.000001

* Indicates that the Annual Percent Change (APC) is significantly different from zero at the alpha = 0.05 level.
-- Test Statistic not available for the Empirical Quantile method. See Help to Learn More

Average Annual Percent Change (AAPC)

Range	Lower Endpoint	Upper Endpoint	AAPC	Lower CI	Upper CI	Test Statistic	P-Value
Full Range	2020	2024	25.5457*	11.7009	40.6429	--	< 0.000001

* Indicates that the AAPC is significantly different from zero at the alpha = 0.05 level.
-- Test Statistic not available for the Empirical Quantile method. See Help to Learn More

Figure S31. Vancomycin 2020-2024 consumption trend.

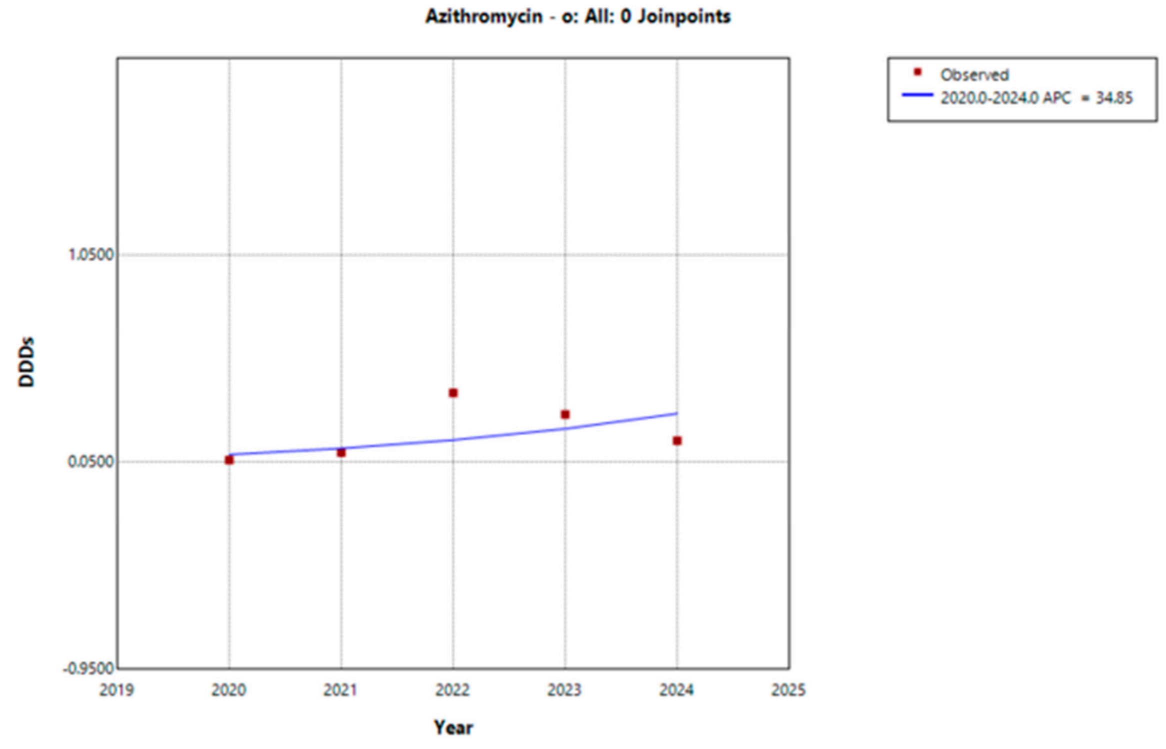

Annual Percent Change (APC)

Segment	Lower Endpoint	Upper Endpoint	APC	Lower CI	Upper CI	Test Statistic	P-Value
1	2020	2024	34.8482	-39.8053	193.6365	--	0.393121

* Indicates that the Annual Percent Change (APC) is significantly different from zero at the alpha = 0.05 level.
-- Test Statistic not available for the Empirical Quantile method. See Help to Learn More

Average Annual Percent Change (AAPC)

Range	Lower Endpoint	Upper Endpoint	AAPC	Lower CI	Upper CI	Test Statistic	P-Value
Full Range	2020	2024	34.8482	-39.8053	193.6365	--	0.393121

* Indicates that the AAPC is significantly different from zero at the alpha = 0.05 level.
-- Test Statistic not available for the Empirical Quantile method. See Help to Learn More

Figure S32. Azithromycin (oral) 2020-2024 consumption trend.

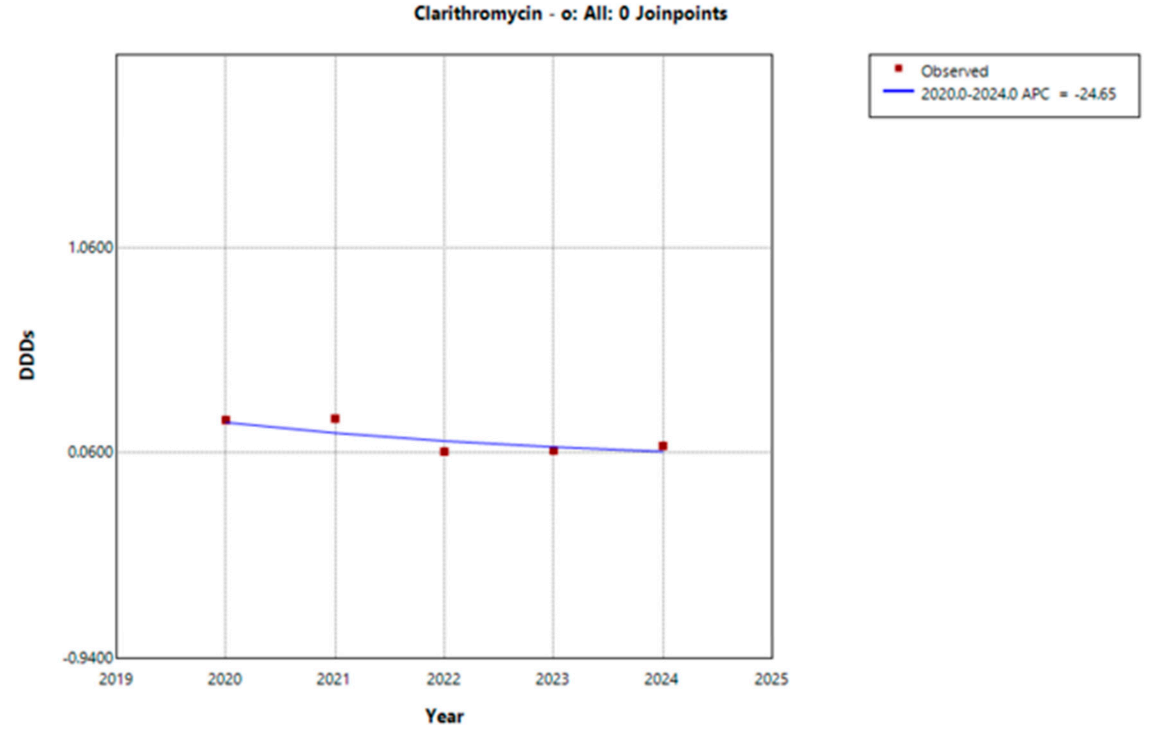

Annual Percent Change (APC)

Segment	Lower Endpoint	Upper Endpoint	APC	Lower CI	Upper CI	Test Statistic	P-Value
1	2020	2024	-24.6458	-53.1286	19.6647	--	0.220356

* Indicates that the Annual Percent Change (APC) is significantly different from zero at the alpha = 0.05 level.
-- Test Statistic not available for the Empirical Quantile method. See Help to Learn More

Average Annual Percent Change (AAPC)

Range	Lower Endpoint	Upper Endpoint	AAPC	Lower CI	Upper CI	Test Statistic	P-Value
Full Range	2020	2024	-24.6458	-53.1286	19.6647	--	0.220356

* Indicates that the AAPC is significantly different from zero at the alpha = 0.05 level.
-- Test Statistic not available for the Empirical Quantile method. See Help to Learn More

Figure S33. Clarithromycin (oral) 2020-2024 consumption trend.

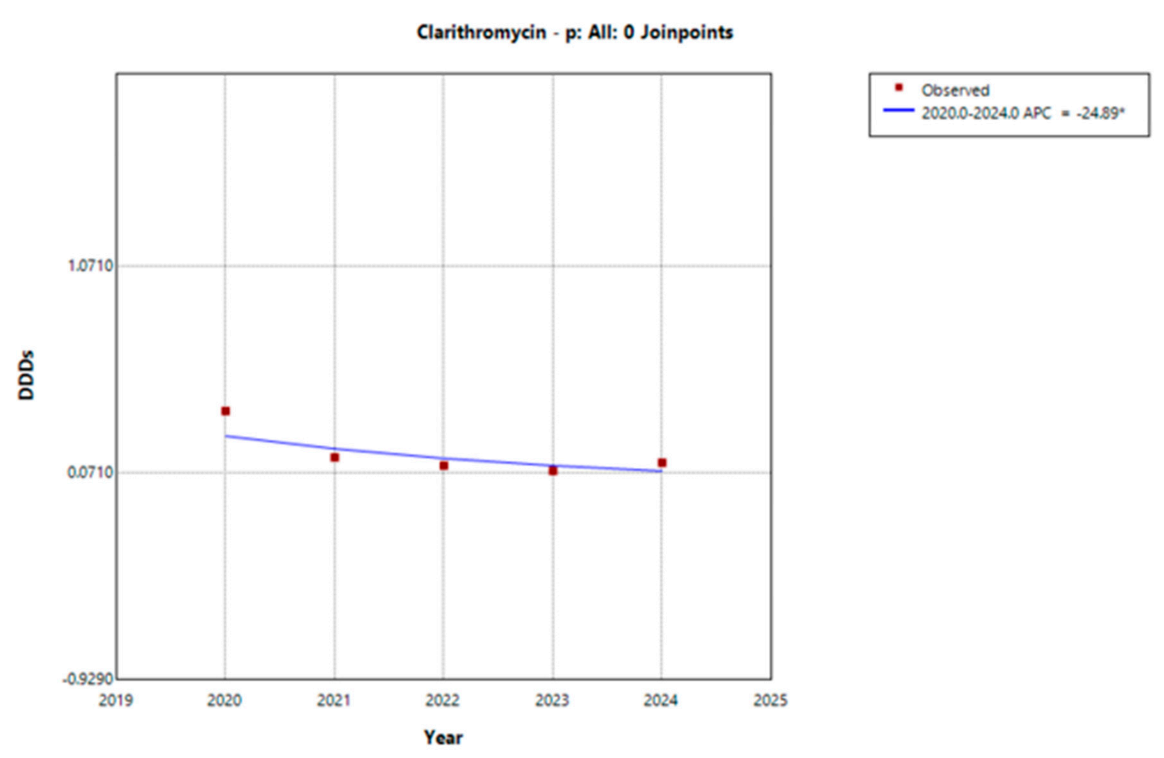

Annual Percent Change (APC)

Segment	Lower Endpoint	Upper Endpoint	APC	Lower CI	Upper CI	Test Statistic	P-Value
1	2020	2024	-24.8929*	-40.1387	-4.9446	--	0.009598

* Indicates that the Annual Percent Change (APC) is significantly different from zero at the alpha = 0.05 level.
-- Test Statistic not available for the Empirical Quantile method. See Help to Learn More

Average Annual Percent Change (AAPC)

Range	Lower Endpoint	Upper Endpoint	AAPC	Lower CI	Upper CI	Test Statistic	P-Value
Full Range	2020	2024	-24.8929*	-40.1387	-4.9446	--	0.009598

* Indicates that the AAPC is significantly different from zero at the alpha = 0.05 level.
-- Test Statistic not available for the Empirical Quantile method. See Help to Learn More

Figure S34. Clarithromycin (parenteral) 2020-2024 consumption trend.

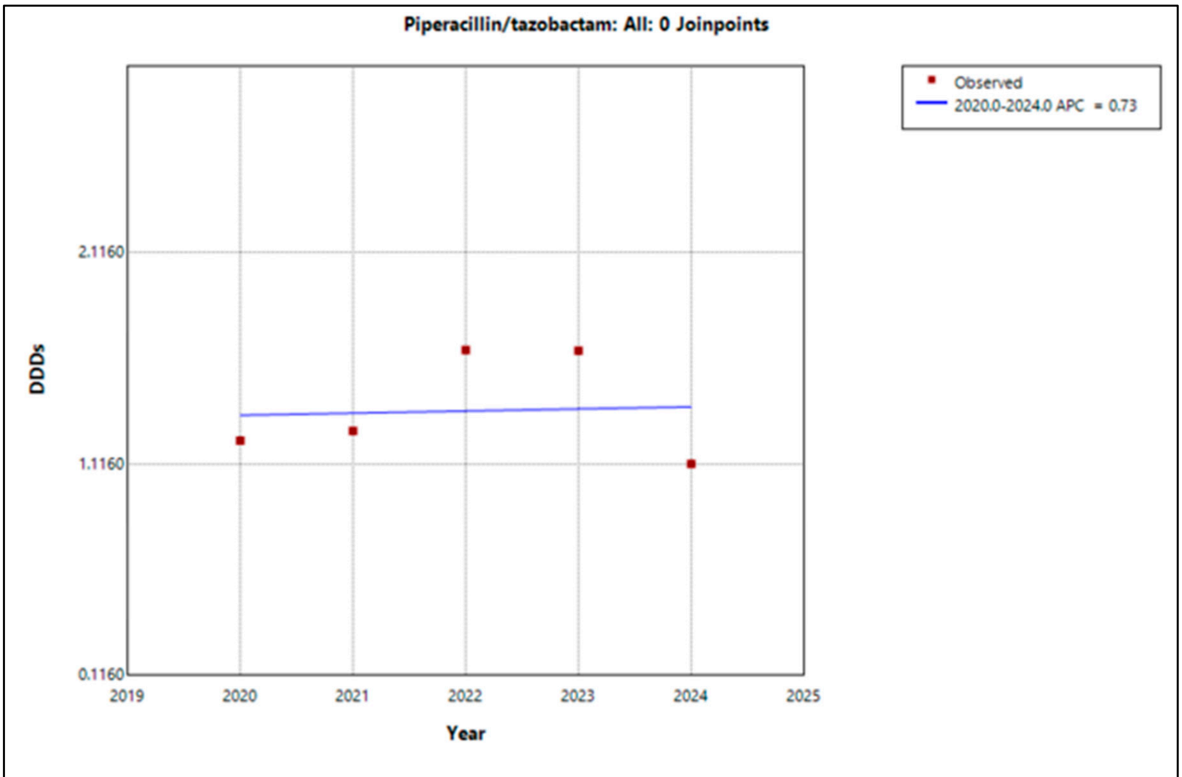

Annual Percent Change (APC)

Segment	Lower Endpoint	Upper Endpoint	APC	Lower CI	Upper CI	Test Statistic	P-Value
1	2020	2024	0.7292	-24.5463	33.9490	--	0.962208

* Indicates that the Annual Percent Change (APC) is significantly different from zero at the alpha = 0.05 level.
-- Test Statistic not available for the Empirical Quantile method. See Help to Learn More

Average Annual Percent Change (AAPC)

Range	Lower Endpoint	Upper Endpoint	AAPC	Lower CI	Upper CI	Test Statistic	P-Value
Full Range	2020	2024	0.7292	-24.5463	33.9490	--	0.962208

* Indicates that the AAPC is significantly different from zero at the alpha = 0.05 level.
-- Test Statistic not available for the Empirical Quantile method. See Help to Learn More

Figure S35. Piperacillin/tazobactam 2020-2024 consumption trend.

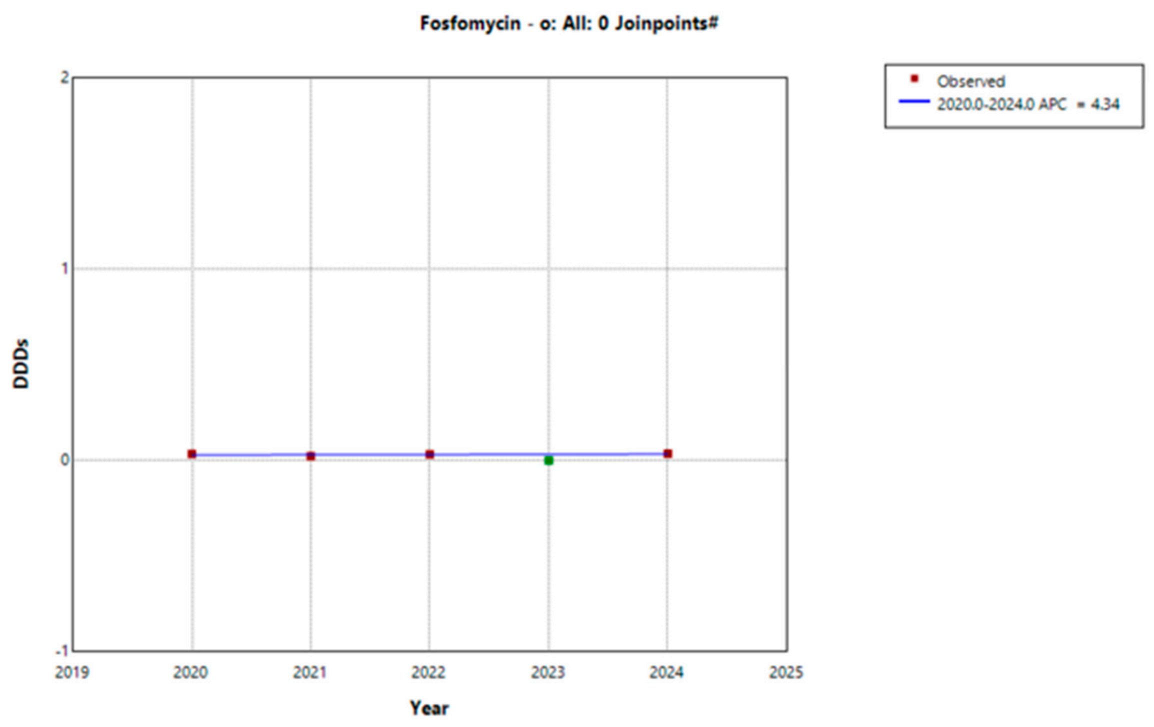

Annual Percent Change (APC)							
Segment	Lower Endpoint	Upper Endpoint	APC	Lower CI	Upper CI	Test Statistic	P-Value
1	2020	2024	4.3351	-8.6194	18.3164	--	0.557888

* Indicates that the Annual Percent Change (APC) is significantly different from zero at the alpha = 0.05 level.
-- Test Statistic not available for the Empirical Quantile method. See Help to Learn More

Average Annual Percent Change (AAPC)							
Range	Lower Endpoint	Upper Endpoint	AAPC	Lower CI	Upper CI	Test Statistic	P-Value
Full Range	2020	2024	4.3351	-8.6194	18.3164	--	0.557888

* Indicates that the AAPC is significantly different from zero at the alpha = 0.05 level.
-- Test Statistic not available for the Empirical Quantile method. See Help to Learn More

Figure S36. Fosfomycin (oral) 2020-2024 consumption trend.

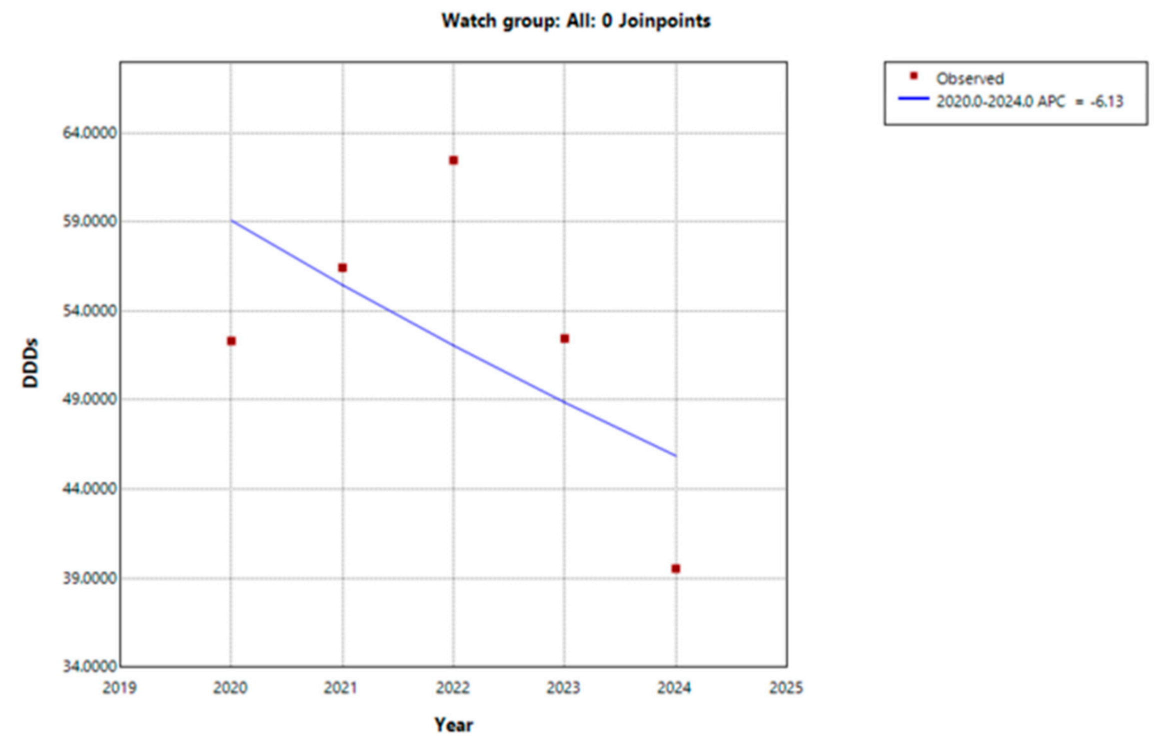

Annual Percent Change (APC)

Segment	Lower Endpoint	Upper Endpoint	APC	Lower CI	Upper CI	Test Statistic	P-Value
1	2020	2024	-6.1322	-26.0026	18.8932	--	0.433113

* Indicates that the Annual Percent Change (APC) is significantly different from zero at the alpha = 0.05 level.
-- Test Statistic not available for the Empirical Quantile method. See Help to Learn More

Average Annual Percent Change (AAPC)

Range	Lower Endpoint	Upper Endpoint	AAPC	Lower CI	Upper CI	Test Statistic	P-Value
Full Range	2020	2024	-6.1322	-26.0026	18.8932	--	0.433113

* Indicates that the AAPC is significantly different from zero at the alpha = 0.05 level.
-- Test Statistic not available for the Empirical Quantile method. See Help to Learn More

Figure S37. Watch group 2020-2024 consumption trend.

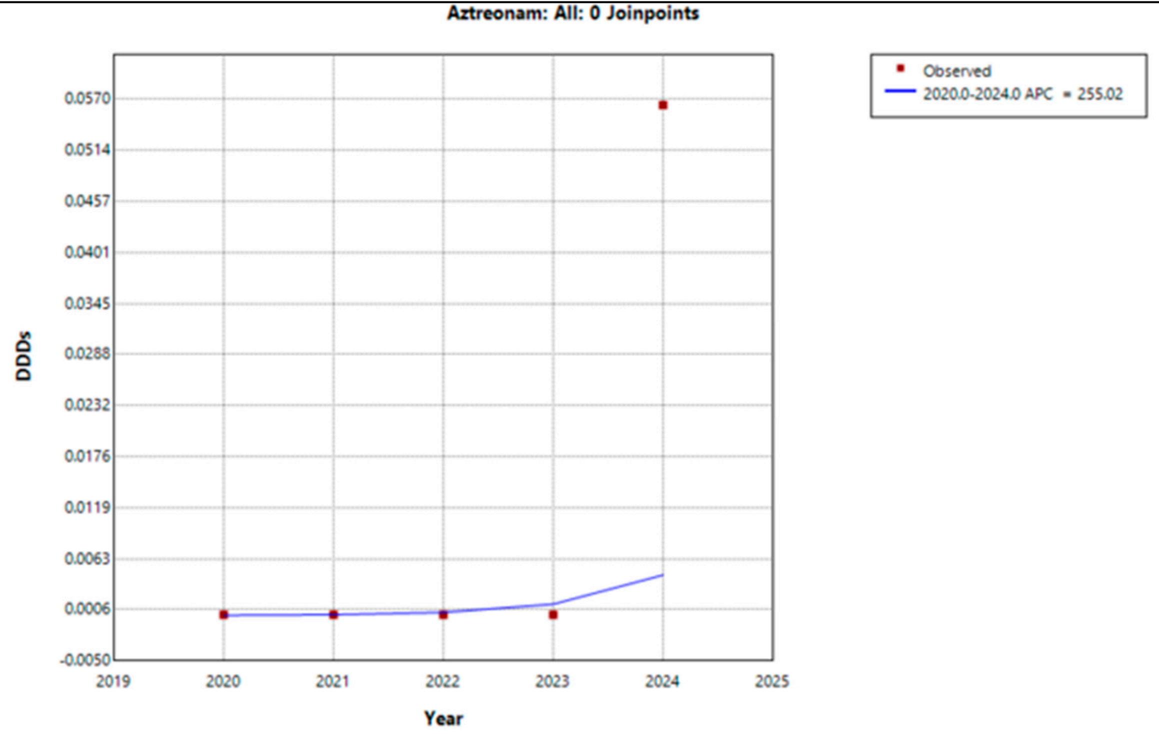

Annual Percent Change (APC)

Segment	Lower Endpoint	Upper Endpoint	APC	Lower CI	Upper CI	Test Statistic	P-Value
1	2020	2024	255.0225	-58.4921	2617.0721	--	0.233953

* Indicates that the Annual Percent Change (APC) is significantly different from zero at the alpha = 0.05 level.
-- Test Statistic not available for the Empirical Quantile method. See Help to Learn More

Average Annual Percent Change (AAPC)

Range	Lower Endpoint	Upper Endpoint	AAPC	Lower CI	Upper CI	Test Statistic	P-Value
Full Range	2020	2024	255.0225	-58.4921	2617.0721	--	0.233953

* Indicates that the AAPC is significantly different from zero at the alpha = 0.05 level.
-- Test Statistic not available for the Empirical Quantile method. See Help to Learn More

Figure S38. Aztreonam 2020-2024 consumption trend.

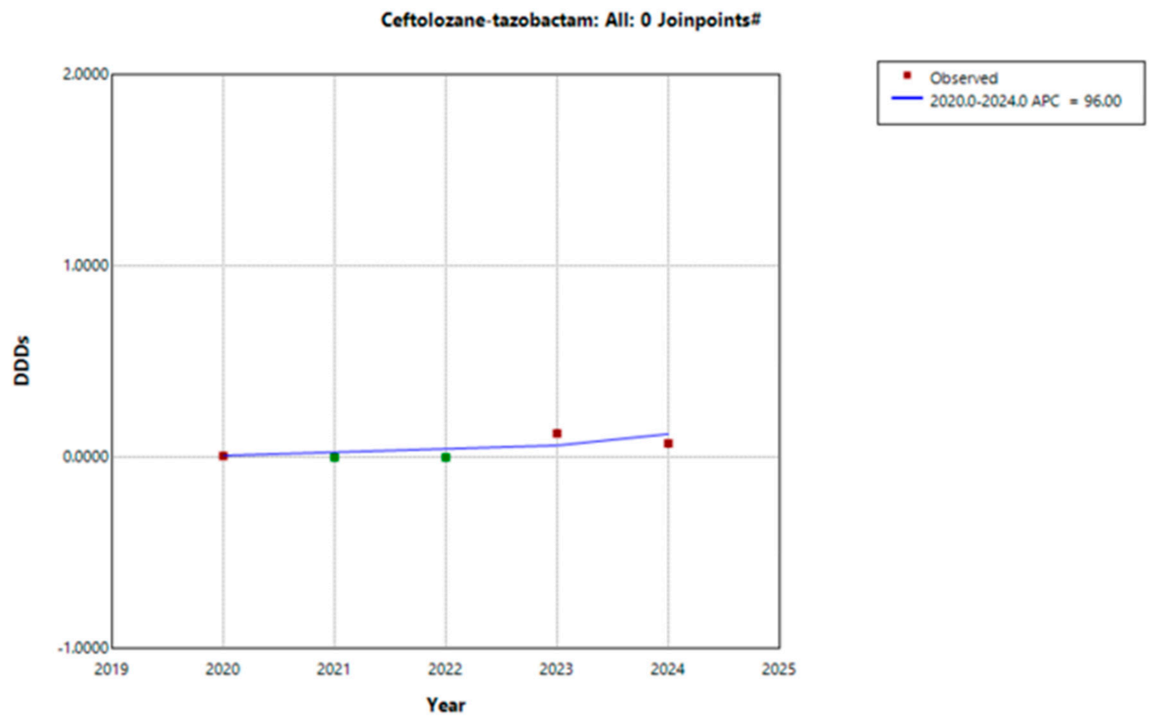

Annual Percent Change (APC)

Segment	Lower Endpoint	Upper Endpoint	APC	Lower CI	Upper CI	Test Statistic	P-Value
1	2020	2024	96.0017	-3.5196	325.6809	--	0.063587

* Indicates that the Annual Percent Change (APC) is significantly different from zero at the alpha = 0.05 level.
-- Test Statistic not available for the Empirical Quantile method. See Help to Learn More

Average Annual Percent Change (AAPC)

Range	Lower Endpoint	Upper Endpoint	AAPC	Lower CI	Upper CI	Test Statistic	P-Value
Full Range	2020	2024	96.0017	-3.5196	325.6809	--	0.063587

* Indicates that the AAPC is significantly different from zero at the alpha = 0.05 level.
-- Test Statistic not available for the Empirical Quantile method. See Help to Learn More

Figure S39. Ceftolozane/tazobactam 2020-2024 consumption trend.

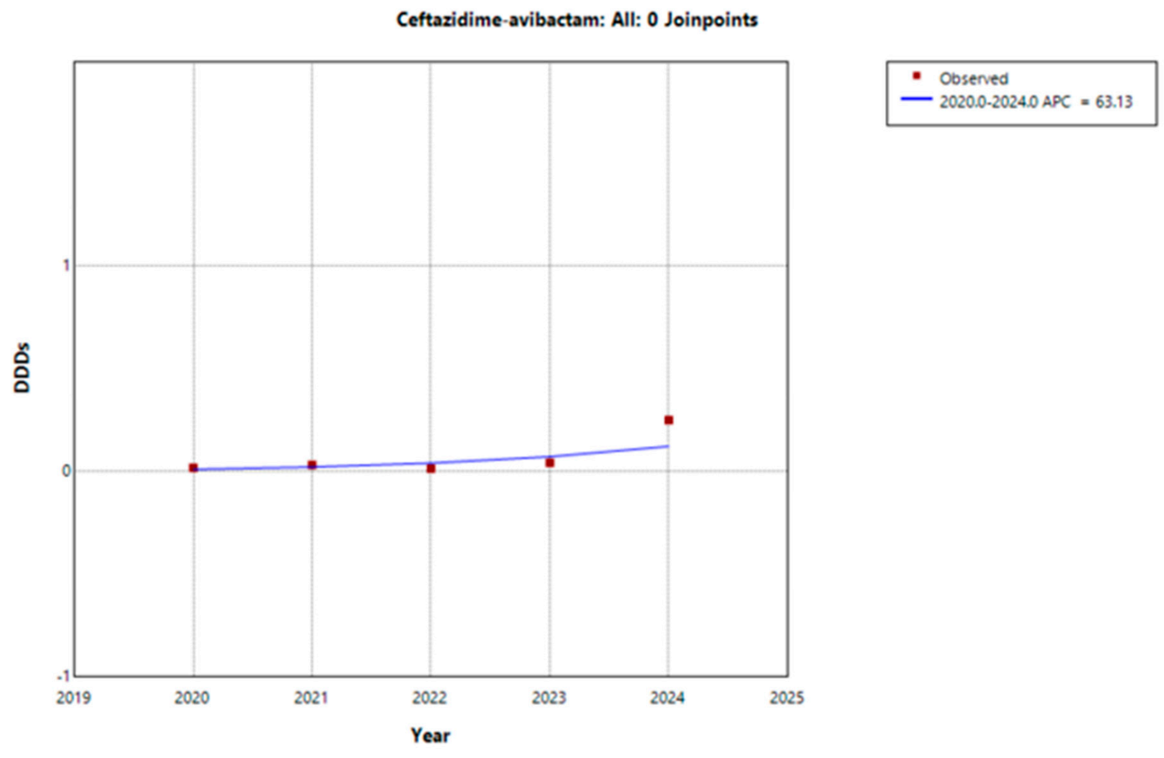

Annual Percent Change (APC)

Segment	Lower Endpoint	Upper Endpoint	APC	Lower CI	Upper CI	Test Statistic	P-Value
1	2020	2024	63.1344	-7.5043	181.0703	--	0.097181

* Indicates that the Annual Percent Change (APC) is significantly different from zero at the alpha = 0.05 level.
-- Test Statistic not available for the Empirical Quantile method. See Help to Learn More

Average Annual Percent Change (AAPC)

Range	Lower Endpoint	Upper Endpoint	AAPC	Lower CI	Upper CI	Test Statistic	P-Value
Full Range	2020	2024	63.1344	-7.5043	181.0703	--	0.097181

* Indicates that the AAPC is significantly different from zero at the alpha = 0.05 level.
-- Test Statistic not available for the Empirical Quantile method. See Help to Learn More

Figure S40. Ceftazidime/avibactam 2020-2024 consumption trend.

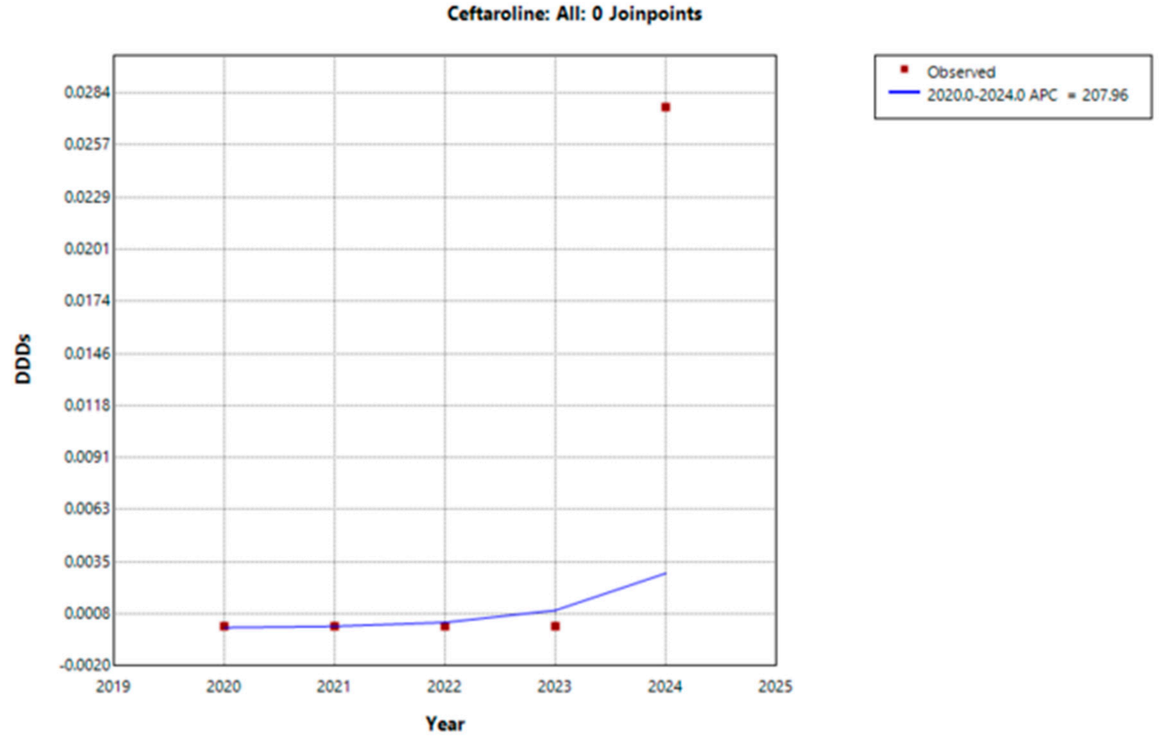

Annual Percent Change (APC)

Segment	Lower Endpoint	Upper Endpoint	APC	Lower CI	Upper CI	Test Statistic	P-Value
1	2020	2024	207.9612	-54.1868	1775.5984	--	0.233953

* Indicates that the Annual Percent Change (APC) is significantly different from zero at the alpha = 0.05 level.
-- Test Statistic not available for the Empirical Quantile method. See Help to Learn More

Average Annual Percent Change (AAPC)

Range	Lower Endpoint	Upper Endpoint	AAPC	Lower CI	Upper CI	Test Statistic	P-Value
Full Range	2020	2024	207.9612	-54.1868	1775.5984	--	0.233953

* Indicates that the AAPC is significantly different from zero at the alpha = 0.05 level.
-- Test Statistic not available for the Empirical Quantile method. See Help to Learn More

Figure S41. Ceftaroline 2020-2024 consumption trend.

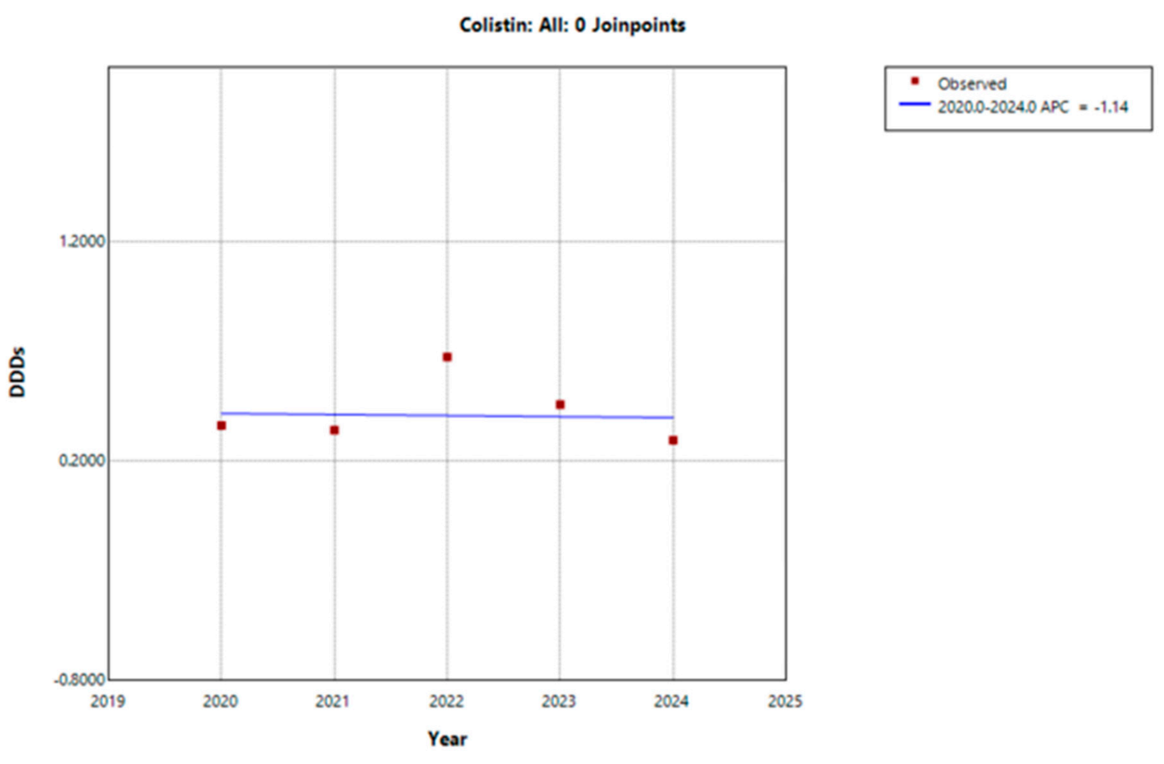

Annual Percent Change (APC)

Segment	Lower Endpoint	Upper Endpoint	APC	Lower CI	Upper CI	Test Statistic	P-Value
1	2020	2024	-1.1411	-29.4001	37.1762	--	0.910218

* Indicates that the Annual Percent Change (APC) is significantly different from zero at the alpha = 0.05 level.
-- Test Statistic not available for the Empirical Quantile method. See Help to Learn More

Average Annual Percent Change (AAPC)

Range	Lower Endpoint	Upper Endpoint	AAPC	Lower CI	Upper CI	Test Statistic	P-Value
Full Range	2020	2024	-1.1411	-29.4001	37.1762	--	0.910218

* Indicates that the AAPC is significantly different from zero at the alpha = 0.05 level.
-- Test Statistic not available for the Empirical Quantile method. See Help to Learn More

Figure S42. Colistin 2020-2024 consumption trend.

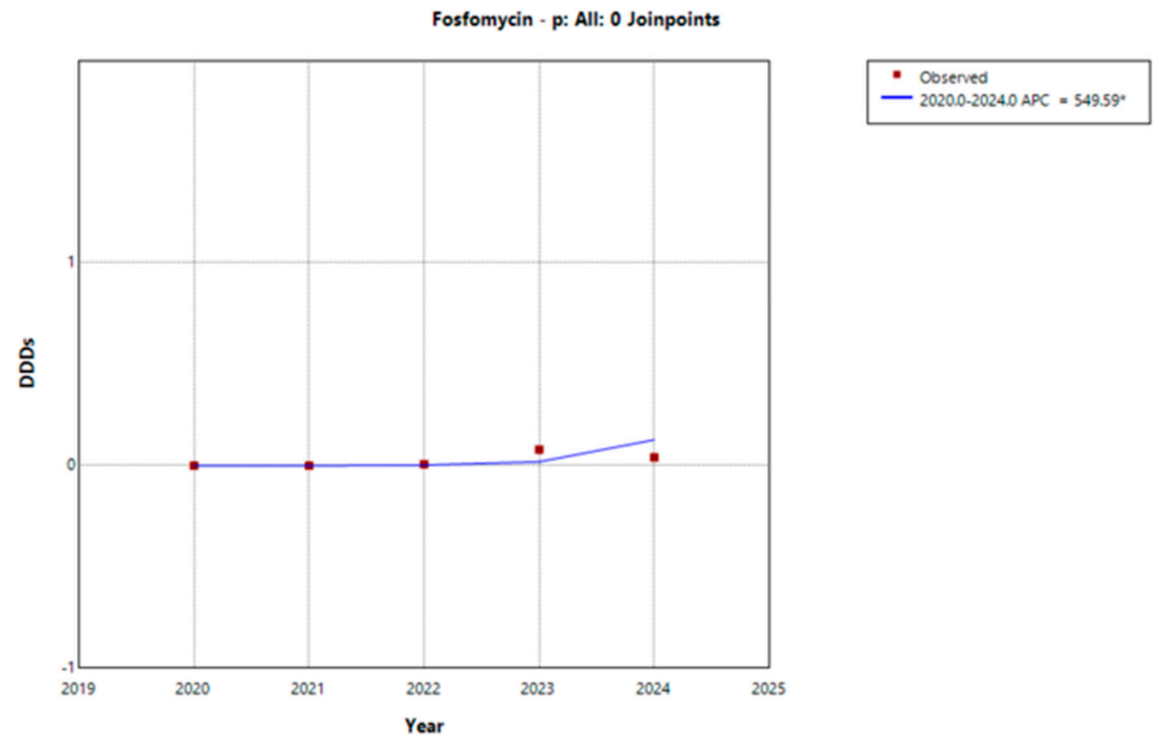

Annual Percent Change (APC)

Segment	Lower Endpoint	Upper Endpoint	APC	Lower CI	Upper CI	Test Statistic	P-Value
1	2020	2024	549.5923*	15.4675	3416.6799	--	0.029594

* Indicates that the Annual Percent Change (APC) is significantly different from zero at the alpha = 0.05 level.
-- Test Statistic not available for the Empirical Quantile method. See Help to Learn More

Average Annual Percent Change (AAPC)

Range	Lower Endpoint	Upper Endpoint	AAPC	Lower CI	Upper CI	Test Statistic	P-Value
Full Range	2020	2024	549.5923*	15.4675	3416.6799	--	0.029594

* Indicates that the AAPC is significantly different from zero at the alpha = 0.05 level.
-- Test Statistic not available for the Empirical Quantile method. See Help to Learn More

Figure S43. Fosfomycin (parenteral) 2020-2024 consumption trend.

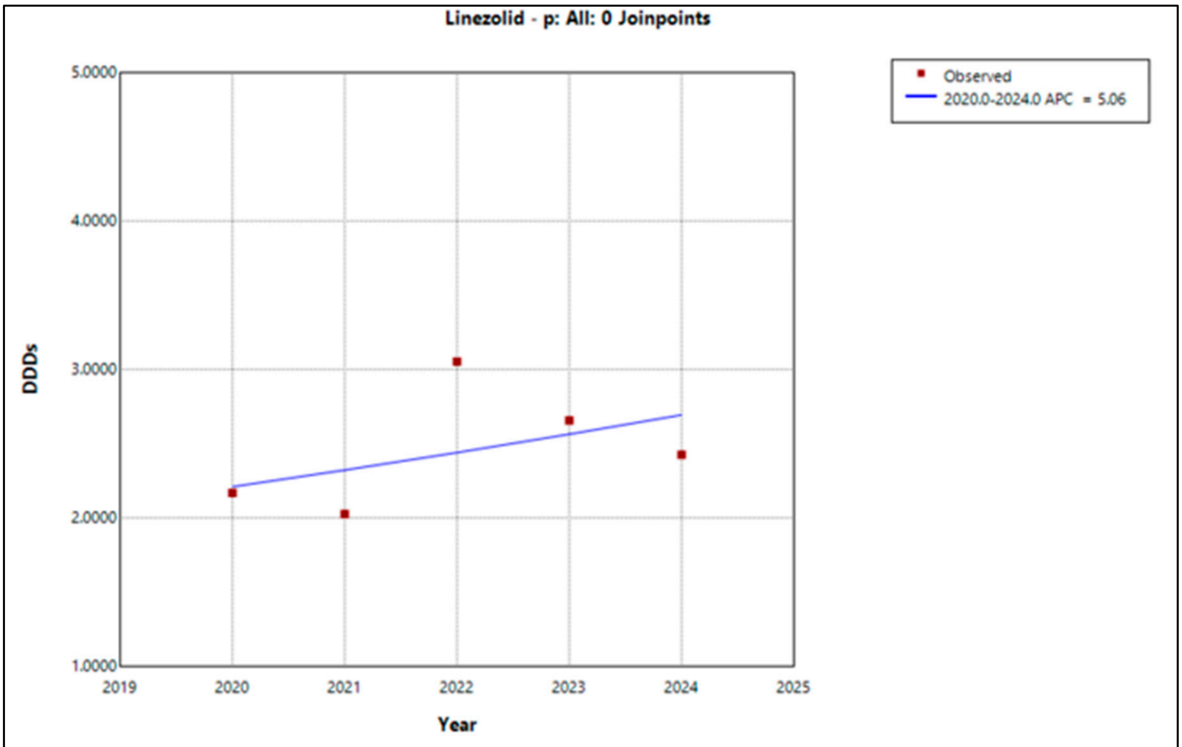

Annual Percent Change (APC)

Segment	Lower Endpoint	Upper Endpoint	APC	Lower CI	Upper CI	Test Statistic	P-Value
1	2020	2024	5.0636	-10.2461	22.1304	--	0.467506

* Indicates that the Annual Percent Change (APC) is significantly different from zero at the alpha = 0.05 level.
-- Test Statistic not available for the Empirical Quantile method. See Help to Learn More

Average Annual Percent Change (AAPC)

Range	Lower Endpoint	Upper Endpoint	AAPC	Lower CI	Upper CI	Test Statistic	P-Value
Full Range	2020	2024	5.0636	-10.2461	22.1304	--	0.467506

* Indicates that the AAPC is significantly different from zero at the alpha = 0.05 level.
-- Test Statistic not available for the Empirical Quantile method. See Help to Learn More

Figure S44. Linezolid (parenteral) 2020-2024 consumption trend.

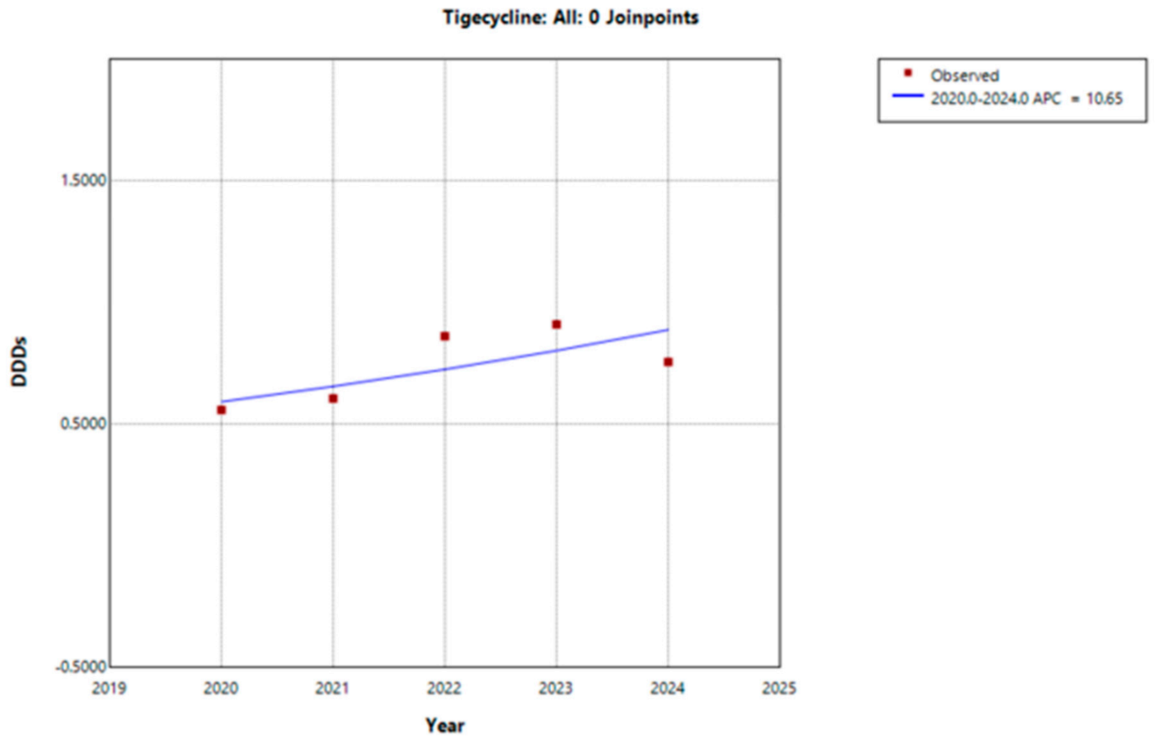

Annual Percent Change (APC)

Segment	Lower Endpoint	Upper Endpoint	APC	Lower CI	Upper CI	Test Statistic	P-Value
1	2020	2024	10.6471	-9.3403	34.3066	--	0.285143

* Indicates that the Annual Percent Change (APC) is significantly different from zero at the alpha = 0.05 level.
-- Test Statistic not available for the Empirical Quantile method. See Help to Learn More

Average Annual Percent Change (AAPC)

Range	Lower Endpoint	Upper Endpoint	AAPC	Lower CI	Upper CI	Test Statistic	P-Value
Full Range	2020	2024	10.6471	-9.3403	34.3066	--	0.285143

* Indicates that the AAPC is significantly different from zero at the alpha = 0.05 level.
-- Test Statistic not available for the Empirical Quantile method. See Help to Learn More

Figure S45. Tigecycline 2020-2024 consumption trend.

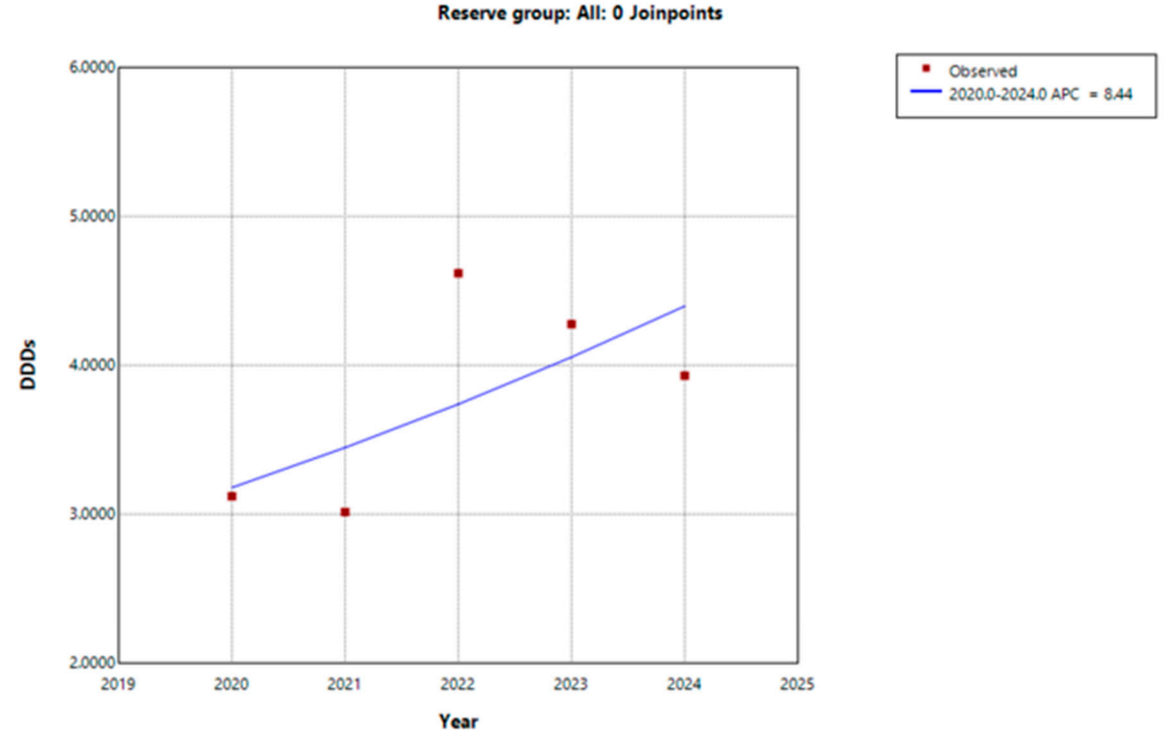

Annual Percent Change (APC)

Segment	Lower Endpoint	Upper Endpoint	APC	Lower CI	Upper CI	Test Statistic	P-Value
1	2020	2024	8.4418	-8.5762	27.7810	--	0.301540

* Indicates that the Annual Percent Change (APC) is significantly different from zero at the alpha = 0.05 level.
-- Test Statistic not available for the Empirical Quantile method. See Help to Learn More

Average Annual Percent Change (AAPC)

Range	Lower Endpoint	Upper Endpoint	AAPC	Lower CI	Upper CI	Test Statistic	P-Value
Full Range	2020	2024	8.4418	-8.5762	27.7810	--	0.301540

* Indicates that the AAPC is significantly different from zero at the alpha = 0.05 level.
-- Test Statistic not available for the Empirical Quantile method. See Help to Learn More

Figure S46. Reserve group 2020-2024 consumption trend.

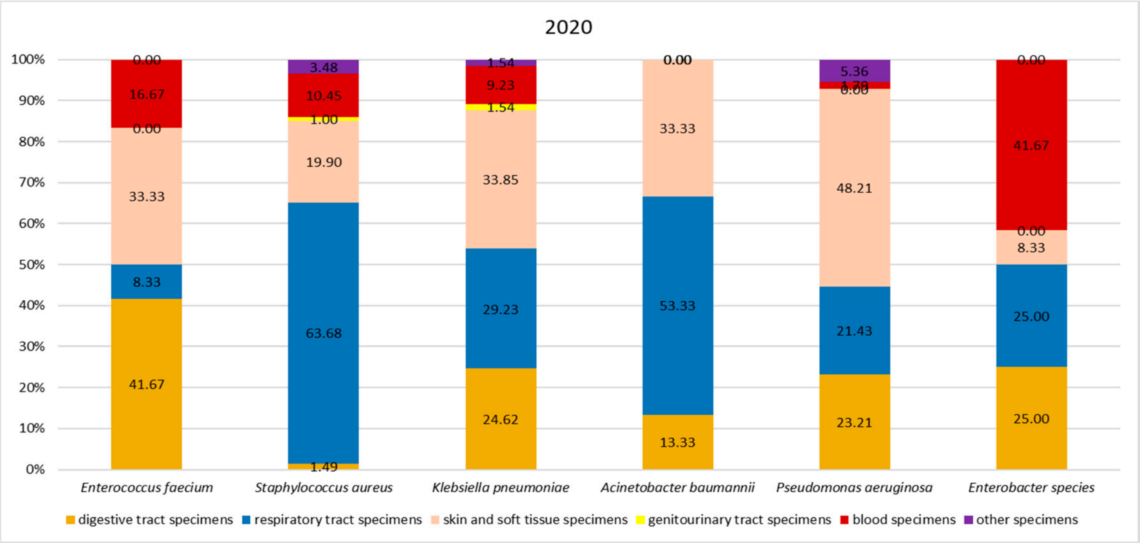

Figure S47. Distribution of positive samples for each ESKAPE pathogen in 2020.

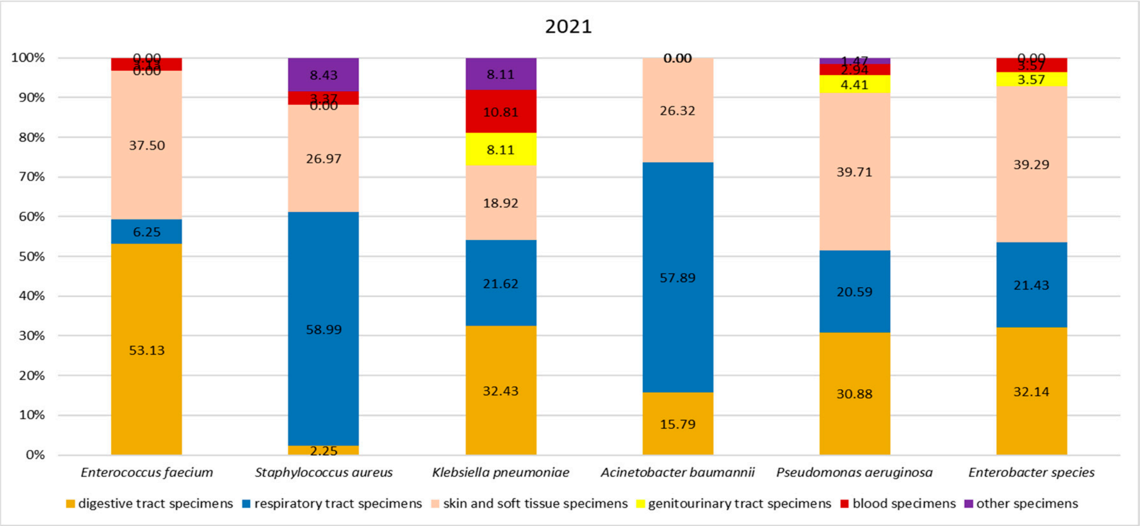

Figure S48. Distribution of positive samples for each ESKAPE pathogen in 2021.

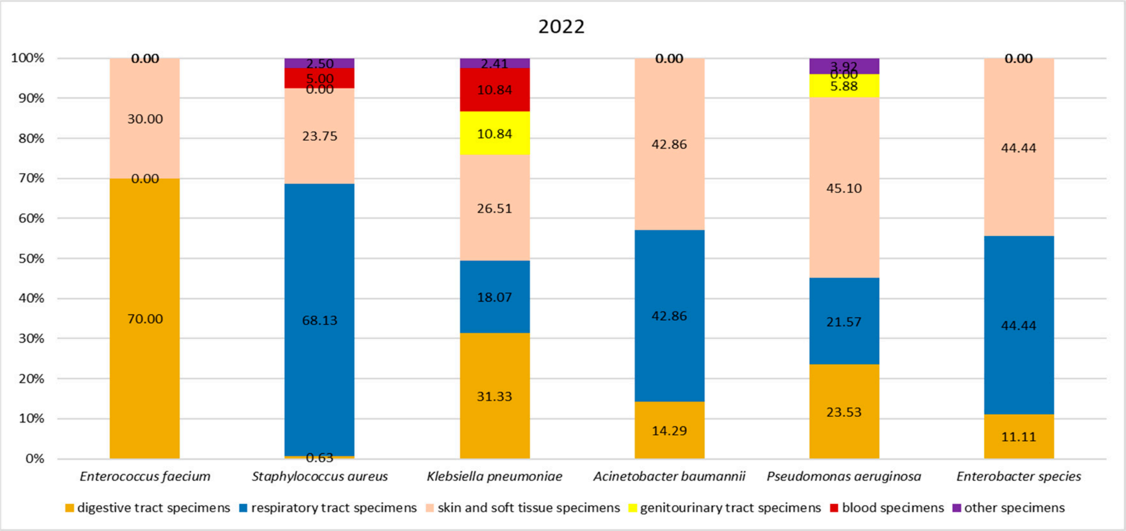

Figure S49. Distribution of positive samples for each ESKAPE pathogen in 2022.

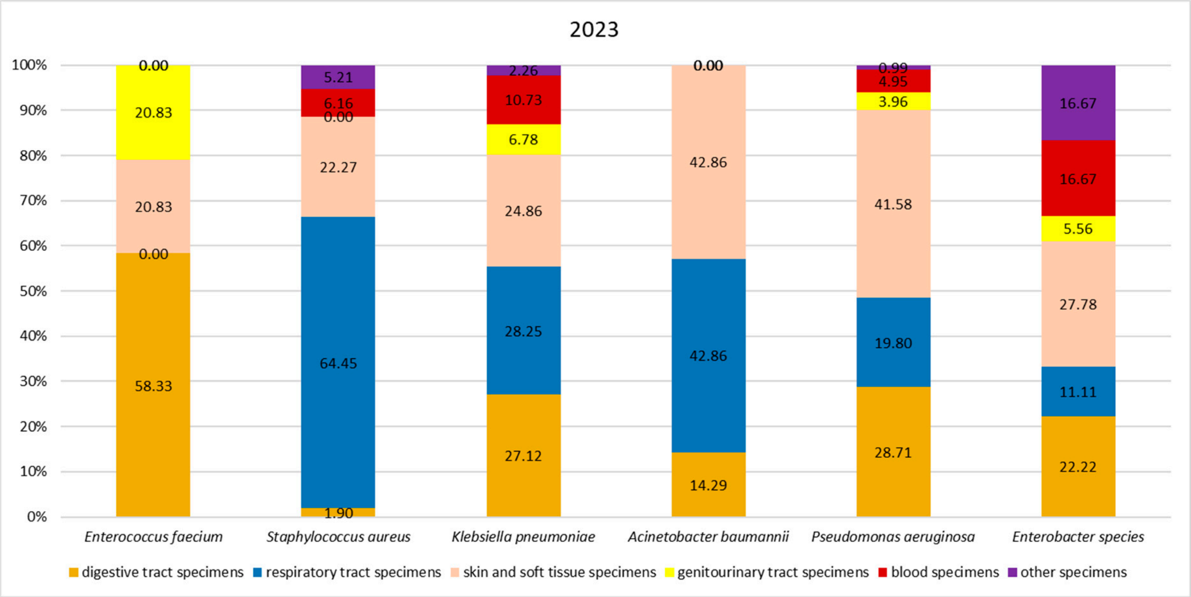

Figure S50. Distribution of positive samples for each ESKAPE pathogen in 2023.

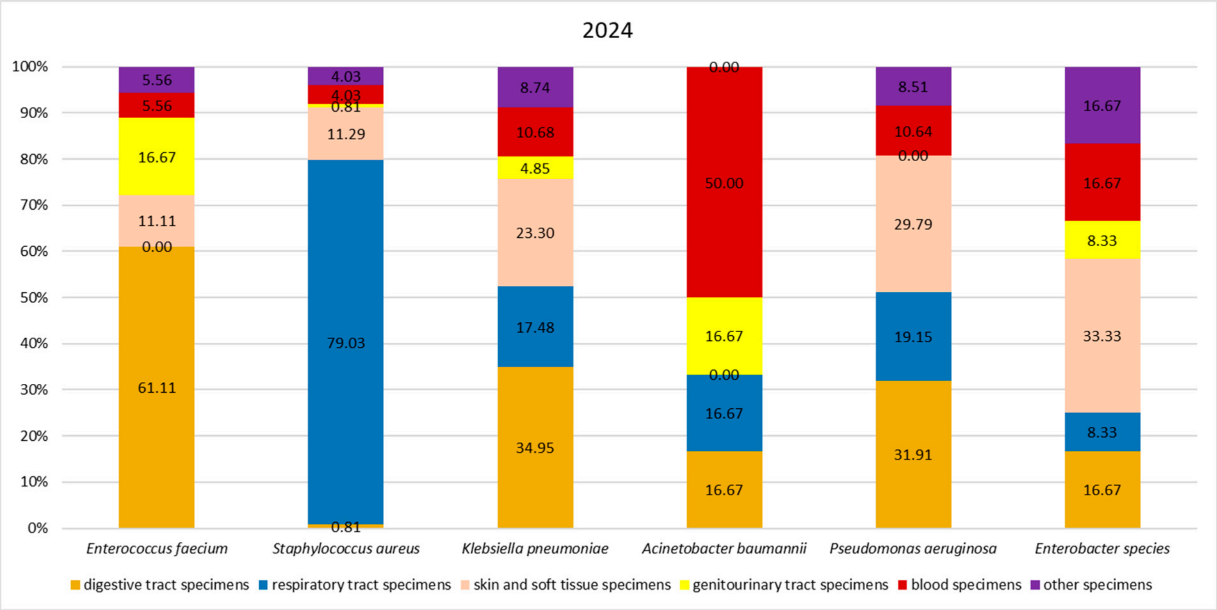

Figure S51. Distribution of positive samples for each ESKAPE pathogen in 2024.
